# Intersectional equity in Brazil’s remote rural municipalities: the road to efficiency and effectiveness in local health systems

**DOI:** 10.3389/fpubh.2024.1401193

**Published:** 2024-09-10

**Authors:** Simone Schenkman, Aylene Bousquat

**Affiliations:** Department of Politics, Management and Health, Faculty of Public Health, University of São Paulo, São Paulo, Brazil

**Keywords:** equity, rural health, primary health care, intersectionality, efficiency, effectiveness, data envelopment analysis, fixed effects models

## Abstract

**Objective:**

The Brazilian remote rurality has been classified more reliably only recently, according to demographic density, proportion of urban population, and accessibility to urban centers. It comprises 5.8% of the municipalities, in nearly half of the states, with a population of 3,524,597 (1.85%). Remote rural localities (RRL) have reduced political/economic power, facing greater distances and barriers. Most health strategies are developed with the urban space in mind. We aim to understand how RRL are positioned concerning efficiency/effectiveness in health, compared to other urban-rural typologies of Brazilian localities, focusing on Primary Health Care (PHC), and its organizational models.

**Methods:**

We evaluated the efficiency and effectiveness of the organizational models using the health production model, from 2010–2019, gradually deepening the immersion into the RRL reality. We analyzed the human and financial resources dimensions, emphasizing teams, the results of PHC actions, and health levels. We used the fixed effects model and data envelopment analysis, cross-sectioned by intersectional inequities. We compared the Brazilian states with and without RRL, Brazilian municipalities according to rural-urban typologies, and RRL clusters.

**Results:**

Brazilian RRL states show superior resource/health efficiency through services utilization according to health needs. The remote rural typology demonstrated greater efficiency and effectiveness in health than the other typologies in the RRL states. The organizational models with the Family Health Strategy (FHS) teams and the Community Health Worker (CHW) visits played a key role, together with local *per capita* health expenditures and intergovernmental transfers. Thus, financial resources and health professionals are essential to achieve efficient/effective results in health services. Among the RRL, the Amazon region clusters stand out, denoting the importance of riverine and fluvial health teams, the proportion of diagnostic/treatment units in addition to the proportion of illiteracy and adolescent mothers along with the inequity of reaching high levels of schooling between gender/ethnicity.

**Conclusion:**

Hopefully, these elements might contribute to gains in efficiency and effectiveness, prioritizing the allocation of financial/human resources, mobile FHS teams, availability of local diagnosis/treatment, and basic sanitation. Finally, one should aim for equity of gender/ethnicity in income and education and, above all, of place, perceived in its entirety.

## 1 Introduction

Remote rural localities should be acknowledged according to the dynamics of their underlying socio-spatial relations, their social structures, and power relations, whose constant disputes bring material and symbolic consequences, even influencing how a remote rural space is conceived and valued locally and globally (1).

In Brazil, municipalities with these characteristics were classified more reliably to their reality recently by the National Institute of Geography and Statistics-IBGE (2), according to demographic density, proportion of urban population, and accessibility to urban centers. These localities are the rural remote municipalities (*n* = 323) of 13 Brazilian states (out of a total of 27 units), adding up to 5.8% of the municipalities, with a population of 3,524,597 (1.85%), differentiated from the other urban-rural typologies (urban, the adjacent rural and the intermediate remote or adjacent).

Moreover, remote rural localities differ from rural areas, with smaller and more cohesive populations but with reduced power, longer distances, and social and geographical barriers (3). These localities are not homogeneous; neither are their populations, which have multiple compositions and activities, far from performing only activities in the countryside (4, 5).

Furthermore, most public policies are still formulated based on the urban space; thus, decision-makers analyze the rural space in opposition to the urban space in a decontextualized way. The specific populations of these locations, especially the more remote ones, are even less considered in policy formulation, making it hard to implement them in remote rural locations because they deal with homogeneous policies focused on the urban space and its populations. These populations present greater social vulnerability and constitute the traditional peoples of the fields, waters, and forests, seldom studied (5).

As a counterpoint, we have specific studies and analyses on the health policies of Primary Health Care (PHC), with the research group *PHC in networks* dedicating itself to the theme; since the mid-2000s, bringing together researchers from various Brazilian educational and research institutions. More recently, the project “PHC in remote rural territories” (2018) seeks to recognize the characteristics of the supply of primary care services and their relationship with the network to ensure comprehensive and integrated care, aiming for local solutions to improve healthcare access and quality (5).

In this line, the best healthcare model is one whose logic, in the technical dimension of work processes and health practices, combines the best techniques and technologies to solve health problems according to the health needs of the population (6, 7), in an equitable, effective and efficient way. PHC, mainly through the Family Health Strategy (FHS), offers the best response to these needs, especially in remote rural settings that are distant from large urban centers and have high population rarefactions.

Multiprofessional PHC teams are expected to connect with families in the territory under their responsibility, offer and integrate individual and collective actions, provide the first contact, take responsibility for referral to other points in the system, navigation through health services, and continued care of patients, especially with health surveillance actions and the efforts of Community Health Workers (CHW) (8). We have 51,369 FHS teams (80% PHC coverage; 2023) and 257,061 CHW (61% coverage; 2020).

More importantly, the model should allow access and accessibility, either by facilitating the traveling needs of the population to the PHC facility, through institutional transportation, or in a virtual way with different means of communication—telehealth (9)—or even by moving the units and or health professionals towards the population that lives farther from the headquarters, such as those that live by the riverside or inside the forests (10). Their main contact with health services is through CHW home visits and with the health professionals that eventually organize local activities. Thus, fluvial and riverine teams designed especially for their needs are essential in this outreach, as well as fluvial PHC facilities that overcome distances and short local supplies and other means of transportation, such as ambulances and speedboats.

From the perspective of proper (effective) PHC, an integrative international review conducted by Franco et al. (11), defines three basic categories to delineate strategies adapted to rural and remote localities: access, health organization, and workforce. Access related to geographic aspects, users’ travel needs, and access to more complex services; the healthcare organization highlighted the operation of health services and community management, the physical structure, and critical supplies. Concerning the health workforce, the professional profile, role, and the factors of attraction and retainment stood out. The authors highlighted the importance of these cross-cutting axes: community action, extension and visitation models, information and communication technologies, access to care, and training and professional development.

Added to these, other relevant dimensions to PHC outlined by de Almeida et al. (12), which discuss the organization of PHC practices, being more successful, the greater its proximity and centrality. The authors advise us to consider the adscription of the clientele and territorialization, the organization of work processes, the first contact of the service, and interprofessional work. The critical points refer to distances and involve the availability of health transportation, information, and communication technologies, and strategies to attract and retain professionals.

### 1.1 Literature review: a brief overview

Despite the growing production of knowledge in these locations, most health strategies are still developed with the urban space in mind, ignoring rural locations and their specific populations, having to adapt them alone, without the corresponding resources. On another note, there are few studies that measure efficiency in PHC (13, 14), and even rarer are those that consider the inequities and local health systems of remote rural spaces. Some qualitative studies mention difficulties in allocative efficiency when showing disparities and imbalances between resources, especially human and logistic, when compared to urban settings (15). As a consequence, patients have poor access to healthcare, especially to higher-level facilities.

This trend is also observed in international settings with remote rurality, where health inequality is a global issue. For instance, Zhu et al. (16) describe how Asian-Pacific countries are having difficulties in retaining human resources in rural remote contexts, due to better wage offers in the private sector, located in urban settings. The incentives for attracting and retaining health professionals should include education, financial and personal support, with a strong PHC guidance and referral system.

In Canada, Wong and Regan (17) studied patient perspectives on PHC in rural communities, finding that the main problems were difficulties in access, continuity of care, and efficiency of the health system and services, specifically the diagnostic and therapeutic services. The traveling needs and expenses were frequently reported as a burden due to lack of organization and resources. Mseke et al. (18) conducted a scoping review in OECD countries and confirmed the impact of distances on healthcare access; mainly the time spent traveling seeking specialist medical care.

Kontodimopoulos et al. (19) actually measured productive efficiency in Greece for hospital health centers providing primary and secondary care in remote rural localities. Their findings suggest that services providing preventive medicine were more efficient regarding service utilization and hospital admissions. Also, they ponder on the needs to balance equity and efficiency in these settings. Likewise, Shen et al. (20) describe the need to enhance the quality and scope of PHC outpatient visits in remote localities, considering the health service utilization disparities in western China, where residents are provided with more medical services close to their homes. Both studies suggest that telemedicine and PHC may increase efficiency in these localities.

Mitton et al. (21) performed a broad review on the innovations on health service organization and delivery in the northern Arctic remote rural regions. Their main findings related to organizational structure of health services; utilization of telehealth and e-health; medical transportation; and public health challenges. The initiatives included operational efficiency and integration, access to care, organizational structure, public health, continuing education and workforce composition, which may positively impact health care quality and outcomes.

Regarding access and quality of care, Ferreira et al. (22) found evidence in Portuguese public hospitals that technical efficiency is predicted by access to health care services, patients’ clinical safety, appropriateness and timeliness. Quality and access work in the same direction as efficiency, especially with the association between primary and secondary health care. Ferreira et al. (23) also analyzed multiple criteria for satisfaction in a Portuguese pediatric inpatient service. Although it relates to the secondary level of care, some aspects are essential in PHC as well, such as the health professional’s communication skills, clinically and concerning the capacity to explain the users’ rights and duties, as well as the auxiliary staff’s efficiency and concern, such as the CHW.

Nunes and Ferreira (24) evaluated the efficiency and effectiveness of health before and during the COVID-19 pandemic: although efficiency dropped in the beginning of the pandemic, the effectiveness increased during the pandemic, demonstrating the sustainability and resilience of health services and professionals, and the importance of collective actions. Ferreira et al. (25) studied an optimal model for increasing the allocative efficiency of Portuguese public hospitals, according to the different modes of funding and contracts, which may tackle the inequities on access to health care and improve the quality of services, with better outcomes.

Newberry and Mallete (26) presented a pilot project that could impact health outcomes and patient experience in Canada, with Rural Health Hubs and Patient Medical Home concepts that intertwine improved patient experiences/navigation, population outcomes and system efficiency. In short, most of the international literature complies with the need for efficiency, equity or both, but few of them measure it and when they do, they do not evaluate this balance in the model, nor do they perform efficiency analysis of local health systems. On the contrary, they all outline the importance of formulating health policies according to sociogeographical characteristics, imbalances between resources, especially human and logistic, when compared to urban settings (15). As a consequence, patients have poor access to healthcare, especially to higher-level facilities.

From the brief literature review around the globe, we may affirm that place constitutes a relevant intersectional category, with urban dominance and forms of oppression in rural social space. This category adds to the other intersectional categories (27), such as gender, ethnicity, and social position, accumulating inequities. The complex notion of place has attracted much attention from geographers who describe it as a bounded entity containing unique characteristics within which people shape deep connections and identities (28). This description means that place is doubly constructed, physically but socially interpreted, narrated, perceived, felt, understood, and imagined. Accordingly, socio-environmental space is inseparable, operating simultaneously with the systems of objects and actions, time and totality (29).

Thus, places are not just physical constructs but are steep in social aspects. While places have unique meanings for people, personal history and experiences will influence their perceptions and experiences about places; at the same time, places will affect their opportunities and activities. Therefore, places interconnect in complex and unequal ways through social power relations (28).

Intersectionality encourages critical reflection that allows researchers and decision-makers to move beyond the singular categories that are typically favored in policy analysis to consider the complex relationships and interactions between gender, ethnicity, and social class, in addition to other social situations and identities, such as Indigeneity, sexuality, gender expression, immigration status, age, ability, and religion. This framework allows for examining the simultaneous impact and resistance to systems and structures of oppression and domination, such as racism, classism, sexism, ableism, and heterosexism. Thus, intersectional equity points to improving the living conditions and health status of different categories in an interrelated way (30). Achieving intersectional equity means reducing and ultimately eliminating disparities in health and its determinants that harm and affect excluded or marginalized groups (31).

To better understand the intersectional inequities in these localities and verify the best organizational models to reduce them, we aim to analyze the efficiency and effectiveness of the remote rural local health systems compared to other typologies of Brazilian localities. Thus, we intend to provide elements that can contribute to planning health actions and programs that serve these social groups in their territory, reducing the profound inequities of Brazilian society.

## 2 Methods

We evaluated the efficiency and effectiveness of different organizational models (arrangements of PHC teams and establishments) using the health production model (32) in remote rural socio-environmental spaces. We assessed the dimensions of human, material, and financial resources, emphasizing teams, the results of PHC health actions, and the final results (variables detailed in Table 1). The methodology used was the Fixed Effects (FE) model and Data Envelopment Analysis (DEA) (32), with the dimension of intersectional inequalities transversely evaluated.

**Table 1:** Variables in national comparisons, for Brazilian states and remote rural localities, according to the stage of the health production process, data sources and periods analyzed.

Dimensions	Variables	Period 1 (*t*)	Period 2 (*t* + 1)
Input			
Financial resources	Total health expenditure *per capita*	2010 IBGE	2019 STN/MF
	GDP composition	2010 IBGE	2019 STN/MF
	Public finance (taxes and transfers)	2010 IBGE	2019 STN/MF
	Health spending as a % of GDP	2010 IBGE	2019 STN/MF
	Primary health care (PHC) expenditure	2010 SIOPS/MS e-gestor-AB	2019 SIOPS/MS e-gestor-AB
Human, material and technological resources	Density of health professionals	2010 CNES	2019 CNES
	Number of PHC doctors and nurses	2010 CNES	2019 CNES
	Number and density of PHC facilities	2010 CNES	2019 CNES
	Number and density of other services and mobile units	2010 CNES	2019 CNES
	FHS (types of teams) and CHW coverage	2010 CNES e-gestor-AB	2019 CNES e-gestor-AB
	Density of hospital beds/equipment	2010 CNES	2019 CNES
Output			
Access and use	Consultations (per inhabitants)	2010 SAI	2019 SAI
	PHC consultations/home visits	2010 SIAB	2019 SISAB
	Hospital admissions (per 100 inhabitants)	2010 SIH	2019 SIH
	Prenatal consultations—consultation coverage	2010 SINASC	2019 SINASC
Prevention	Incidence of tuberculosis and HIV	2010 SINAN	2019 SINAN
	Vaccination—vaccination coverage	2010 PNI	2019 PNI
	Cervical cancer screening	2010 SISCOLO	2019 SISCOLO
	Breast cancer screening	2010 SISMAMA	2019 SISMAMA
^*^PHC (intermediate results)	% of adult mothers and newborns with normal weight^*^	2010 SINASC	2019 SINASC
	Hospitalizations for PHC and basic sanitation sensitive conditions^*^	2010 SIA/SIH	2019 SIA/SIH
	Relationship between use and need	2013 PNS	2019 PNS
Outcomes			
Final results	Life expectancy at birth	2010 IBGE	2016 IBGE
	Infant mortality	2010 SIM/SINASC	2016 SIM SINASC
	Deaths—preventable causes	2010 SIM	2015 SIM
Environmental/intersectoral			
Demographic and socioeconomic	Fertility rate	2010 IBGE	2019 IBGE
	Dependency ratio	2010 IBGE	2019 IBGE
	Population density	2010 IBGE	2019 IBGE
	Gini index of *per capita* household income	2010 IBGE	2019 IBGE
	% Bolsa Família (cash transfers)	2010 MDS	2019 MDS
	Municipal HDI	2010 UNDP	2019 UNDP
	High level of education (gender and ethnicity)	2010 IBGE	2018 IBGE
	Average income (gender and ethnicity)	2010 IBGE	2018 IBGE
	Unemployment rate	2010 IBGE	2018 IBGE
	Aging rate	2010 IBGE	2018 IBGE
Environmental risk factors and chronic diseases	Obesity and sedentary lifestyle (prevalence)	2013 PNS	2019 PNS
	Alcohol and tobacco (prevalence)	2013 PNS	2019 PNS
	Arterial hypertension and diabetes (prevalence)	2013 PNS	2019 PNS
	Adequate sanitation—proportion of population served by sewage or septic tanks, garbage collection and water supply	2010 IBGE	2018 IBGE
Governance	Transparency Level	2015 MPF	2016 MPF

Source: CIH, Hospital Information Communication; CNES, National Register of Health Establishments; DATASUS, Department of Informatics of the Unified Health System; e-manager-AB, PHC system; HDI, Human Development Index; IBGE, The Brazilian Institute of Geography and Statistics; MDS, Social Development Ministry; MPF, Federal Public Ministry; PNI, National Immunization Program; PNS, National Health Survey; SIA, Ambulatory Information System; SIH, Hospital Information System; SIAB; SISAB, Primary Care Health Information System; SIM, Mortality Information System; SINAN, Notifiable Diseases Information System; SINASC, Information System on Live Births; SIOPS, Information System for the Public Budgets in Health; SISCOLO and SISMAMA, Cervical and breast cancer screening information systems; STN/MF, National Treasury Secretariat/ Ministry of Finance; UNDP, United Nations Development Program.

**Figure 1:**
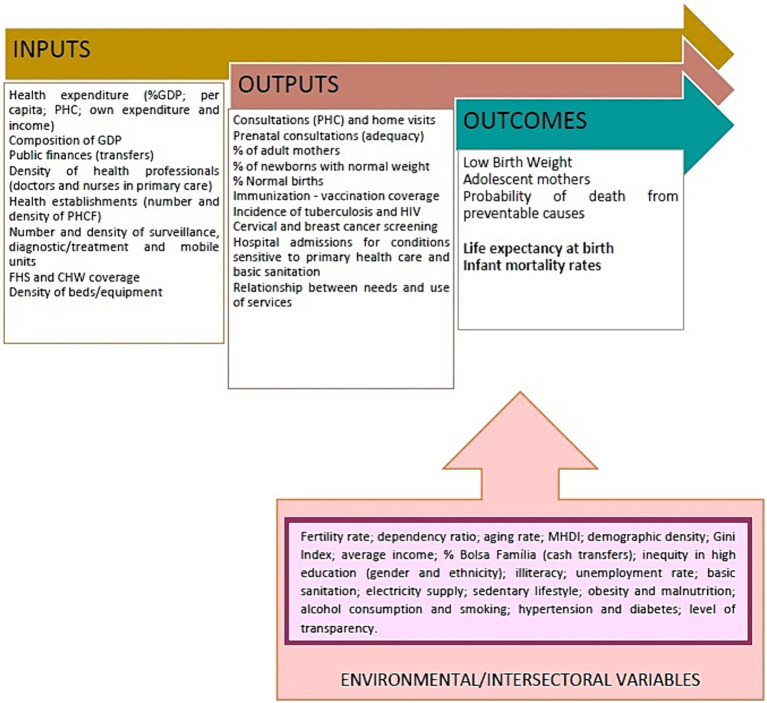
Health production model with the following variables: inputs or resources, products or intermediate results (outputs), final results (outcomes), and environmental results (throughput or control).

We also present Figure 1 to make it easier to understand the analysis of the health production process, with the main variables analyzed according to the stages, from inputs/resources through health outputs to final results (outcomes), cross-cut by intersectoral variables.

Considering the few efficiency analyses at the local level, we carried out local efficiency analyses in remote rural municipalities, simultaneously comparing them to the Federative Units (FU) (states) to which they belong. At that level, it is possible to evaluate the use of health services according to health needs, which allows for overcoming problems already perceived in the local analyses. Compared to the global efficiency analyses, local analyses usually present lower determination coefficients in the health dimension (32).

We focused on equity in the financing and distribution of health teams, which involves allocating human and financial resources according to health needs and the proportion of the state’s GDP devoted to health. We evaluated the following variables: sources of funding, local expenditures, and revenues for health, the proportion of allocation of resources to primary care of total health spending, staffing and funding arrangements, and transfers between different levels of government.

Regarding equity in access, we assessed the following aspects: the use of health services according to needs; financial and non-financial barriers (access, use, coverage, and prevention); the scope of actions, services, and practices, in addition to hospitalizations from PHC and basic sanitation sensitive causes.

Environmental or intersectoral variables include demographic, socioeconomic, governance, and risk factors (environmental and chronic diseases) dimensions.

### 2.1 Statistical analysis

We used two complementary techniques: Data Envelopment Analysis allows analysis throughout the stages of the production process (33), while the Fixed Effects model enables a dual analysis (34), combining effectiveness (results of the coefficients obtained) with efficiency (residual analysis). The number required for the sample is at least 10 observations per independent variable for the regression (FE), or *N* > 104 + *m*, where “*m*” is the number of independent variables (35). Considering that the set has approximately 300 remote rural localities (RRL) per period and we did not analyze more than twenty variables simultaneously, we have complied with the proposed rules. In the case of DEA, the rule proposed by Cooper et al. (36) is that the sample exceeds the number of inputs (*m*) and outputs (*s*) by several times, specifically that “*n*” is greater than max. [m*s, 3* (*m* + *s*)]. For more details, please consult Supplementary material S1.

**Figure 2:**
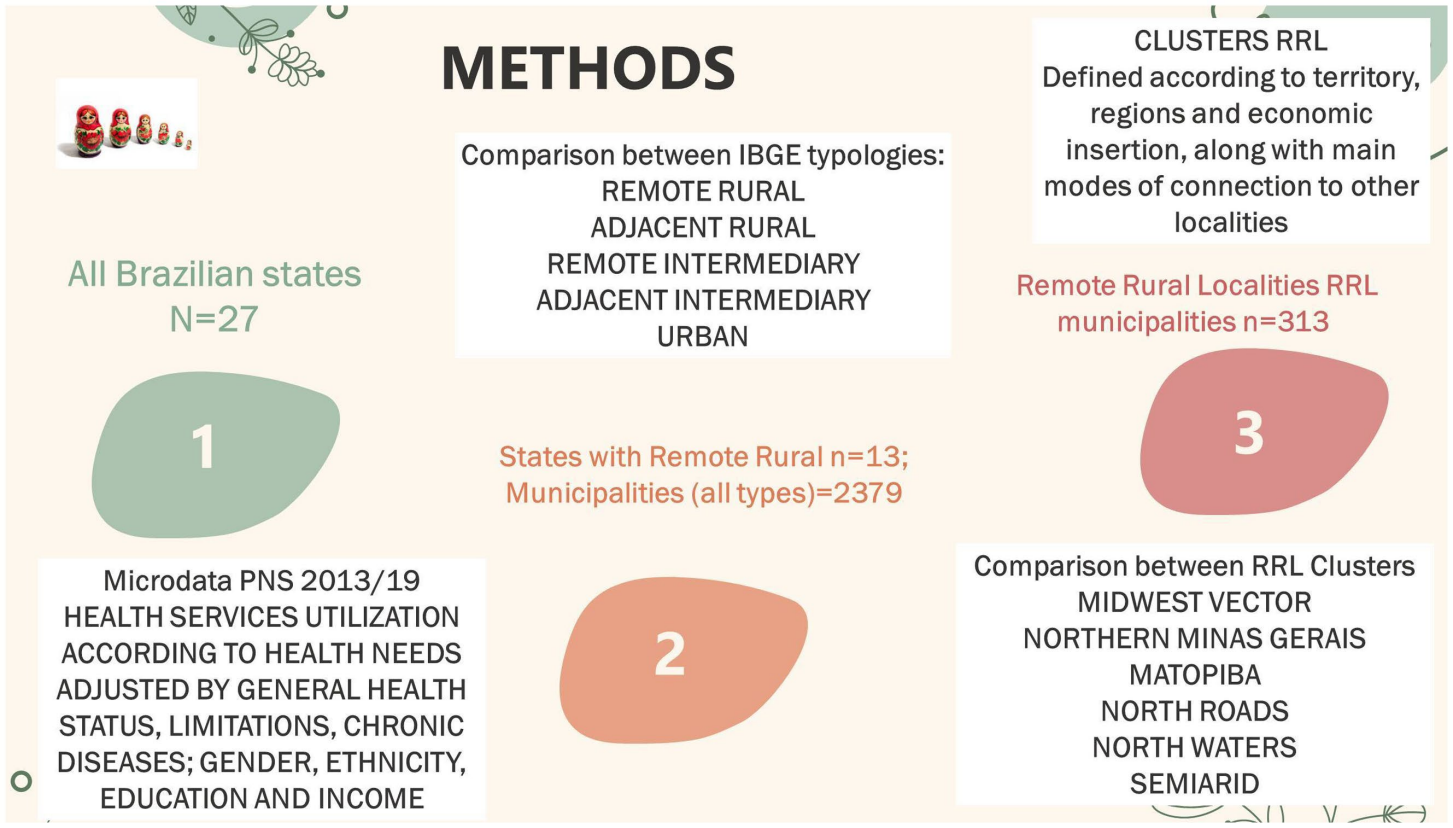
The three steps in our methods.

We performed three analyses to deepen our understanding of the remote rural localities. We have prepared a step-by-step infographic to help readers understand the immersion in the remote rural reality (Figure 2).

Our hypothesis is that RLL might have higher efficiency and effectiveness in health, considering the limited resources and their capacity to mobilize health resources, especially concerning the healthcare FHS models. We also knew of the heterogeneity of these regions and clusters of the RRL. Hence, we supposed that the collective PHC models would be stronger in distant regions, where there are mobile health facilities and customized riverine health teams.

We have tested our hypothesis in three layers of statistical analysis, in order to deepen the RRL reality. First, we studied all of Brazil’s states, comparing states with and without these localities, through fixed effects models, globally and dimensionally (resources, health and intersectoral variables). The use of health services according to health needs was a very important test, since we had past experiences that the health dimension yielded low determination coefficients.

Then, we examined the RRL states and performed DEA and fixed effects, comparing the urban–rural typology. Finally, we compared different sociogeographical clusters within the RRL.

### 2.2 Empirical strategy

Our empirical strategy comprises the use of logistic regression in order to assess the utilization of services according to health needs, the fixed effects model and data envelopment analysis.

Fists, we explored health and sociodemographic variables to measure service utilization with a logistic regression model in all Brazilian states. The advantages of performing logistic regression is the small number of requirements, such as not presenting multicolinearity nor heterocedasticity, along with high reliability. Maximum likelihood is used to estimate the parameters., presented either by its linear form, *logit* (*P*) or the probability odds log (*p*/(1 − p)), with *P* exhibiting the formulae:


$$P\left(y=1\right)=\frac{1}{1+e^{-f\left(x\right)}}$$

where *f*(*x*) = *β*_0_ + *β_e_ X_e_* + *β*_*c*1_ *X*_*c*1_ + *β*_*ec*1_ *X_e_* * *X*_*c*1_ … + *β_cn_ X_cn_*

*β*_0_ corresponds to the intercept; *β_e_ X_e_* are the equation terms, *β* is the coefficient and *X* refers to the independent exposure variables; *β*_*c*1_ *X*_*c*1_ is the covariable 1 (until *n*, *β_cn_ X_cn_*); *β*_*ec*1_ *X_e_* * *X*_*c*1_ is the interaction term between the exposure variables and covariable 1 (confounding variable).

The sample size is about 10 to 30 observations per variable. We applied the *stepwise backward* method, from the initial complete model, until managing to find the adequate final model. The analyses were carried out using *Stata SE 14.0* software.

We then proceeded to use the fixed effects regression model for panel data (time-invariant characteristics) including confounding and interaction variables in the effectiveness analysis, which considers the regression’s beta coefficients, whilst the location-specific effect was calculated by adding the fixed effect to the output residuals, which calculates efficiency. We employed this method to all three stages listed below. The analyses were carried out using *Stata SE 14.0* software.

The equation below was used to obtain the final models:


$$\begin{array}{l} Y_{it}=\beta_{0}+\beta_{1}\;X_{\left(1,it\right)}+\cdots +\beta_{k}\;X_{\left(k,it\right)}\\ +y_{2}\;E_{2}+\cdots +y_{n}\;E_{n}+\delta_{2}\;T_{2}+\delta_{t}\;T_{t}+\mu_{it} \end{array}$$

where *Y_it_* is the dependent variable, e.g., Life expectancy at birth and Infant Mortality rates (DV where *i* = unit and *t* = time); *X*_(*k,it*)_ represents the independent variables (VI), *β_k_* is the coefficient for the VIs, *u_it_ i* is the error term, *ɛ_n_* is the unit *n*. *γ*_2_ is the coefficient for the units. *T_t_* is the time, and *δ_t_* is the coefficient relative to time. We have employed this method alone to compare Brazilian states (with and without RRL) and the typologies within RRL only and to compare urban-rural typologies along with DEA (in the states with RRL).

The fixed effects model has a relatively good correlation to the DEA analysis. All the same, we employed DEA dynamic model with stages to deepen the analysis of the states with and without RRL, with different dependent variables, to ensure validity for the models. Besides life expectancy at birth and infant mortality rates, we also had models for the probability of deaths due to preventable causes, the proportion of newborns with low birth weight, and the proportion of adolescent mothers.

The equation for the network dynamic model is derived from the slack model, product-oriented with k steps:


$$\frac{1}{\tau_{0}^{*}}=\max\;\Sigma_{k=1}^{K}W^{k}\left[\left[1+\frac{1}{r^{k}+\sum_{h\in F_{k}}t_{k,\;\;h}}\left(\frac{\sum_{kr=1}^{r}s_{r0}^{k+}}{y_{r0}^{k}}+\frac{\Sigma_{h\in F_{k}s_{h0}^{\left(k,h\right)+}}}{z_{h0}^{\left(k,h\right)}}\right)\right]\right]$$

where, *w_k_* is the relative weight of each division; *F_k_* is the set of stages with links (*k*, *h*); ∑*K_k_* = 1 *w_k_* = 1; *w_k_* ≥ 0; $s_{k}^{+}$ are the output slack vectors; *r_k_* is the number of outputs in stage *k*; *t*_(*k*, *h*)_ is the number of products in the link between stage *k* and *h*; *s*_*h*0_^(*k*, *h*)+^ are the slack vectors of the links and *z* deals with the intermediate products. We performed these analyses with the *Max Dea 8 Ultra* (DEA software).

### 2.3 Data collection

All our data were secondary, aggregated and public databases, used in an ecological perspective, except for the use of health services according to health needs, which was extracted from the National Health Survey.

The National Health Survey was carried out by the Ministry of Health, together with the Brazilian Institute of Geography and Statistics. The survey was carried out with a probabilistic sample of households, with primary sampling units, composed by at least one census sector and 12–18 households, according to the number of households in every state. In each household, a resident (15 years or older) was randomly selected and interviewed on behalf of the group living together, with questions about lifestyle, work, chronic diseases and violence. The method employed at all stages was the simple random sample. Data were collected during 2019–2020 from 108,457 households.

### 2.4 The three analyses carried out

#### 2.4.1 All Brazilian states

First, an analysis of efficiency and effectiveness was carried out with all Brazilian Federative Units (26 states and 1 Federal District), including resource, health, and intersectoral categories, using the fixed effects model. We adopted the health production model, with life expectancy at birth (LEB) and infant mortality rates (IM) as effect variables, in two stages. It is noteworthy that the models relating to the results of health services relate to the use of services according to health needs. We included microdata from the 2013 and 2019 National Health Survey-PNS in the database to circumvent the bias of using ecological variables (37). We compared the states with and without RRL as the first level of analysis.

We calculated the use of health services at the state level, according to health needs, by regressions in which we tested the use of services, general health status, and limitations resulting from chronic diseases, adjusting for gender, race, marital status, education, and income. The variables of age, chronic diseases (collinear), and enrollment in FHS units were not statistically significant.

#### 2.4.2 Brazilian states with remote rural localities (municipality level)

Then, we carried out efficiency and effectiveness analyses using the EF and DEA models, comparing the remote rural typology with the others (2). We performed a controlled comparison among the states with RRL between the remote rural typology and the typologies of rural adjacent, intermediate adjacent and remote, and urban. For further details, please check Supplementary material S2.

This analysis considered only the Brazilian states with RRL (*n* = 13; 2,379 municipalities). The states with RRL comprise all seven states in the north region, three states in the northeast region (Bahia, Maranhão, and Piauí), and two in the Midwest region (Mato Grosso do Sul and Mato Grosso), in addition to Minas Gerais, the only one in the southeast region. Three were excluded, due to low representativeness of their units (2 in the South and 1 in the Northeast).

The dependent variables in these models were, in addition to life expectancy at birth and infant mortality, the probability of deaths due to preventable causes, the proportion of newborns with low birth weight, and the proportion of adolescent mothers. In this way, we decided to evaluate the intermediate health results (outputs), which could relate in a more detailed and proximal way to the different organizational arrangements present in these locations, and the results of efficiency and effectiveness in health outcomes.

#### 2.4.3 Remote rural localities (municipalities) only

In addition, we also studied the typologies of the different RRL among themselves. This typology was elaborated by Bousquat et al. (4), starting from the category of analysis of the territory use and having as reference the study of Santos and Silveira (38) and the identification of four regions, the concentrated one (South and Southeast) the one of Recent Peripheral Occupation; the Northeast and the Amazon. Bousquat et al. (4) then classified the RRL by the respective logic of the economic circuit insertion and their prevalent form of interconnection with the other points of the territories, whether by land or river.

Next, Bousquat et al. (4) scanned the RRL and broke them down by variables that denote the rarefaction and remoteness of the population, in addition to economic capacity. The qualitative analysis led to the design of six clusters (313 RRL or 97% of the total) namely: Matopiba (*n* = 92); Northern Minas Gerais (NMG) (*n* = 22); Midwest Vector (MWV) (*n* = 84); Semiarid (*n* = 42); North Waters (*n* = 45); and North Roads (*n* = 28). For more information, please refer to Supplementary material S3.

Finally, we compared the different RRL clusters with each other (4), with regard to intersectionality. Thus, we compared the Midwest Vector, Matopiba, Northern Minas Gerais, North Roads, North Waters, and Semiarid concerning the reach of efficiency and effectiveness in health, with equity as a reference to these relationships.

We compared the typologies of the municipalities and the RRL clusters using the Kruskal–Wallis test and the multiple comparisons with the Dunn test, and Bonferroni adjustments.

We performed the analyses with *Stata SE 10.1 and 14.0* software (Fixed Effects model) and *Max Dea 8 Ultra* (DEA).

## 3 Results

**Figure 3:**
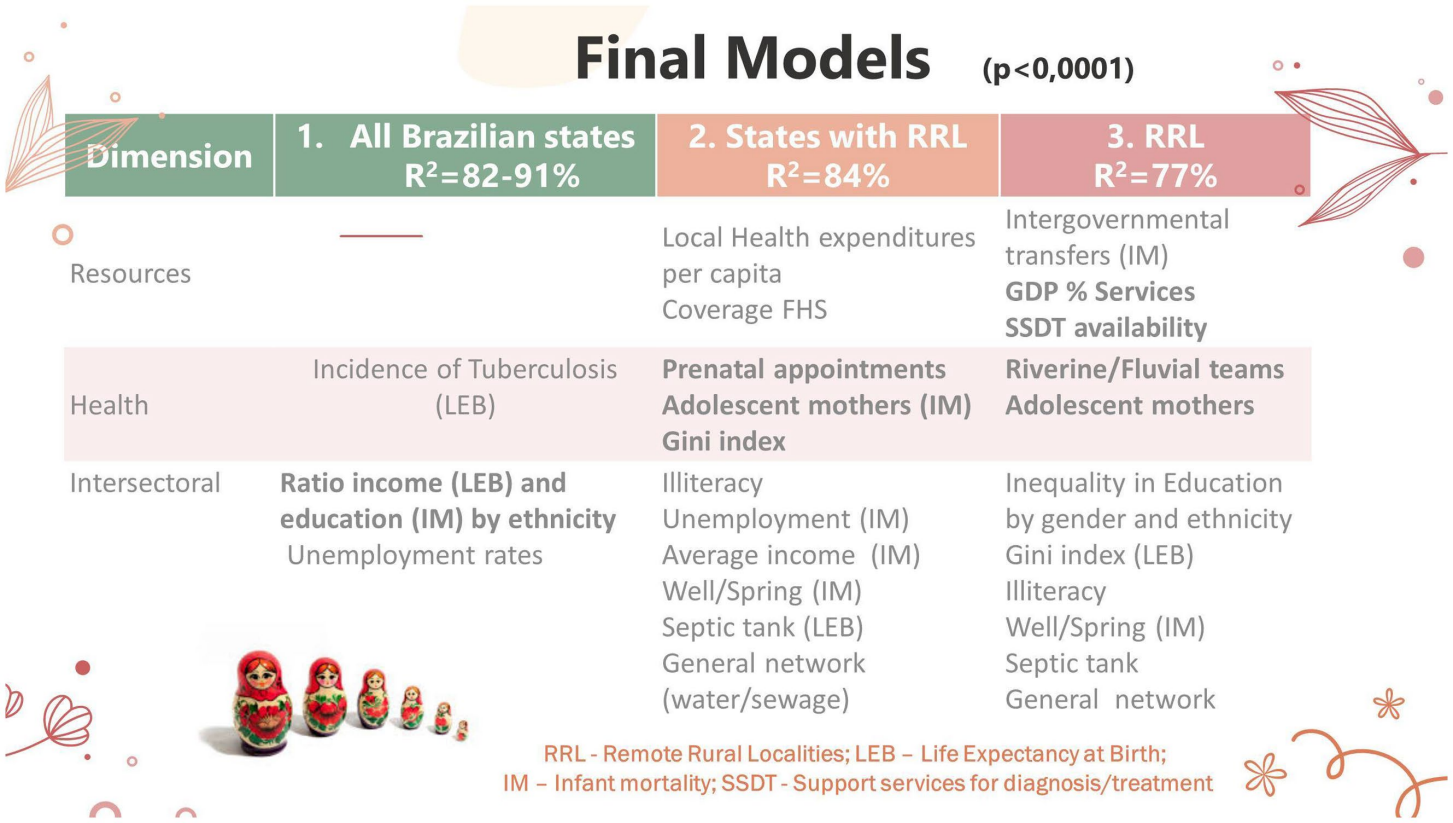
Summary results of the three analyses for life expectancy at birth and infant mortality.

We present the results in three levels of analysis, which allowed us to deepen our understanding of the RRL and their health needs relating to their territories and contiguities. The main summary results are available in Figure 3. Next, we give more details on each analysis result.

### 3.1 Comparison between Brazilian states with and without RRL, considering health needs

First, we performed bivariate analyses on the dimensions according to the fixed effects model. We retrieved data on service utilization from the 2013 and 2019 National Health Surveys (PNS) (37) based on the relationship between health needs and the use of services (6, 7). We selected the fixed effects model because of its ability to capture health effectiveness through its coefficients and efficiency through the analysis of residuals. The data on the use of services was essential to reduce the gap between needs and the utilization of services, with less aggregation of health data, which usually presents very low determination coefficients.

The highest use of services occurred among those with limited functional capacity to carry out activities of daily living, with worse general health status, female, white, married, and with a better level of education and higher income (Table 2). The variables age, chronic diseases, and registration with the FHS did not remain in the final model.

**Table 2:** Regression of use of health services according to selected sociodemographic and health variables.

Variable	OR (IC 95%)	*p*
Limited functional capacity (LFC)—ref (yes)	0.63 (0.59–0.66)	<0.001
General health status ref (better)	1.46 (1.41–1.51)	<0.001
Sex (ref: male)	1.42 (1.35–1.48)	<0.001
Race (ref: white)	0.98 (0.96–0.99)	0.019
Marital status (ref: married)	0.96 (0.94–0.98)	<0.0001
Education level (ref: lower)	1.07 (1.05–1.09)	<0.001
Income level (ref: lower)	1.11 (1.09–1.14)	<0.001
Constant	0.12 (0.10–0.15)	<0.001

*N* = 77.887; Population 85,801,346; *F* = 207.12; *p* < 0.0001.

Based on the bivariate analysis (Supplementary material S4), we selected the variables that would be part of the multivariate analysis according to their statistical significance and coefficient value concerning other variables in their dimension and group.

The results of the comparison of Brazilian states with and without the RRL (Table 3) revealed high determination coefficients (*R*^2^ = 82–96%), drawing attention, especially to the health dimension, which had high values both in the efficiency and productivity analysis, i.e., the relationship between resources and services (61–93%), very similar to the other dimensions (Table 4). These results were even more significant for service utilization, ranging from 77–82%. Moreover, in the final model for life expectancy at birth, the variable incidence of tuberculosis remained a relevant variable.

**Table 3:** Final regression models for selected effect variables, according to dimensions and general models (model with all states: Brazil, 2010 and 2019).

Model/variable	Life expectancy at birth—LEB variable (beta)	Infant mortality—IM variable (beta)
Physical and financial resources	% population receiving < ¼ MW (−0.40) Health expenditure % GDP (−1.33) Public administration % GDP (6.74 × 10^−5^) Constant (87.00) (*R*^2^ = 96%; *p* < 0.0001)	% population receiving < ¼ MW (1.38) Density of nurses (−3.24) Public administration % GDP (−1.55 × 10^−7^) Constant = −14.83 (*R*^2^ = 96%; *p* < 0.0001)
Health production	Use of health services according to health needs (0.41) TB incidence (−0.10) Smoking (−0.36) General proportion of normal deliveries (−0.29) Constant (88.14) (*R*^2^ = 95%; *p* < 0.0001)	Use of health services according to health needs (−1.94) Hospitalizations sensitive to basic sanitation (2.49) Smoking (2.68) Constant (8.34) (*R*^2^ = 86%; *p* < 0.0001)
Intersectoriality	Ratio ethnicity income (−5.60) Ratio ethnicity education (−1.93) Unemployment rate (−0.40) Inadequate sanitation (−0.22) Constant (94.44) (*R*^2^ = 92%; *p* < 0.0001)	Gini index (122.20) Ratio ethnicity education (11.42) Unemployment rate (0.96) Constant (−93.97) (*R*^2^ = 85%; *p* < 0.0001)
General—all dimensions	Ratio income ethnicity (−7.91) Unemployment rate (−0.44) TB incidence (−0.15) Constant (96.96) (*R*^2^ = 91%; *p* < 0.0001)	Ratio ethnicity education (12.14) Unemployment rate (1.53) Constant (26.94) (*R*^2^ = 82%; *p* < 0.0001)

**Table 4:** Final regression models for health variables, according to the dimensions of resources and environmental variables (model with all states: Brazil, 2010 and 2019).

Model/variable	Physical/financial resources variable (beta)	Intersectoriality variable (beta)
Service utilization	% population that receives < ¼ MW (−0.14) Public administration % GDP (6.23 × 10^−8^) Constant (18.10) (*R*^2^ = 77%; *p* < 0.0001)	Unemployment rate (−0.46) Constant (20.16) (*R*^2^ = 82%; *p* < 0.0001)
Incidence tuberculosis TB	% population that receives < ¼ MW (−0.14) Health expenditure % GDP (6.46) Public administration % GDP (−1.91 × 10^−7^) Constant (−6.83) (*R*^2^ = 61%; *p* < 0.0001)	Ratio ethnicity education (7.01) Unemployment rate (0.99) Constant (9.27) (*R*^2^ = 59%; *p* < 0.0001)
Hospitalizations BSSC^*^	% population receiving < ¼ MW (0.17) Health expenditure % GDP (1.03) Constant (−5.44) (*R*^2^ = 81%; *p* < 0.0001)	Ratio ethnicity education (1.22) Inadequate sanitation (0.20) Constant (−2.10) (*R*^2^ = 70%; *p* < 0.0001)
Normal deliveries (%)	% population receiving < ¼ MW (0.65) Constant (32.60) (*R*^2^ = 93%; *p* < 0.0001)	Gini index (124.62) Ratio ethnicity education (4.51) Inadequate sanitation (0.31) Constant (−42.00) (*R*^2^ = 90%; *p* < 0.0001)
Smoking (%)	% population receiving < ¼ MW (0.17) Constant (8.41) (*R*^2^ = 72%; *p* < 0.0001)	Gini index (32.06) Ratio ethnicity education (1.73) Constant (−11.21) (*R*^2^ = 63%; *p* < 0.0001)

^*^MW, minimum wage; BSSC, basic sanitation-sensitive causes.

Most strikingly, the final models for both effects retained variables relating to ethnic inequity in income (LEB) or education (IM) in conjunction with unemployment rates. Thus, the final models are more intersectoral, with no resource dimension variables remaining.

Regarding life expectancy at birth and infant mortality, the best results are in the southeast, part of the south and the capital, besides a small part of the Amazon region (LEB only). Considering the dimensions, the resources and health dimensions present better results for the North and Northeast regions, whereas the intersectoral dimension shows higher scores for the southeastern region and the capital, besides a small part of the Amazon region (LEB). To find out more details, see Supplementary material S5.

It is worth mentioning that the level of transparency [Federal Public Ministry (MPF)] was highly significant in the bivariate analysis in the expected direction, although it did not remain in the dimensional models. In contrast, income inequality by gender was statistically significant in the opposite direction and did not remain in the final models.

We also present graphs showing the potential gains in years of life or the reduction in infant mortality rates attributed to efficiency gains by state and macro-region for the set and the dimensions analyzed (Supplementary material S5).

**Graph 1:**
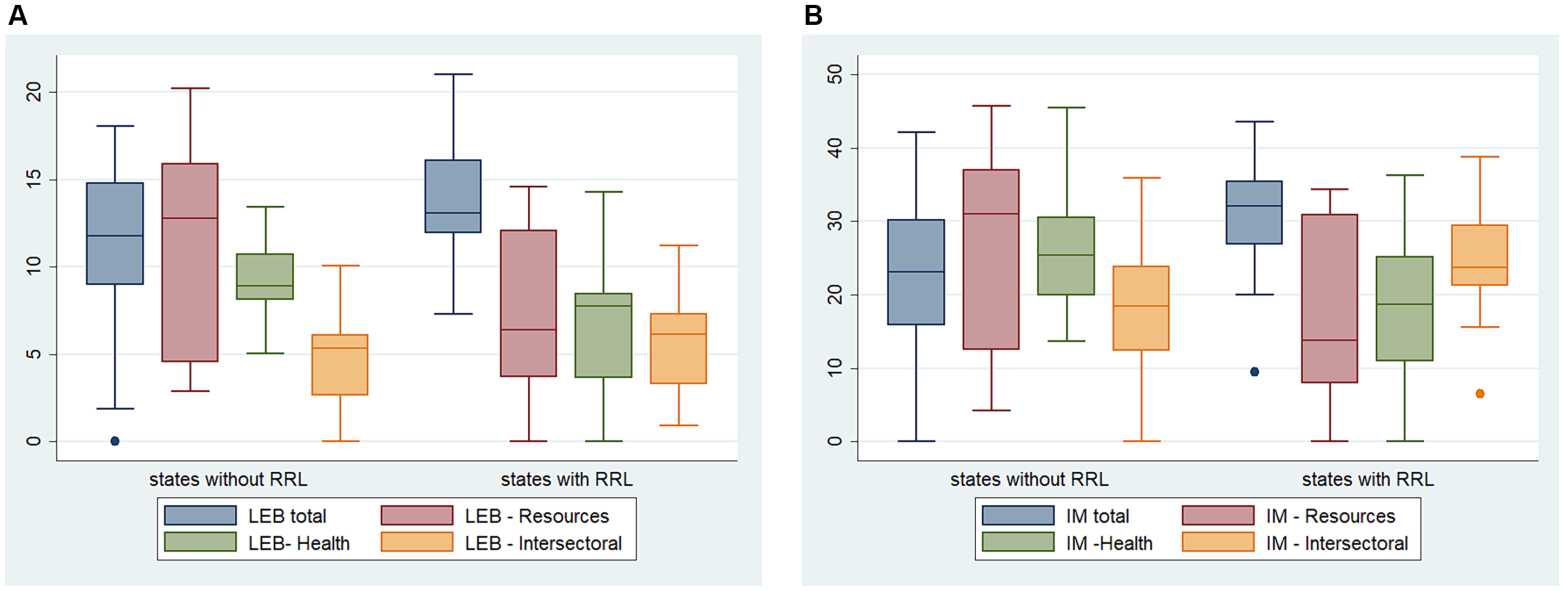
**(A)** Distribution of potential years of life gained attributed to efficiency gains according to states with and without remote rural municipalities. **(B)** Distribution of the potential reduction in infant mortality due to improved efficiency according to states with and without remote rural municipalities.

The comparison between the 13 states with and without RRL (*n* = 14) on Graphs 1A,B shows that at the level of resources and health, there is greater efficiency in states with remote rural municipalities (*p* ≤ 0.01), while regarding the intersectoral and global models, the reverse is perceived, being significant for infant mortality (*p* ≤ 0.01).

We also performed a technical efficiency analysis (productivity), associating the physical and financial resource variables with the health dimension variables, as well as the intersectoral variables. The coefficients of determination were very high (Table 4), ranging from 61–93% for the resource dimension and 59–90% for the intersectoral dimension. The lowest values were for the tuberculosis incidence variable, whereas the highest was for the proportion of vaginal births. The most relevant variables were health spending as a % of GDP, the % of the population earning less than ¼ of the minimum wage, and the % of GDP allocated to Public Administration. Concerning the intersectoral variables, the Gini index, ethnicity inequality of higher education, the unemployment rate, and inadequate sanitation stand out.

Regarding the health variables (Table 4), we depict that the use of health services was negatively associated with low income and positively associated with the proportion of GDP of the Public Administration and employment rates. Thereby, the states containing RRL presented lower utilization than the others. It is worth mentioning that the incidence of tuberculosis, hospitalizations for sanitation-sensitive causes, vaginal birth deliveries, and smoking were influenced by ethnicity inequalities in education and the proportion of the population with low income, according to variables remaining in the dimensional models. Furthermore, health expenditure as a % of state GDP was relevant for the incidence of tuberculosis (along with unemployment and lower proportions of GDP derived from the Public Administration) and for hospitalizations due to sanitation-sensitive causes. Income inequalities (Gini index) were relevant for the higher proportion of vaginal birth deliveries and smoking prevalence.

### 3.2 Comparison of efficiency among the states with RRL, between the remote rural typology and the urban, adjacent or remote intermediate, and adjacent rural

In the second analysis, which involves only the states with RRL, we combined the fixed effects model with network and dynamic DEA. This process yielded satisfactory correlations of the efficiency scores from both models, higher for the indicators closest to health services (intermediate results), such as the deaths from preventable causes, the % of low birth weight, and the % of teenage mothers. Once again, we used bivariate analyses to select the variables considered for the multivariate models by dimension and group of variables (Supplementary material S6).

The models regarding health levels (LEB and IM) with only the 13 states containing RRL remained with the Gini index, local health expenditure *per capita*, and FHS coverage, in addition to basic sanitation (general network, septic tank, and well/spring), illiteracy and unemployment (IM). Additionally, the variables related to adequate prenatal care and the proportion of teenage mothers (IM) stand out. Remarkably, the determination coefficients were 84%, presenting a regular correlation between the methods of FE and DEA of 46% (Tables 5, 6).

**Table 5:** Position in relation to the average rank of efficiency for each geographic classification (model with 13 states containing remote rural localities).

Geographic classification^*^	LEB	IM	Preventable deaths (%)	Low birth-weight (%)	Teen mothers (%)	Average rank
Remote rural (RR)	2.2 (1)	2.0 (1)	1.3 (1)	2.8 (1.5)	2.6 (2)	2.2 (1)
Urban (U)	4.6 (5)	4.8 (5)	4.2 (5)	3.2 (4.5)	3.3 (3)	4.0 (5)
Adjacent rural (AR)	2.7 (3)	2.5 (2)	2.8 (2)	3.2 (4.5)	2.0 (1)	2.6 (2)
Remote intermediate (RI)	2.6 (2)	2.8 (3)	3.1 (3)	2.8 (1.5)	3.7 (5)	3.0 (3)
Adjacent intermediate (AI)	2.8 (4)	3.0 (4)	3.7 (4)	3.1 (3)	3.5 (4)	3.2 (4)
Remaining variables in the final model	*n* = 7	*n* = 9	*n* = 6	*n* = 3	*n* = 5	*n* = 19
FHS coverage	⨯					1
FHS % teams			⨯			1
CHW			⨯			1
FHS home visits				⨯		1
% PHC physicians				⨯	⨯	2
Gini	⨯	⨯			⨯	3
% Illiteracy	⨯	⨯				2
% High education					⨯	1
% Unemployment		⨯	⨯			2
Average income		⨯			⨯	2
Diarrhea/ARI < 2y			⨯			1
Prenatal consultations	⨯	⨯	⨯			3
Low birthweight			⨯			1
% Adolescent mothers		⨯				1
Septic tank	⨯					1
Well/spring		⨯				1
General network	⨯	⨯				2
Local health expenses	⨯	⨯		⨯		3
Intragov transfers					⨯	1
*R* ^2^	84%	84%	10%	2%	38%	*p* < 0.0001
Correlation FE and DEA	47%	46%	96%	97%	87%	*p* < 0.0001

Multiple comparisons—Dunn test *p* = 0.0001; LEB AI/AR > RI/RR; U > all; IM AI > RI/AR/RR; AR > RI/RR. U > other; preventable deaths AI > RI/AR > RR. U > all; low birthweight AI > RI/RR; AR > RR. U > all; adolescent mothers IR > all; RR > AI > AR; U > AR; RR > U. ARI, acute respiratory infection; CHW, Community Health Workers; EPC, local health expenses per capita; FHS, Family Health Strategy; IM, infant mortality; PN, prenatal; LEB, life expectancy at birth.

**Table 6:** Final regression models for selected effect variables, according to dimensions and general models (model with remote rural localities states only: Brazil, 2010 and 2019).

Effect variable	Independent variables (beta)	Details
Life expectancy at birth	Local health expenses *per capita* (3.9 × 10^−3^)	Constant (67.48)
	Gini index (−4.24)	(*n* = 4.678; *R*^2^ = 84%; *p* < 0.0001)
	FHS coverage (0.52)	
	Illiteracy (−0.25)	
	Prenatal appointments (5.36)	
	Septic tank (0.01)	
	General network (0.05)	
Infant mortality	Local health expenses *per capita* (−0.01)	Constant (38.58)
	Gini index (−12.36)	(*n* = 4.724; *R*^2^ = 84%; *p* < 0.0001)
	FHS coverage (−1.69)	
	Illiteracy (0.67)	
	Prenatal appointments (−16.06)	
	Adolescent mothers (5.28)	
	Unemployment rates (0.08)	
	Average income (−0.02)	
	Well/spring (−0.07)	
	General Network (−0.16)	
Preventable deaths	Proportion of FHS teams and oral health (−0.40)	Constant (2.28)
	Proportion of CHW teams (−1.00)	(*n* = 3.379; *R*^2^ = 10%; *p* < 0.0001)
	Prenatal appointments (0.73)	
	ARI/diarrhea <2a (−0.52)	
	Low birthweight (2.09)	
	Unemployment (−0.01)	
Low birthweight	Local health expenses *per capita* (7.35× 10^−6^)	Constant (0.10)
	Domiciliary visits (−0.01)	(*n* = 3.800; *R*^2^ = 2%; *p* < 0.0001)
	Proportion of medical doctors PHC (−0.02)	
Adolescent mothers	Intergovernmental transfers (−5.18 × 10^−4^)	Constant (0.32)
	Higher education (−2.08 × 10^−3^)	(*n* = 4.605; *R*^2^ = 38%; *p* < 0.0001)
	Gini index (0.07)	
	Average income (−7.29 × 10^−5^)	
	Density of medical doctors PHC (−12.78)	

The family health and oral health teams and the CHW showed relevant effectiveness results in reducing *preventable deaths*, combined with employment, adequate prenatal care, adequate birth weight, and treatment for acute respiratory infections and diarrhea in children under 2 years of age.

Regarding *low birth weight*, the following variables remained relevant: home visits by the Family Health Team, *per capita* health expenditure, and the proportion of primary care physicians.

Concerning the proportion of *teenage mothers*, intergovernmental transfers are a fundamental resource variable, as well as the density of primary care physicians, high education, average income, and income inequality (Gini index).

The determination coefficients were much lower than in the health levels models precisely because they represent intermediate output variables (2–38%). On the other hand, they showed a high correlation between the methods employed (FE and DEA), ranging from 87–97% (Tables 5, 6).

It is possible to see the distribution of these results (Supplementary material S7) for LEB, IM, deaths from preventable causes, % of low birth weight, and adolescent mothers, by typology and by state, with the potential gains in years of life or the reduction in the other measures (rates and proportions) due to improved efficiency.

The RRL stand out compared to the other typologies, especially regarding avoidable deaths, LEB, and IM (Table 5). On another note, the RRL exhibited a tie with the remote intermediate municipalities and a second-place position after the adjacent rural municipalities for low birth weight and the proportion of adolescent mothers. We may then show a notable gradient among typologies for the set of models: the RRL with the best values, followed by the adjacent rural, remote intermediate, adjacent intermediate, and, lastly, urban. To check the Brazilian states ranking, please refer to Supplementary material S7.

Remote localities (intermediate and rural) have higher distributions of overall income inequality (Gini index) and income inequality by gender relative to adjacent municipalities (intermediate and rural). On the other hand, the differences in education levels by gender and ethnicity are lower in remote rural municipalities than in the remaining typologies and represent a determining factor for their efficiency and effectiveness.

### 3.3 Comparison between remote rural clusters regarding efficiency and effectiveness, with exclusive models intersectionality-wise

In the third analysis, we evaluated the RRL clusters only with the multivariate fixed effects analysis since we had already assessed the regression models and knew which relevant variables to test the final models.

It is interesting to compare the different models, as if in layers, according to the weight of the RRL (Table 7). When we analyze all the Brazilian states, we realize that the final model encompasses intersectional and health issues concerning the incidence of tuberculosis. When we repeat the model with only the 13 states containing RRL, we verify emerging variables regarding income inequality (Gini index), resources (health expenditure *per capita*) and FHS coverage, basic sanitation specific to these localities (general network, septic tank, and well/spring), illiteracy and unemployment (IM). The variables related to adequate prenatal care and the proportion of teenage mothers (IM) draw attention.

**Table 7:** Comparison between the different models, according to data aggregation.

Model/Variable	Life expectancy at birth variable (beta)	Infant mortality variable (beta)
All Brazilian states	Income ethnicity ratio (−7.91) Unemployment rate (−0.44) Tuberculosis incidence (−0.15) Constant (96.96) (*n* = 54; *R*^2^ = 91%; *p* < 0.0001)	Education ethnicity ratio (12.14) Unemployment rate (1.53) Constant (−26.94) (*n* = 54; *R*^2^ = 82%; *p* < 0.0001)
RRL states only	Local health expenses *per capita* (3.9 × 10^−3^) Gini index (−4.24) FHS Coverage (0.52) Illiteracy (−0.25) Prenatal appointments (5.36) Septic tank (0.01) General Network (0.05) Constant (67.48) (*n* = 4.678; *R*^2^ = 84%; *p* < 0.0001)	Local health expenses *per capita* (−0.01) Gini index (−12.36) FHS coverage (−1.69) Illiteracy (0.67) Prenatal appointments (−16.06) Adolescent Mothers (5.28) Unemployment rates (0.08) Average income (−0.02) Well/spring (−0.07) General Network (−0.16) Constant (38.58) (*n* = 4.724; *R*^2^ = 84%; *p* < 0.0001)
RRL only	Gini index (−4.39) GDP %-services (15.35) Riverine/fluvial teams (5.23) Inequality in education by gender/ethnicity (−1.06) Adolescent mothers (−9.73) Illiteracy (−0.29) Septic tank (0.03) General network (0.11) SSDT availability (4.69) Constant (72.78) (*n* = 610; *R*^2^ = 77%; *p* < 0.0001)	Intragovernmental transfers (−0.13) GDP % services (−48.07) Riverine/fluvial teams (−34.83) Inequality in education by gender/ethnicity (3.16) Adolescent mothers (20.74) Illiteracy (0.74) Well/spring (−0.13) Septic tank (−0.09) General network (−0.31) SSDT availability (−16.94) Constant (46.42) (*n* = 608; *R*^2^ = 77%; *p* < 0.0001)

Brazilian states, states with remote rural localities (RRL and only Brazil, 2010 and 2019). ^*^FHS, Family Health Strategy; SSDT, Support services for diagnosis and treatment.

When we analyze the RRL only (Table 7), the models denote the importance of riverine and fluvial health teams, the proportion of diagnostic and treatment units, very relevant in these localities, in addition to the proportion of illiteracy and teenage mothers, even in the LEB model. This situation, of the level of education, also manifests itself in the inequity of reaching high levels of schooling among gender and ethnicity (higher inequities observed in the clusters of the Midwest Vector and North Waters). The proportion of the GDP derived from services is another relevant variable, which marks the living standards since they constitute advances allied to the agricultural activity in these localities. All models showed high determination coefficients (77–81%), especially in the models with more states.

**Graph 2:**
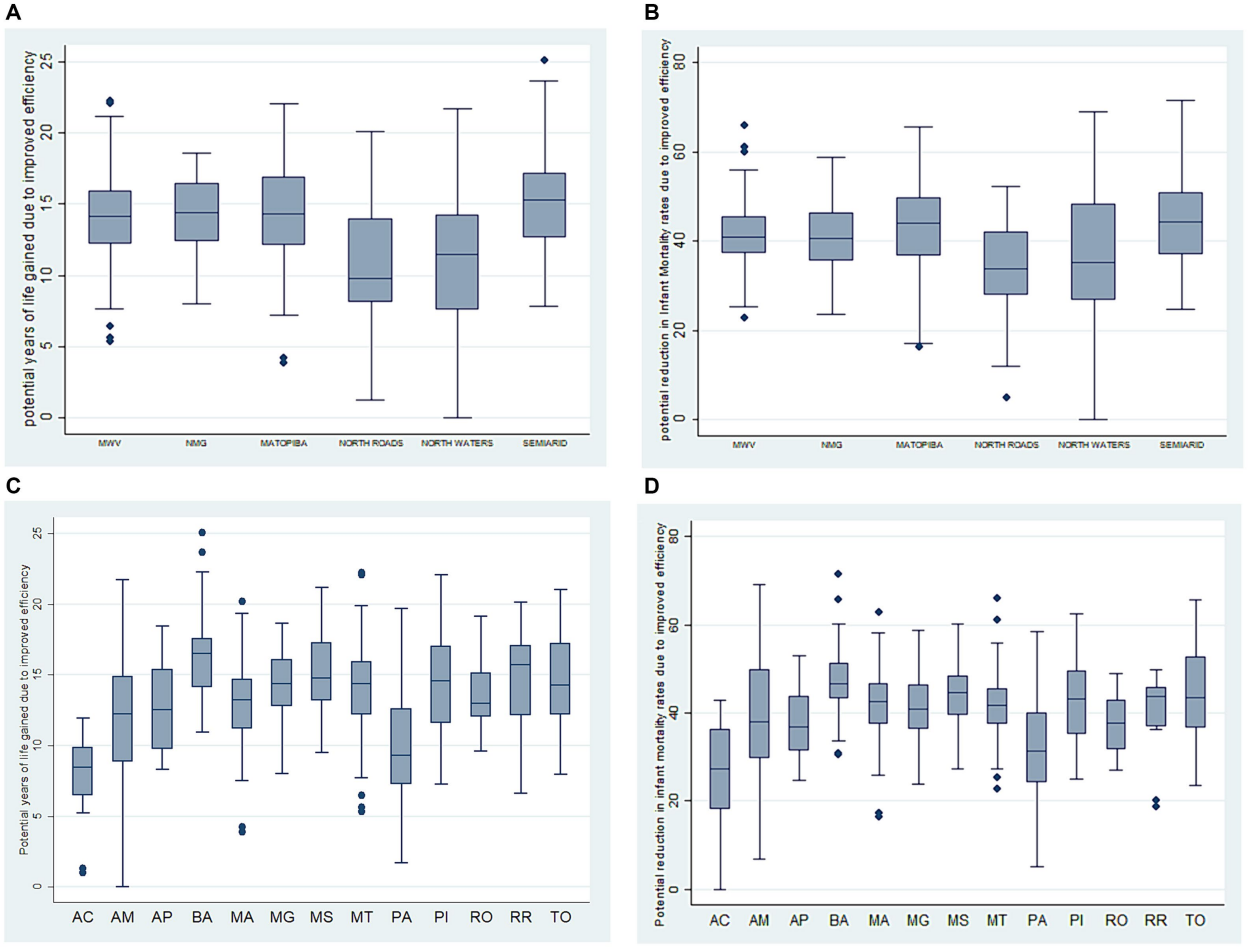
**(A)** Potential years of life gained, due to increased efficiency in the clusters of remote rural municipalities in Brazil. **(B)** Reduction in infant mortality rates due to efficiency gains in the clusters of remote rural municipalities in Brazil. **(C)** Potential years of life gained attributed to increased efficiency in Federative Units with remote rural municipalities in Brazil. **(D)** Reduction in infant mortality rates due to efficiency gains in the Federative Units of remote rural municipalities in Brazil.

The best efficiency results (LEB) were observed in the Amazon region, in the North Roads and Waters clusters, in that order, with relatively lower values of potential years of life gained by improved efficiency compared to the other typologies. Regarding the IM efficiency model, North Roads stands out along with the Northern Minas Gerais cluster, reaching a distribution close to North Waters. The others (MWV, MATOPIBA, and Semiarid) present higher potential reductions in infant mortality rates (Graphs 2A,B).

The best results are in Acre, Pará, Amazonas, and Amapá, which are part of the North Roads and North Waters Amazon clusters for both effect variables, with a reduced number of potential years of life gained and lower potential reductions in the infant mortality rate due to efficiency gains. The state of Rondônia, in the MWV, also stands out (Graphs 2C,D).

The inequalities in low income (% of the population receiving less than ¼ of a minimum wage) were higher for the MWV cluster (higher than the other typologies) and Matopiba, compared to the Semiarid. The inequalities in education by gender and ethnicity, on the other hand, were higher for the MWV and North Waters clusters compared to the remaining typologies, except for the North Roads region. Concerning income inequality by gender, the MWV presented the worst results, and the Northern Minas Gerais the best.

We may summarize our most important results below, depicting in which settings the RRL score higher than their counterparts:

**Table:** 

**RLL score better at resources and health levels** Level of resources and health were more efficient and effective in RRL states, scoring less at the intersectoral/global level	**RRL, compared to the other rural-urban typologies scored better overall**, with PHC models, basic sanitation, local health expenditures, income equality, employment, literacy, adequate prenatal care and adult mothers, and less education inequalities by gender/ethnicity
**RRL used less health services**, according to health needs, which was associated with low income and lower level of GDP (Public Administration)	**The best organizational models** were the FHS with CHW working in the territory, with home visits, prevention of acute respiratory infections and diarrhea and primary care physicians, with adequate birthweight
**Less inequities in RRL** Incidence of tuberculosis, hospitalizations due to sanitation-sensitive health causes, vaginal birth deliveries and smoking were influenced by ethnicity inequities in education and % of population with low income, average income inequities and % state GDP	**Best results in the Amazon region** Riverine and fluvial health teams, with diagnostic and therapeutic services, with less disparity in education levels, less teenage mothers, high % of Services GDP

Lastly, you may have a full view of all the results and the variables used in Supplementary material S8, including metadata, which can help the interested reader understand and reproduce this analysis. We also offer a summary table of all the efficiency analyses carried out and the variables remaining in the final models.

## 4 Discussion

The contrasts among Brazilian states bring difficulties inherent to their level of aggregation. At this level, the summary measures are difficult to interpret but also have some advantages, especially regarding the existence of more robust measurements in health when arising from epidemiological surveys, when compared to the local and global levels (32). Thus, our study assessed efficiency and effectiveness in health, with the possibility of including the utilization of health services according to health needs, as microdata from the National Health Survey.

Unlike the analyses performed at the global and local levels, we demonstrated the potential to dimension health more accurately. The differences observed shed light on the issue that healthcare is more closely related to health outcomes when related to health needs. In other words, by being disconnected from health needs, health actions do not contribute much to the health levels of society (6).

It is important to ponder that the results in the southeastern region do not mean that the states in this region are in a better situation than the others. We may deduce that in these places, the expected results are worse than the observed ones, such as the high levels of inequality, the incidence of tuberculosis, and unemployment. Thus, they could be much worse off than they are. This statement leads us to another reflection: are these places increasing their average results at the expense of gender and ethnic inequalities in income and education? Considering the contemporary capitalist development model, these inquiries make much more sense than we would like to admit (39).

The differences observed among the RRL states evidenced, in turn, greater efficiency in the dimensions of resources and especially health, with more promising results than intermediate and urban localities. Therefore, the models containing health expenditures, the % of GDP of the Public Administration, the density of nurses, and the proportion of low-income achieved better results in these localities. Concerning health, likewise, the final model showed better results in the states with RRL through the utilization of services according to health needs, the incidence of tuberculosis, the proportion of vaginal deliveries, smoking, and sanitation-sensitive hospitalizations.

We observed that reducing inequalities in income and education by ethnicity could confer efficiency and effectiveness gains in the societal health levels. Reducing the incidence of tuberculosis, which involves a series of socioeconomic structural measures, and increasing the possibilities of formal employment and guaranteed labor rights are fundamental steps on this path (40). We must consider that these inequities are exceedingly high in some Brazilian states, reaching more than twice in the case of income and almost four times in education. This unfair distribution affects all of society, involving those in a position of apparent privilege.

It is interesting to observe some continuity between remote rural, adjacent rural, and remote intermediate as if rurality and remoteness maintain some overlapping in remote rural municipalities. The most striking differences between rural and remote recently discussed by Wakerman et al. (3) were the geographical aspect, isolation, access to general and health services, smaller populations and of original peoples, differentiated care models with smaller, more locally integrated teams, and a broader scope of actions with difficulties of specialist support, relevant socioeconomic challenges, reduced power and more difficulty in obtaining the necessary resources.

According to research on intersectionality in health (41), “place” does not carry a unique meaning or singular experience for participants. Three themes related to place were the most prominent in their data set: (i) location, (ii) distance/proximity, and (iii) urban or rural situation. Place refers to the actual locations where care takes place, while distance/proximity refers to the relational aspect of place, for example, in terms of being near or far from health services. Situation refers to a sense of socially “positioned” or “situated” place. Intersectionality theory reminds us that these categorical findings are highly fluid and relational, and they complexly interrelate at various scales ranging from the body to overarching economic and sociopolitical structures (27).

Understanding place through an intersectional lens can increase the sophistication of the concept by raising questions about how researchers situate themselves in their research and develop, categorize, and understand the relationships between various types of places (28).

Intersectionality also increases the complexity of how identities are understood, made, undone, and simultaneously experienced in particular places. Similarly, a more sophisticated application of the concept of place to intersectional research can increase intersectionality’s appreciation of social constructions and meanings of place and its role in shaping processes of oppression and subject formation as it shapes places (28).

Furthermore, social constructions of meaning and power infuse places and integrate a complex web of intersecting categories of difference (e.g., cultural, economic, historical, and political). Hence, these intersections will ultimately shape places, social experiences, and contexts of social interactions. We must understand gender roles relationally to place, with material and symbolic consequences on rural women’s health. Gender and caregiving relations ultimately resonate with idealized views of the rural as an idyllic and feminine space, reinforcing and reproducing spaces of power and oppression (30, 42).

The remaining variables in the final RRL models point to the local reality for socio-environmental variables and those specific to healthcare models, such as basic sanitation with wells, springs, and septic. In these places, the general network is not always present and thorough, and the water supply and sewage collection occur in alternative ways, especially in the more distant regions. The health teams, the FHS, the CHW, and the fluvial and riverine teams stand out, as well as the importance of education and adequate prenatal care.

The remarkable theoretical framework of Bourke et al. (1), anchored in Giddens’ social structuring, already emphasized the importance of local health responses from health professionals, managers, and users in the structuring of the local health system, buoyed by health systems in a broader sense, i.e., the social practices and the set of protocols and norms that homogenize the sociocultural space.

Another relevant point is interculturality regarding teenage pregnancy in these localities, especially the more remote and rarefied, which acquires importance in the observed models (43, 44). Proper prenatal care becomes even more relevant—when we consider adolescent pregnancy, which is more prevalent in rural areas and vulnerable populations—to ensure maternal-fetal well-being during the perinatal period and afterward. The undesirable consequences, such as low birth weight, remain throughout these children’s lives.

The proportion of the services’ GDP reinforces the different populations and activities in rural and remote localities, which present different economic and sociocultural insertions and which have been advancing in the services sector, including technological innovations with agricultural, extractive, fishing, and handicraft activities. They may belong to traditional communities such as indigenous, “quilombolas” (maroon), and river dwellers who reside in rural settlements or work at the headquarters as salaried employees (5).

Strikingly, the Amazon clusters (North Roads/Waters) showed the best efficiency results, although they have immense difficulties with geographical accessibility and less favorable health indicators (45). However, considering their local realities, they always seek to deliver health care to their populations, no matter how dispersed they are, besides expanding their clinical performance, acquiring critical inputs, and performing exams in their territory when there is a pressing need.

Whereas North Roads presents itself as a new agribusiness frontier region with a more structured regional health network, North Waters has no interconnection to the intermediate urban network in part of the RRL and connects directly to the metropolis, Manaus (46). Both clusters present rarified populations, a higher proportion of traditional peoples, with distinct ways of life and socioeconomic insertions (4, 5).

The organization of life in Amazonas features the centrality of Manaus, around which the other municipalities disperse. The capital monopolizes life and the provision of services with low interaction among the municipalities, causing the remote population to reach Manaus for healthcare purposes. The model of inter-municipal relations is asymmetric, vertical, and fragile (46).

Our results show that these two clusters use their resources efficiently, combining care management strategies adapted to their socioeconomic, demographic, and cultural reality, even by necessity, in the face of the asymmetric model of municipal relations to all types of services.

Concerning infant mortality, the Northern Minas Gerais region also presented more favorable efficiency and effectiveness results than the other clusters. This performance is due in part to the results already achieved in this locality, despite its socioeconomic vulnerability (12), with better maternal and child health indicators, as well as the high coverage of the FHS in its territory (4, 5) and, mainly, by the higher equity of income and education by gender and ethnicity.

Regarding organizational models, it is evident that coverage by FHS teams and CHW determine the efficiency and effectiveness of health actions when comparing remote rural municipalities to others in the same Federative Unit. In this sense, Castro et al. (47) demonstrated, in a case study, greater cost-effectiveness in the FHS than in traditional PHC facilities.

However, when we evaluate the RRL only, the fluvial and riverine teams become more important and visible, as the models demonstrate. This result highlights the importance, in the RRL, of the teams moving to where the population lives, often surrounded by water and in areas of elevated population rarefaction, respecting interculturality.

The local availability of biochemical and imaging tests and therapeutic support are essential factors in the coordination of care by PHC and the perception of healthcare satisfaction by users (48). Their offer can make a big difference in these regions since the time spent, the excessive traveling needs and high cost of performing exams in another municipality, and or the acquisition of medicines in distant public or private facilities can affect the health status and the results achieved by health services.

The Amazon territories engender new reflections on the best choice of the organization model to face the local realities and the users’ needs. We should consider that the implementation of fluvial and riverine teams, as well as fluvial PHC facilities, mark relevant innovations in the way of organizing care management but introduces new needs for modifications in work processes and territorialization, with a family focus, markedly intercultural and anchored in health surveillance (10).

The investments required for its implementation may be higher than usual; however, the running costs are lower with more concrete results once the service adapts to the population and the use of the territory. Thus, these experiences can re-signify the best way to act in the territory: should the population be ascribed to the service, or should the service serve the territory? It reinforces the need for a strong, accessible, responsive, and proper (effective) PHC, offering continuity of care (49) and a community-territorialized approach that meets local needs (50).

In a case study in the Amazon region, Ferreira (51) defined the proportion of costs of a fluvial PHC facility, showing that most of them relate to human resources (64.62%), followed by inputs for health actions (17.72%), fuel (12.11%), and others (5.54%). We should draw attention to the potential efficiency gain from the performance of laboratory tests, at least the simplest ones, and from the displacement costs already planned and allocated in the scope of this kind of facility.

However, these arrangements require more planning and logistics to reach the fluid populations due to the activities and the water cycle with floods and ebb tides, characterizing challenges to overcome the distances and differentiated forms of displacement. Hence, the population’s lifestyles already incorporate these fluctuations, which may facilitate or hinder access to health services (45). They remain, therefore, to blend into health policies, with financial and logistical support to the municipalities, associated with the attraction of health professionals as they present greater efficiency and effectiveness in the collective health outcomes (52).

Our results agree with the studies reviewed, related to the importance of retaining human resources and establishing the best organizational models in PHC in order to gain equity (in access and quality) and efficiency. The models that take into account the geographical distances and local environmental characteristics proved to be more efficient. The farther the locality, the more mobile health teams and facilities appear to be the best alternative.

We have reported several limitations in our study along the way, either methodological (aggregation of data) or in interpretations (symbolic aspects of place), always seeking to overcome these difficulties with the lessons learned from our experience. We still lack more specific variables that may describe intersectional aspects more clearly, as well as customized measures for remote rural localities that may surpass the limits of the health production model (53). Our main strength is to study efficiency simultaneously with effectiveness through an equity lens that broadens our view and reduces distortions, filling an important gap in the literature.

The main limitation of our study regards the use of secondary/aggregated data created for different purposes, coming from different databases and timeframes. We have treated the data accordingly, with control of time invariant characteristics, with the fixed effects models.

Future research should delve with a mixed methods frame, with more contextual information and capacity to build more robust indicators that capture the local reality, with primary data, allowing measuring more directly the efficiency/effectiveness of local health systems.

## 5 Conclusion

Brazilian states with RRL show superior resource and health efficiency through utilization of services according to health needs. These models showed strong determination and the final models included intersectional equity. The main message is that reducing intersectional inequities in income and education by ethnicity could greatly increase the efficient attainment of health levels in society. The remote rural typology demonstrated greater efficiency and effectiveness in health than the other typologies in the states containing RRL. The organizational models with the FHS teams and the CHW visits played a key role, together with *per capita* health expenditures and intergovernmental transfers. Among the remote rural municipalities, the Amazon region clusters (North Roads/Waters) stand out, with models presenting variables relevant to local reality, such as the coverage of fluvial and riverine teams, and especially intersectional gender and ethnicity equity in education.

Hopefully, our findings shall contribute to gains in efficiency and effectiveness in these spaces, through health policies prioritizing financial resources (local and from other levels of government) and the settlement of health professionals, family health teams configured for the necessary displacements, the availability of local diagnosis and treatment, and closer attention to basic sanitation and intersectional inequities in education. Finally, we must aim for equity of gender and ethnicity in income and education, and above all of place, perceived in its entirety.

## Data availability statement

Datasheets are also available as Supplementary materials. The original contributions presented in the study are included in the article/Supplementary material, further inquiries can be directed to the corresponding author. The consulted data sources are open to public access and can be reached at the following websites: IBGE—Brazilian Institute of Geography and Statistics (demographic and health surveys), https://www.ibge.gov.br/estatisticas/sociais/populacao/9662-censo-demografico-2010.html?=&t=o-que-e, https://www.ibge.gov.br/estatisticas/sociais/saude/9160-pesquisa-nacional-de-saude.html; DATASUS—SUS Information Technology Department (CIH, CNES, SIA, SIM, SINAN, SINASC; SIAB; SISMAMA; SISCOLO), https://datasus.saude.gov.br/informacoes-de-saude-tabnet/; e-gestor AB—e-manager-PHC, https://acesso-egestoraps.saude.gov.br/login; MDS—Social Development Ministry, https://legado.dados.gov.br/dataset?_license_id_limit=0&organization=ministerio-do-desenvolvimento-social-mds&res_format=JSON&license_id=cc-by&tags=mi+social&_res_format_limit=0; MPF—Federal Prosecution Ministry, http://combateacorrupcao.mpf.mp.br/ranking; SIOPS—Public Health Budget Information System, http://siops-asp.datasus.gov.br/cgi/siops/serhist/MUNICIPIO/indicadores.HTM; STN/MF—National Treasure Department/Ministry of Finance, https://www.gov.br/tesouronacional/pt-br; UNDP—United Nations Development Program, https://www.undp.org/pt/brazil/idhm-munic%C3%ADpios-2010.

## Ethics statement

Obtaining ethic approval is not applicable for this postdoctoral project, as publicly available data was used in this study. The main research, which this project relates to, was approved by the Ethics Committee of the Faculty of Public Health of the University of São Paulo under CAAE number 37672620.8.0000.5421, statement 4.285.824, as of September, 18th, 2020.

## Author contributions

SS: Conceptualization, Data curation, Formal analysis, Funding acquisition, Investigation, Methodology, Project administration, Writing – original draft, Writing – review & editing. AB: Conceptualization, Funding acquisition, Supervision, Validation, Writing – review & editing.

## Supplementary material

Supplement 1 Statistical techniques in more detail (DEA and FE).

Supplement 2 Rural-urban classifications and typologies (IBGE).

Supplement 3 Typologies of the Brazilian remote rural localities.

Supplement 4 Bivariate efficiency analysis by dimension-all Brazilian states analysis.

Supplement 5 Graphs by macro-regions and Federative Units-all Brazilian states efficiency analysis and more details about the Brazilian states.

Supplement 6 Bivariate analysis by dimension - Brazilian states with RRL only.

Supplement 7 Graphs by rural-urban typologies for Brazilian states with RRL only. Ranking of the Brazilian states, according to rural-urban typology.

Supplement 8 Summary chart of variables in the final models for the three analysis and metadata.

Supplement 9-12 Datasheets with information on the three analyses performed.
