# Supplementary material for: Intersectional equity in Brazil’s remote rural municipalities: the road to efficiency and effectiveness in local health systems
Source: Front Public Health. 2024 Sep 10;12:1401193. doi: 10.3389/fpubh.2024.1401193 (PMC11419982; doi:10.3389/fpubh.2024.1401193)
Supplement: Supplementary file 1 [file Table_1.DOCX]

**Supplement 1- Statistical techniques in more detail (DEA and FE)**

**S1.1 Data Envelopment Analysis (DEA) models:**

The slack-based DEA model (DEA-SBM), proposed by Tone in 2002, considers the production model's stages, a fundamental issue worked on in this article. A limitation of DEA models is that they neglect the internal stages of each Decision-Making Unit (DMU) and the linking activities between them. In network models, the interconnection between activities is an indispensable feature with the analysis carried out dynamically in multiple stages to overcome the restrictions of static models and bring the analysis closer to the characteristics of real systems (Mariz, 2015). Thus, for a DMU to be efficient, it must be efficient at all stages of its production process; detecting non-radial inefficiencies. In this type of approach, intermediate products are produced and consumed within the DMU while inputs and outputs are exogenous to its structure. In addition to the overall score, the network model allows the calculation of efficiency indices for each stage. Another feature is that the production possibility set (PPS) is modeled progressively, with its own technological level, and with specific reference coefficients (Cook and Zhu, 2014). *Max Dea 8 Ultra* software was used to carry out the network and slack analyses in a dynamic model.

Estimating the efficiency of a DMU (x0, y0) is obtained by solving the following linear programming problem, considering the model based on gaps:

$$\rho= \frac{1-\frac{1}{m}.\sum_{i=1}^{m} s_{i}^{-}/x_{i0}}{1+\frac{1}{s}.\sum_{r=1}^{s} s_{r}^{+}/y_{r0}}$$

Subject to:

xo = Xλ+ s- (input slack vector)

y0 = Yλ- s+ (output slack vector)

λ≥ 0, s- ≥ 0, s+ ≥ 0 (λ is the intensity vector)

where *m* refers to the inputs and *s* refers to the outputs.

The breakdown of the previous equation, in the product-oriented model with k steps results in:

$$\frac{1}{\tau_{0}^{*}}= max \Sigma_{k=1}^{K}W^{k}⟦1+ \frac{1}{r^{k}+ \sum_{h \in F_{k}} t_{k, h)}}(\frac{\sum_{kr=1}^{r} s_{r0}^{k+}}{y_{r0}^{k}}+\frac{\Sigma_{h \in F_{k}s_{h0}^{\left( k,h \right)+}}}{z_{h0}^{(k,h)}}⟧$$

Subject to:

z ho(k, h) = Z(k, h) λk - s o(k, h)+

Z(k, h) λh =Z(k, h) λk

s o(k, h)+≥ 0

where, *w_k_* is the relative weight of each division; *F_k_* is the set of stages with links (k, h); ∑K_k_=1 wk =1; w_k_ ≥ 0; s_k_+ are the output slack vectors; *r_k_* is the number of outputs in stage k; t _(k, h)_ is the number of products in the link between stage k and h; *s_ho_^(k, h)+^* are the slack vectors of the links and *z* deals with the intermediate products.

**S1.2 Fixed effects models - panel data**

The fixed effects regression model for panel data has been widely used by researchers, with the possibility of including confounding and interaction variables in the effectiveness analysis, which considers the regression's beta coefficients. The following assumptions apply: each unit (municipality or state) has its own characteristics which may or may not influence the explanatory variables; some attributes of the unit may impair the explanatory power of the variables and it is therefore necessary to control for this effect. The fixed effects model removes these time-invariant characteristics from the explanatory variables in order to analyze their net effect. The location-specific effect was calculated by adding the fixed effect to the output residuals, which can denote inefficiencies at their upper limit when negative, relativizing the untested, non-existent or unavailable variables and measurement errors (Joumard et al., 2008). A relevant premise is that these individual and time-invariant characteristics are specific to the unit and do not correlate with each other. Each unit is distinct, so the error term and the constant (which captures these characteristics) should not correlate with those of the other units (Woolridge, 2006). We also tested serial autocorrelation and heteroscedasticity in the residuals of the regressions estimated using the ordinary least squares method. When necessary, we performed corrections, and regressions based on the generalized least squares method were used. The analyses were carried out using *Stata SE 14.0* software.

The equation below was used to obtain the final models:

Yit=β_o_+ β_1_ X_(1,it)_,+⋯+ β_k_ X_(k,it)_ +y_2_ E_2_+⋯+y_n_ E_n_+ δ_2_ T_2_+ δ_t_ T_t_+μ_i t_

Where:

-Y_it_ is the dependent variable (DV) where i = unit and t = time

-X _k,it_ represents the independent variables (VI),

-β_k_ is the coefficient for the VIs,

-u_it_ i is the error term

-ɛ_n_ is the unit n.

-γ_2_ is the coefficient for the units.

-T_t_ is the time.

-δ_t_ is the coefficient relative to time.

**REFERENCES**

Cook WD, Zhu J, editors. Data Envelopment Analysis: A Handbook of Modeling Internal Structure and Network. New York: Springer (2014).

Joumard I, André C, Nicq C, Chatal O. Health status determinants: lifestyle, environment, health care resources and efﬁciency. Paris: Organisation for Economic Cooperation and Development, (2008).

Mariz FBAR (2015). Modelos dinâmicos de análise envoltória de dados: revisão da literatura e comparação de modelagens [dissertation]. [Natal (RN]: Centro de Tecnologia da Universidade Federal do Rio Grande do Norte.

Tone K. A slack-based measure of efﬁciency in data envelopment analysis. *Eur J Oper Res*. (2002) 130(3): 498–509.

Woolridge JM. Introdução à econometria: uma abordagem moderna. 4. ed. São Paulo: Thomson Pioneira. (2006).
