# Supplementary material for: Intersectional equity in Brazil’s remote rural municipalities: the road to efficiency and effectiveness in local health systems
Source: Front Public Health. 2024 Sep 10;12:1401193. doi: 10.3389/fpubh.2024.1401193 (PMC11419982; doi:10.3389/fpubh.2024.1401193)
Supplement: Supplementary file 2 [file Table_2.DOCX]

**Supplement 2- IBGE rural-urban classifications and typologies**

**On the rural-urban typologies used**

The typology proposed by the Brazilian Institute of Geography and Statistics-IBGE (2017) was based on the following premises: population units with more than 50,000 inhabitants in areas of dense occupation or which were part of urban concentrations were classified as predominantly urban; while population units with a population in areas of dense occupation of less than 3,000 inhabitants were classified as predominantly rural. After defining the initial criteria, an analysis was made of the interrelationship between the classification obtained according to the total population in areas of dense occupation and the percentage of the population in areas of dense occupation of the population units.

Thus, predominantly urban or rural municipalities and intermediate municipalities were classified (IBGE, 2017).

a) Predominantly urban municipality: municipalities with more than 50,000 inhabitants in areas of dense occupation; or which have between 25,000 and 50,000 inhabitants in this area, with a degree of urbanization greater than 50%; or with 10,000-25,000 inhabitants in this area with a degree of urbanization greater than 75%.

b) Intermediate municipality: municipalities with 25,000 to 50,000 inhabitants in a densely populated area with a degree of urbanization between 25 and 50%; or with 10,000-25,000 inhabitants in this area with a degree of urbanization between 50 and 75%; or with 3,000-10,000 inhabitants in this area with a degree of urbanization above 75%.

c) Predominantly rural municipality: municipalities with 25,000 to 50,000 inhabitants in a densely populated area with a degree of urbanization of less than 25%; or with 10,000-25,000 inhabitants in this area with a degree of urbanization of less than 50%; or with 3,000-10,000 inhabitants in this area with a degree of urbanization of less than 75%.

In addition, the location dimension was assessed in order to differentiate, among the municipalities classified as intermediate and rural, those adjacent to higher-ranking urban centers from those that are more distant or remote (IBGE, 2017).

Thus, 76.0% of the Brazilian population were in municipalities considered predominantly urban, corresponding to only 26.0% of all municipalities. In contrast, the majority of municipalities were classified as predominantly rural (60.4%), with 54.6% as adjacent rural and 5.8% as remote rural (IBGE, 2017).

Brazilian macro-regions

In the North region, 10.5% of the population lives in municipalities classified as remote rural, corresponding to 26.9% of municipalities. In the Northeast, 33% of the population lived in rural municipalities, with the lowest percentage of population in urban municipalities (59.0%). The Southeast had the highest percentages of municipalities and population in urban municipalities (87%) and the lowest proportions in rural municipalities. The South Region has the lowest relative values for municipalities classified as remote (intermediate or rural), with less than 0.5%, denoting the shortest distances between municipal headquarters. The Midwest is the region with the second largest population in urban municipalities (79.8%), as well as in remote rural municipalities (4.0%) (IBGE, 2017).

**Reference**

Instituto Brasileiro de Geografia e Estatística, Coordenação de Geografia. Classificação e caracterização dos espaços rurais e urbanos do Brasil: uma primeira aproximação. Rio de Janeiro: IBGE (2017)
