# Supplementary material for: Intersectional equity in Brazil’s remote rural municipalities: the road to efficiency and effectiveness in local health systems
Source: Front Public Health. 2024 Sep 10;12:1401193. doi: 10.3389/fpubh.2024.1401193 (PMC11419982; doi:10.3389/fpubh.2024.1401193)
Supplement: Supplementary file 3 [file Table_3.DOCX]

**Supplement 3-Typologies of the Brazilian remote rural localities**

**The typology of remote rural Brazilian municipal agglomerations – Clusters**

**Remote rural cluster map-distribution across municipalities**

**Clusters:** 1 Midwest Vector (MWV); 2 – Northern Minas Gerais (NMG); 3 – Matopiba; 4 – North Roads (NR); 5 – North Waters (NW) e 6 – Semiarid (SA)

The typology above was developed based on the analytical category of how we use the territory - how, where, for whom, why, for what - in the study of Santos and Silveira (2001). These authors identified four regions: the concentrated (South and Southeast), the Recent Peripheral Occupation, the Northeast and the Amazon. Bousquat et al. (2022) then classified the remote rural municipalities (RRM) according to their logic of insertion into the economic circuit and their predominant mode of interconnection with the other points of the territories, whether by land or river (Bousquat et al., 2022).

The MRRs were then scrutinized and disaggregated by variables that denote the rarefaction and remoteness of the population, in addition to economic capacity. The qualitative analysis led to the design of six clusters (313 MRR or 97% of the total) called: MATOPIBA (92); Northern Minas Gerais-NMG (22); Midwest Vector-MWV (84); Semiarid (42); North Waters (45); and North Roads (28) (Bousquat et al., 2022).

In the Northeast, two clusters were identified, the Semiarid and MATOPIBA, with different insertions in the national economy. MATOPIBA, an area of the *cerrado* biome, is the acronym for the initials of four states: Maranhão-MA, Tocantins-TO, Piauí-PI and Bahia-BA and represents a new agricultural frontier. The Semiarid, on the other hand, is a vulnerable area of ancient occupation and a region marked by drought, with economic, social and environmental impacts. Similarly, the Northern region of Minas Gerais (NMG) is similar to the Semiarid but with better climatic conditions, considered a less vibrant zone within the more concentrated Southeast region of Brazil (Bousquat et al., 2022; Fausto et al., 2023).

The Midwest vector-MWV is the most integrated agglomeration, as it has excelled in agribusiness culture, especially soy, and has an importance in the global economic circuit. The Northern Region is a forest biome, characterized by low population and technical densities, with many conflicts. We have two distinct realities: one based on the dynamics of rivers – North Waters - for all kinds of services, and the other more linked to roads and the new agribusiness transportation – North Roads (Bousquat et al., 2022; Fausto et al., 2023).

**References**

Santos M, Silveira ML. O Brasil: território e sociedade no início do século XXI. Rio de Janeiro: Record. (2001).

Bousquat A, Fausto MCR, Almeida PF de, Lima JG, Seidl H, Sousa ABL, et al. Remoto ou remotos: a saúde e o uso do território nos municípios rurais brasileiros. *Rev Saúde Pública*. (2022) 56:73.

Fausto MCR, Almeida PF; Bousquat A, Santos AM, Giovanella L. Atenção Primária à Saúde em territórios rurais remotos no Brasil. Rio de Janeiro: Fiocruz (2023)
