# Supplementary material for: Intersectional equity in Brazil’s remote rural municipalities: the road to efficiency and effectiveness in local health systems
Source: Front Public Health. 2024 Sep 10;12:1401193. doi: 10.3389/fpubh.2024.1401193 (PMC11419982; doi:10.3389/fpubh.2024.1401193)
Supplement: Supplementary file 4 [file Table_4.DOCX]

**Supplement 4- Bivariate efficiency analysis by dimension-all Brazilian states analysis**

**Chart S4.1** - Bivariate analysis for life expectancy and infant mortality effect variables: resource dimension, fixed effects model. Brazil, 2010-19, all states analysis.

| **Resources dimension**  **Variable** | **Life expectancy at birth**  **beta (p)** | **Infant mortality**  **beta (p)** |
| --- | --- | --- |
| **GDP per capita** | 0,0004 (<0,001) | -0,0010 (<0,001) |
| **State spending as % GDP** | 6,3940 (<0,001) | -21,6624 (<0,001) |
| **Health expenditure as % GDP** | 4,4626 (0,003) | -16,9967 (<0,001) |
| **Medical doctors - density** | 9,7708 (<0,001) | -27,4074 (<0,001) |
| **Nurses- densty** | 5,2642 (<0,001) | -14,9312 (<0,001) |
| **Density of computorized tomography** | 0,0275 (0,003) | -0,0738 (0,015) |
| **Density of magnetic ressonance** | 0,0322 (0,005) | -0,0834 (0,035) |
| **Density of PHC facilities**  **FHS teams** | 0,0120 (<0,001)  0,0094 (0,007) | -0,0407 (<0,001)  -0,0248 (0,028) |
| **PHC % funding** | -0,1901 (<0,001) | 0,5864 (<0,001) |
| **Agriculture (%GDP)** | 5,81 e -07 (0,003) | -1,66 e -06 (0,008) |
| **Industry (%GDP)** | 6,52 e -08 (0,014) | -1,68 e -07 (0,052)** |
| **Services (%GDP)** | 2,52 e -08 (0,061)** | -6,44 e -08 (0,138) |
| **Taxes (%GDP)** | 6,97 e -08 (0,043) | -1,78 e -07 (0,1110) |
| **Public Administration (%GDP)** | 1,78 e -07 (0,001) | -4,86 e -07 (0,0 |
| **Population earning <1/2 MW (%)** | -0,3512 (<0,001) | 1,0746 (<0,001) |
| **Population earning <1/4 MW (%)** | -0,3985 (<0,001) | 1,2998 (<0,001) |
| **Average per capita income** | 0,0195 (<0,001) | -0,0539 (<0,001) |

**Source:** DATASUS- Information Technology Department of the Public Healthcare system; IBGE- Brazilian Institute of Geography and Statistics, SIOPS/MS-Information System for Public Budget in Health/Ministry of Health and STN/MF (National Treasury Secretary/ Ministry of Finance);

**Note:** In green, results associated with improvement; in red, with worsening; in black, not significant.

**Chart S4.2** - Bivariate analysis for life expectancy and infant mortality effect variables: intersectoral dimension, fixed effects model. Brazil, 2010-19, all states analysis.

| **Intersectoral dimension**  **Variable** | **Life Expectancy at birth**  **beta (p)** | **Infant mortality**  **beta (p)** |
| --- | --- | --- |
| **Gini index** | -119,8292 (<0,001) | 369,6582 (<0,001) |
| **% adolescent mothers**  **% children out of school (0-5 y)**  **% vulnerable popultion without electrical power** | -6,5377 (<0,001)  -0,3180 (<0,001)  -0,5208 (<0,001) | 18,2571 (<0,001)  0,9451 (<0,001)  1,6877 (<0,001) |
|  |  |  |
| **Ratio average income**  **Gender**  **Ethnicity**  **Income inequality by gender/race** | 150,1455 (0,006)  -24,1405 (<0,001)  7,2422 (<0,001) | -498,8751 (0,004)  73,6989 (<0,001)  -21,9373 (<0,001) |
| **HDI** | 45,6381 (<0,001) | -140,9991 (<0,001) |
| **Ratio - dependency**  **Ageing index** | -0,5145 (<0,001)  3,8601 (<0,001) | 1,5932 (<0,001)  -11,4840 (<0,001) |
| **Basic sanitation**  **Water supply**  **Inadequate sanitary facilities**  **Garbage collection** | 0,2089 (<0,001)  -0,7799 (<0,001)  0,4257 (<0,001) | -0,6542 (<0,001)  2,2850 (<0,001)  -1,3381 (<0,001) |
| **Ratio % highly educated**  **Gender**  **Ethnicity**  **Inrquality gender/race** | -24,7710 (<0,001)  -6,7118 (<0,001)  39,4841 (0,005) | 74,8504 (<0,001)  22,0206 (<0,001)  -101,0621 (0,030) |
| **Illiteracy rate** | -1,2371 (<0,001) | 4,0142 (<0,001) |
| **Unemployment rate**  **Formal employment** | -0,8618 (<0,001)  0,6555 (<0,001) | 2,5352 (<0,001)  -1,9835 (<0,001) |
| **Transparency** | 1,5282 (0,001) | -4,3049 (0,004) |

**Source:** DATASUS- Information Technology Department of the Public Healthcare system; IBGE- Brazilian Institute of Geography and Statistics, SIOPS/MS-Information System for Public Budget in Health/Ministry of Health and STN/MF (National Treasury Secretary/ Ministry of Finance); UNDP-United Nations’ Development Programme/BRAZIL and MPF (Federal Public Ministry).

**Note:** In green, results associated with improvement; in red, with worsening; in black, not significant.

**Chart S4.3** - Bivariate analysis for life expectancy and infant mortality effect variables: health dimension, fixed effects model. Brazil, 2010-19, all states analysis.

| **Health dimension**  **Variable** | **Life expectancy at birth**  **beta (p)** | **Infant mortality**  **beta (p)** |
| --- | --- | --- |
| **Health services utilization (observed)** | 0,2997 (<0,001) | -0,9014 (<0,001) |
| **Health services utilization (expected)**  **Expected x Observed** | 1,5739 (<0,001)  -1,2872 (0,002) | -4,6784 (<0,001)  3,9300 (0,004) |
| **FHS household registration** | 0,5166 (<0,001) | -1,5161 (<0,001) |
| **Prevalence SAH** | 1,6516 (<0,001) | -5,0472 (<0,001) |
| **Limitation SAH** | -1,4856 (0,001) | 5,0272 (<0,001) |
| **Prevalence DM** | 2,9050 (<0,001) | -9,3105 (<0,001) |
| **Limitation DM** | -0,4007 (0,060)** | 1,2460 (0,067)** |
| **Comparison General Health Status (worse/better)** | -0,9545 (0,003) | 2,5370 (0,0140) |
| **Healthy diet** | -0,2270 (<0,001) | 0,6616 (<0,001) |
| **Alcohol – prevalence**  **Smoking - prevalence** | -0,8799 (0,005)  -1,6920 (<0,001) | 3,2822 (0,001)  5,4463 (<0,001) |
| **Sedentary lifestyle- prevalence** | -0,5449 (<0,001) | 1,4291 (0,003) |
| **Coverage Mental Health-CAPS** | 10,9693 (<0,001) | -34,6471 (<0,001) |
| **Hospitalization due to sensitive conditions**  **related to PHC**  **related to Basic sanitation** | -1,4700 (<0,001)  -2,2339 (<0,001) | 4,5613 (<0,001)  6,9470 (<0,001) |
| **Breast câncer screening** | 35,5604 (<0,001) | -117,4603 (<0,001) |
| **Cervical câncer screening** | -20,3825 (<0,001) | 62,5384 (<0,001) |
| **Newborns with 7 or more prenatal appointments** | 0,2953 (<0,001) | -0,8798 (<0,001) |
| **Consultations per inhabitant** | 5,3220 (<0,001) | -16,0468 (<0,001) |
| **Hospitalization (per 100 inhab)** | -2,7480 (<0,001) | 8,2221 (<0,001) |
| **Incidence of Tuberculosis** | -0,3497 (<0,001) | 0,9797 (<0,001) |
| **Vaccination - general (% coverage)** | 0,0228 (0,864) | -0,0603 (0,887) |
| **Preventable deaths <5yª** | -0,0024 (<0,001) | 0,0074 (<0,001) |
| **% vaginal deliveries** | -0,5832 (<0,001) | 1,8667 (<0,001) |

**Source:** CNES - National Register of Health Establishments; SIA - Outpatient Information System; SIAB - Primary Care Information System; SIH - Hospital Information System; SINAN - Notifiable Diseases Information System; SINASC - Live Births Information System; SIM - Mortality Information System; SISCOLO and SISMAMA - cervical and breast cancer information systems; PNI - National Immunization Program.

**Note:** In green, results associated with improvement; in red, with worsening; in black, not significant.
