# Supplementary material for: Intersectional equity in Brazil’s remote rural municipalities: the road to efficiency and effectiveness in local health systems
Source: Front Public Health. 2024 Sep 10;12:1401193. doi: 10.3389/fpubh.2024.1401193 (PMC11419982; doi:10.3389/fpubh.2024.1401193)
Supplement: Supplementary file 5 [file Table_5.docx]

**Supplement 5-Graphs by macro-regions and Federative Units-all Brazilian states efficiency analysis and more details about the Brazilian states**

**Graph S5.1** - Potential years of life gained by increased efficiency by macro-region and Federative Unit (state), 2010-19, Brazil.


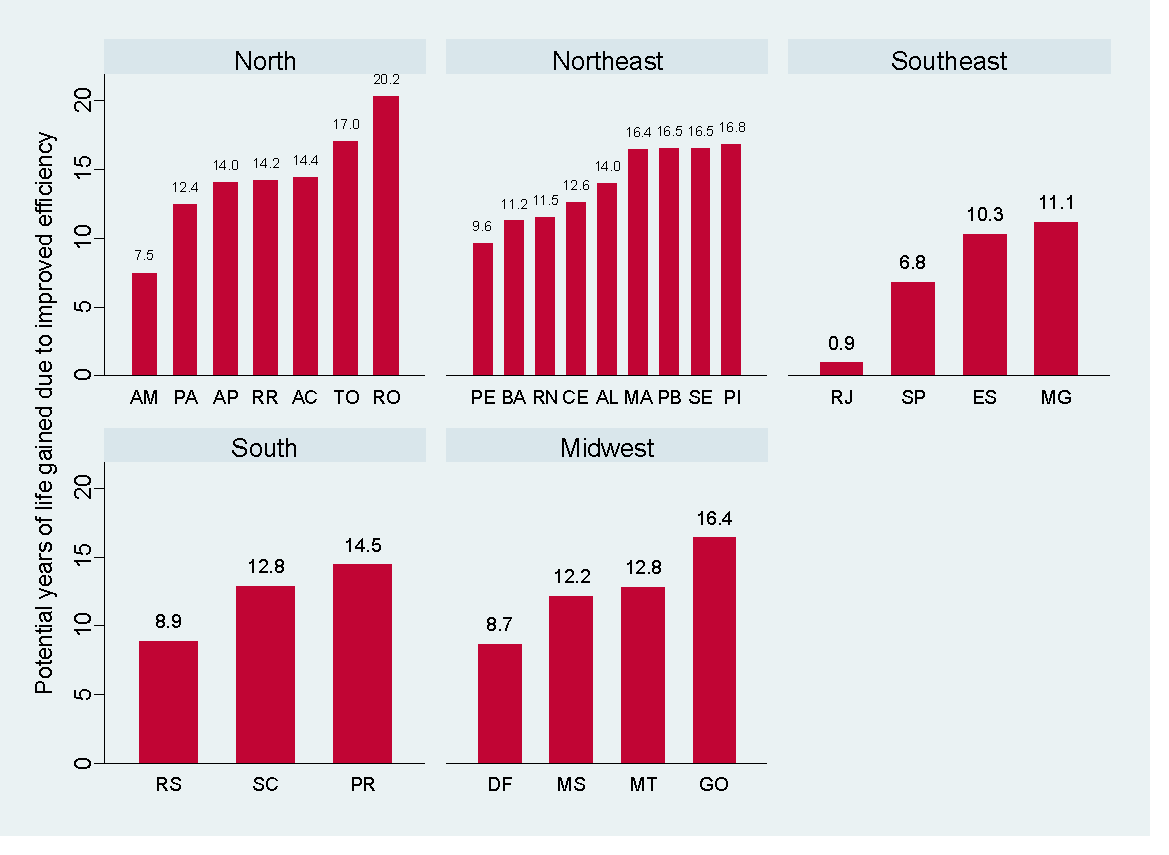


**Graph S5.2** - Potential years of life gained due to increases in efficiency by Federative Unit, 2010-19, Brazil.


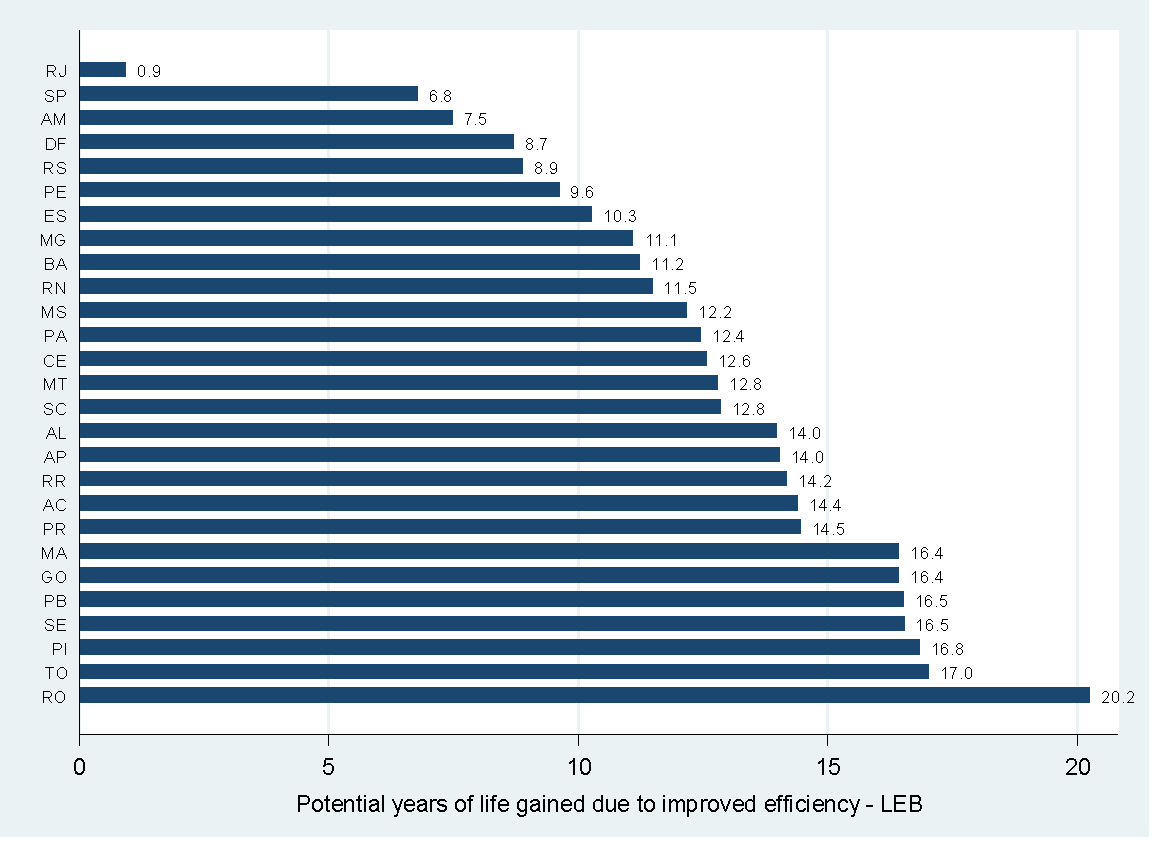


**Graph S5.3** - potential reduction in infant mortality rate due to efficiency gains, by macro-region and Federative Unit, 2010-19, Brazil.


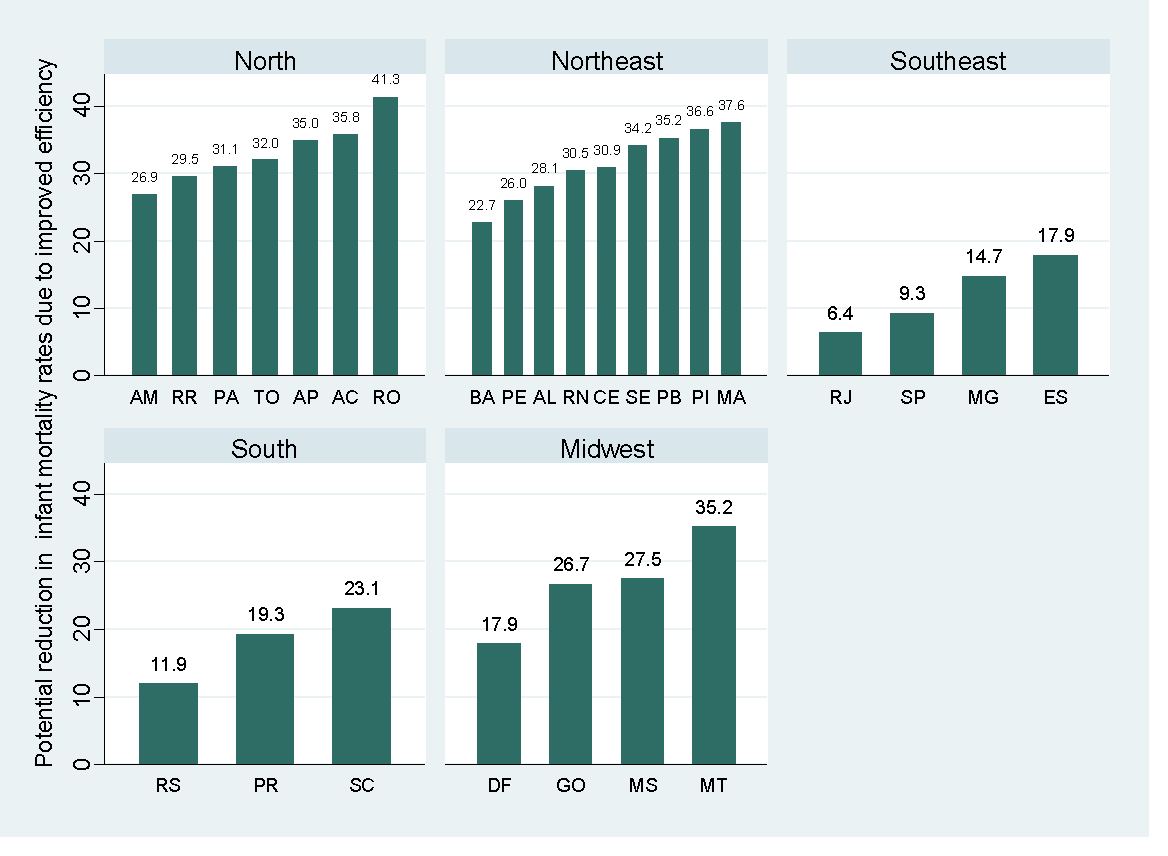


**Graph S5.4**-Potential reduction in the infant mortality rate attributable to efficiency gains by state, 2010-19, Brazil.


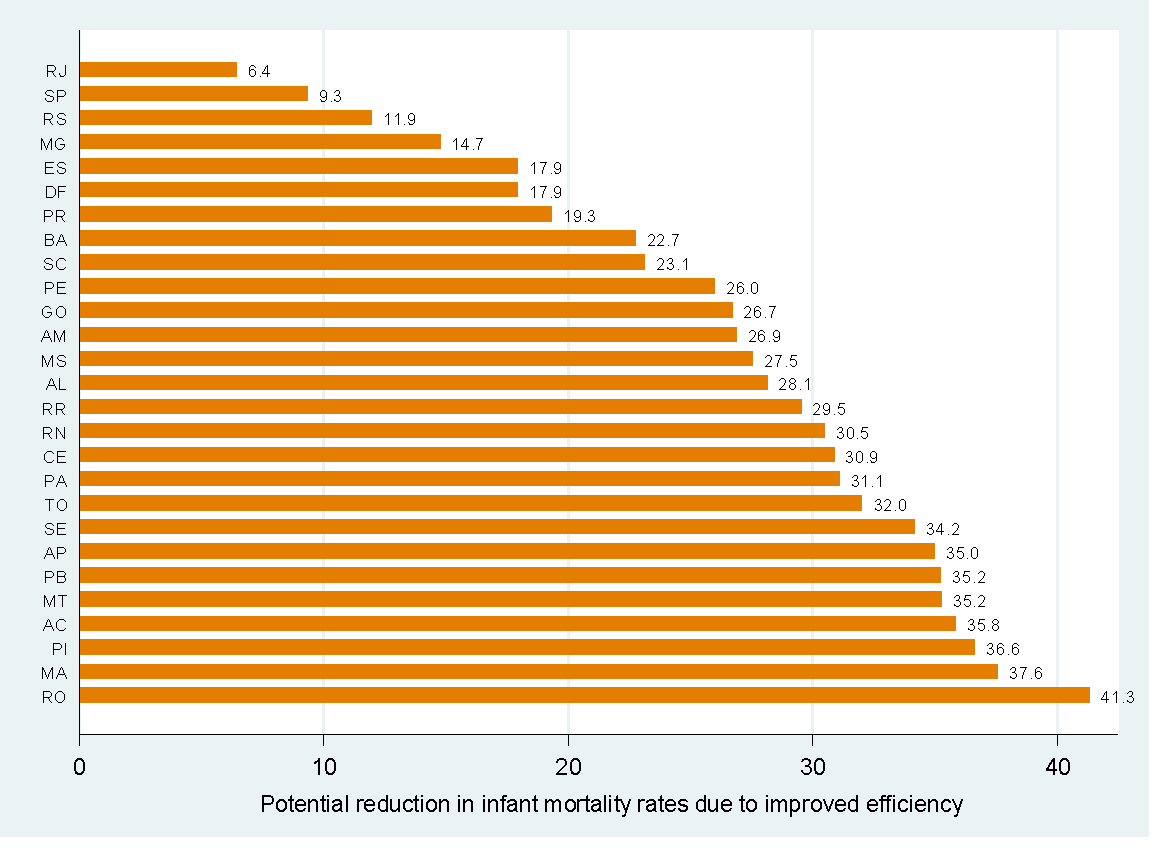


**Graph S5.5** - Potential years of life gained by increased efficiency in the resource dimension by macro-region and Federative Unit, 2010-19, Brazil.


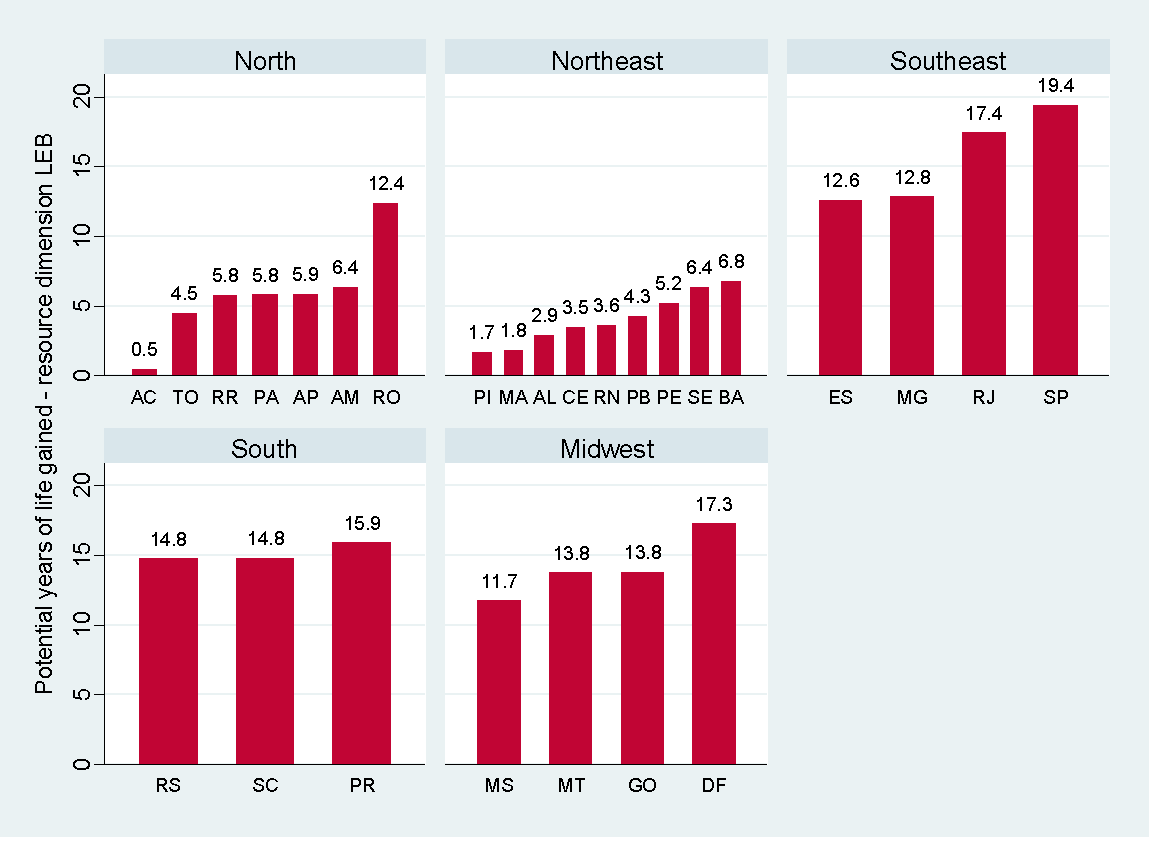


**Graph S5.6** - Potential years of life gained by increased efficiency in the resource dimension by Federative Unit, 2010-19, Brazil.


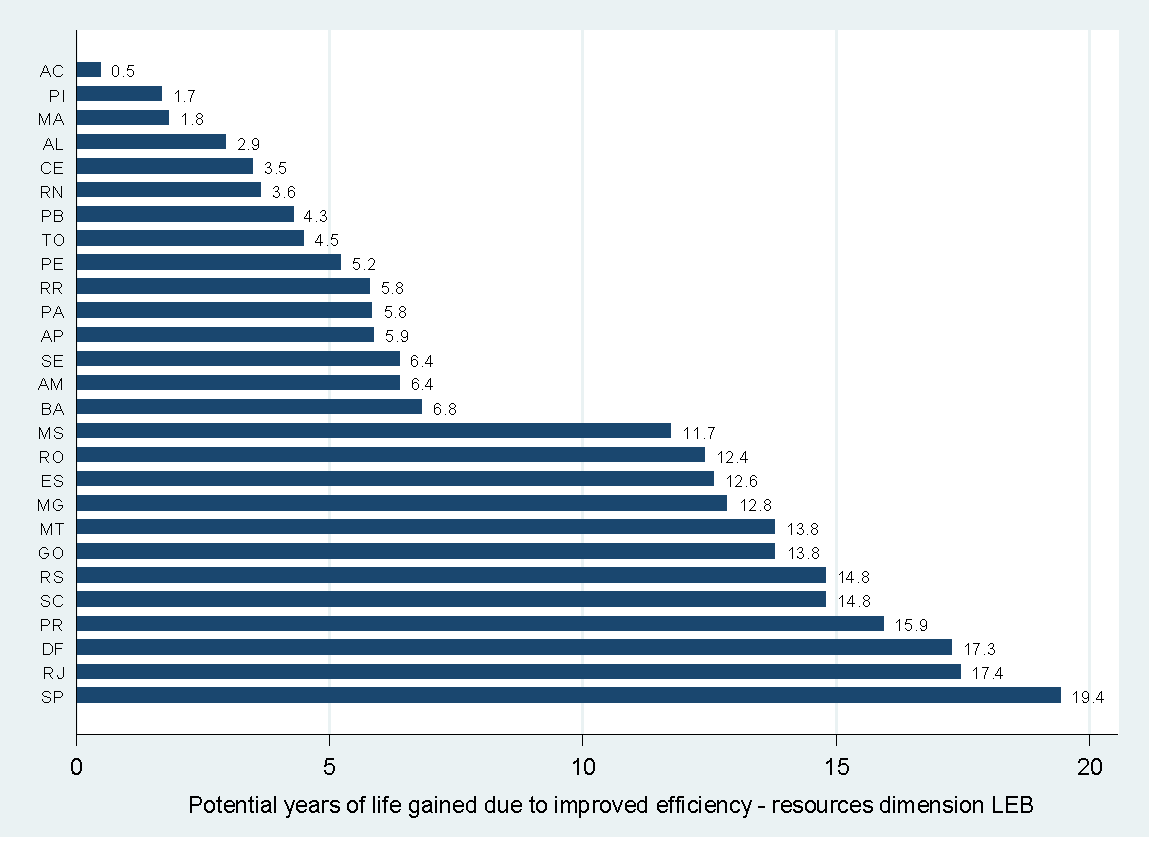


**Graph S5.7** - Potential reduction in infant mortality rate due to increased resource efficiency by macro-region and Federative Unit, 2010-19, Brazil.


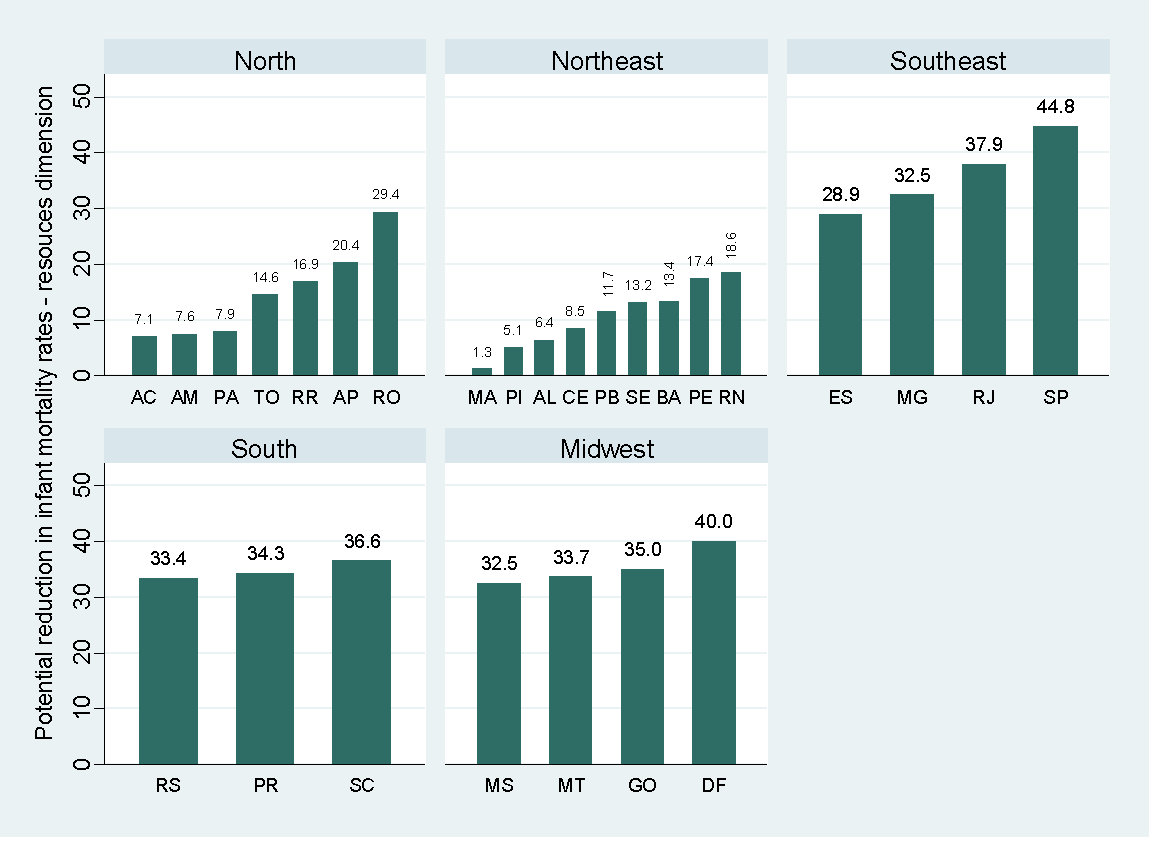


**Graph S5.8** - Potential reduction in infant mortality rate due to increase in resource efficiency by Federative Unit, 2010-19, Brazil.


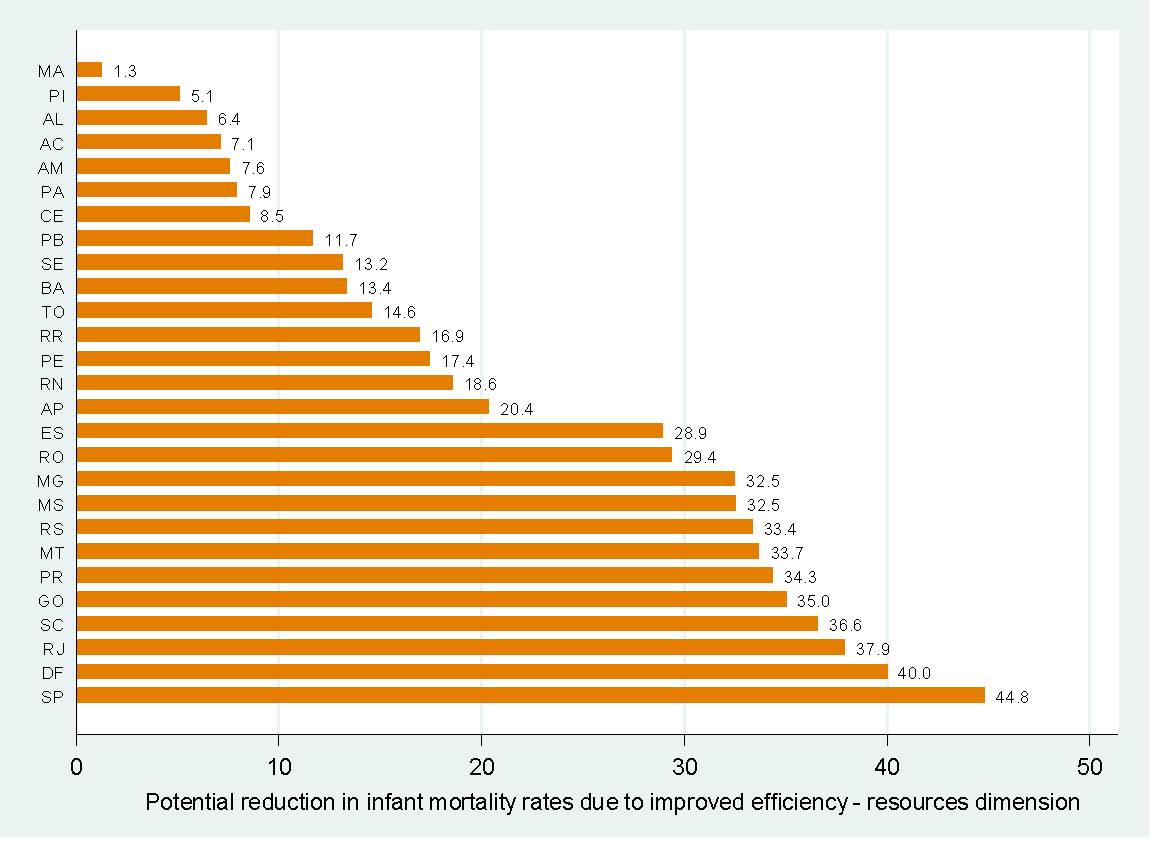


**Graph S5.9-** Potential years of life gained by increased efficiency in the health dimension by macro-region and Federative Unit, 2010-19, Brazil.


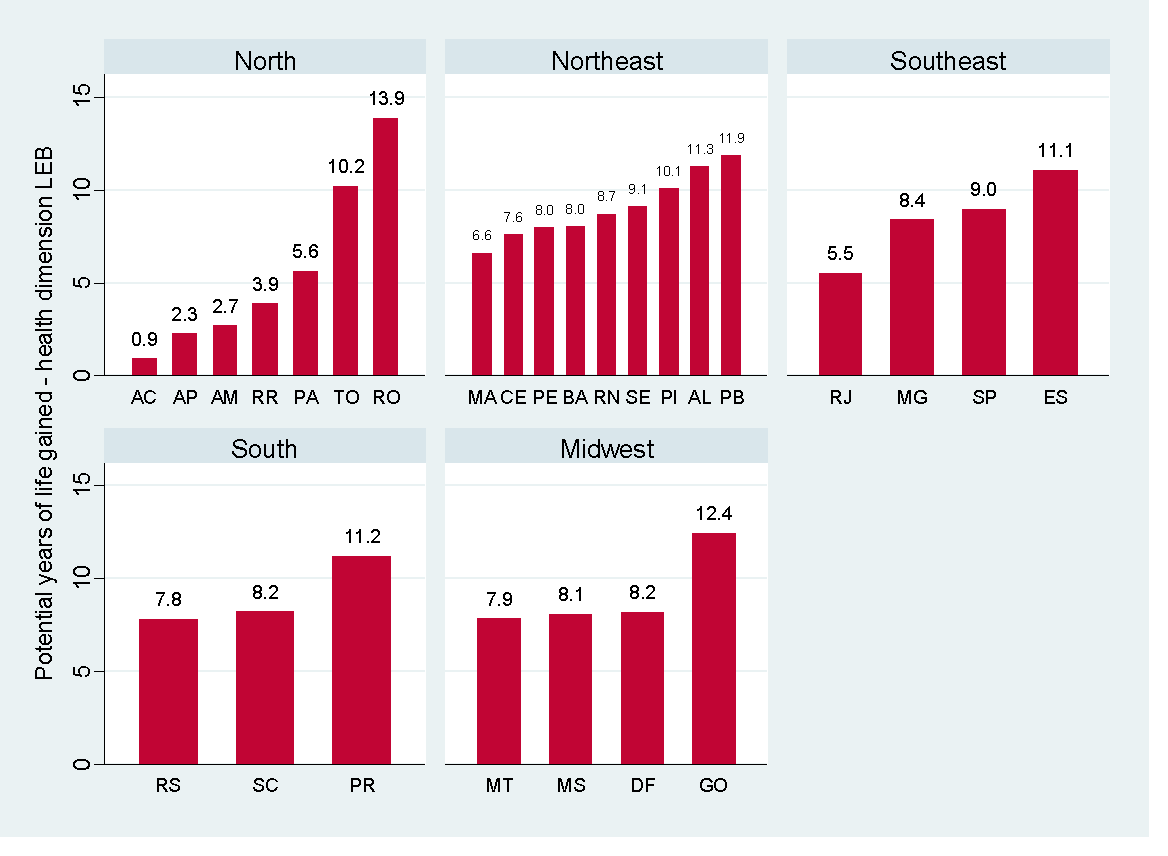


**Graph S5.10** - Potential years of life gained by increased efficiency in the health dimension by Federative Unit, 2010-19, Brazil.


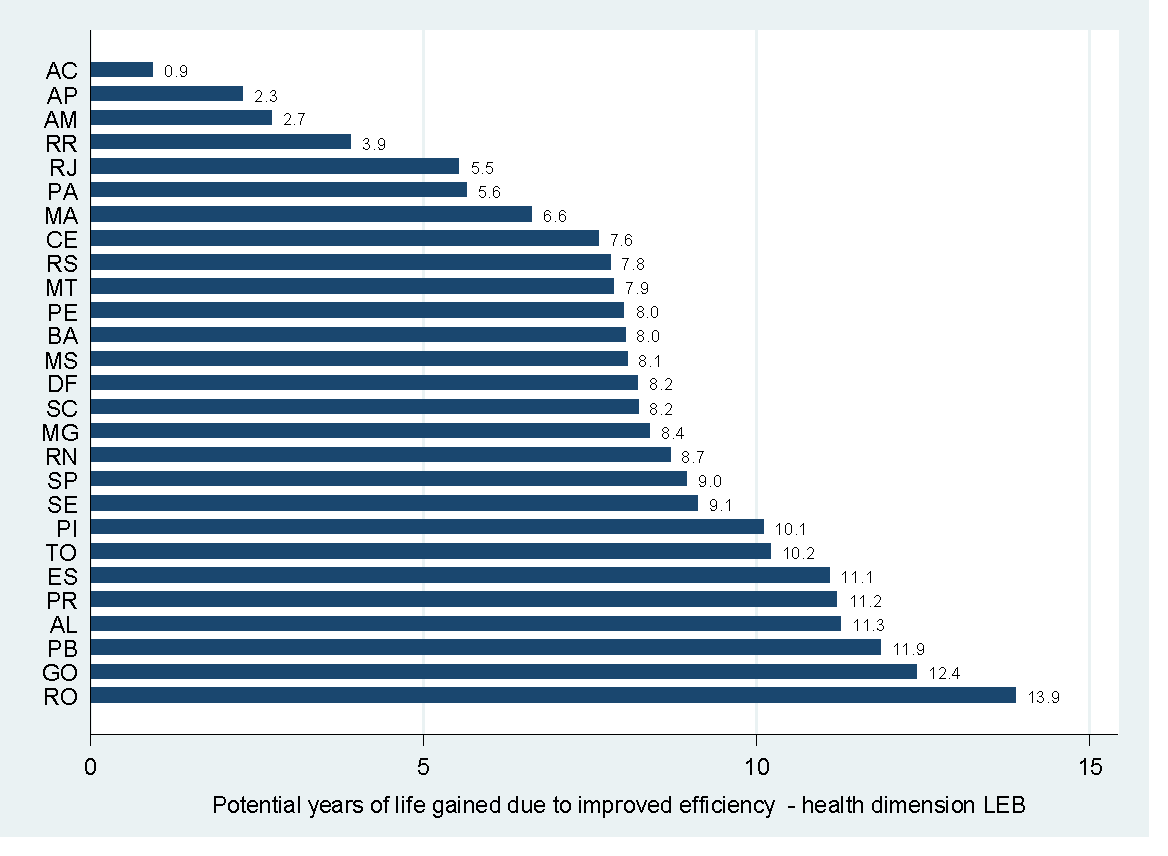


**Graph S5.11**- Potential reduction in the infant mortality rate due to increased efficiency in the health dimension by macro-region and Federative Unit, 2010-19, Brazil.


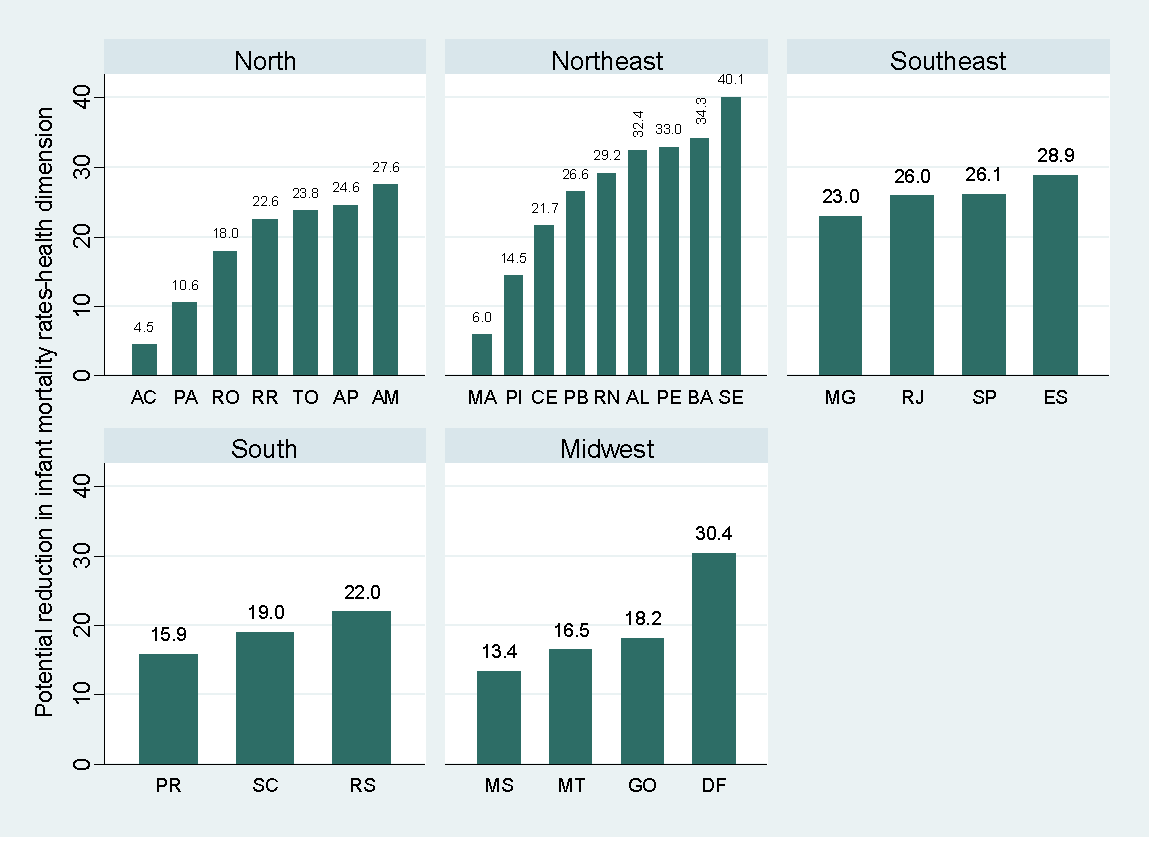


**Graph S5.12** - Potential reduction in the infant mortality rate due to increased efficiency in the health dimension by Federative Unit, 2010-19, Brazil.


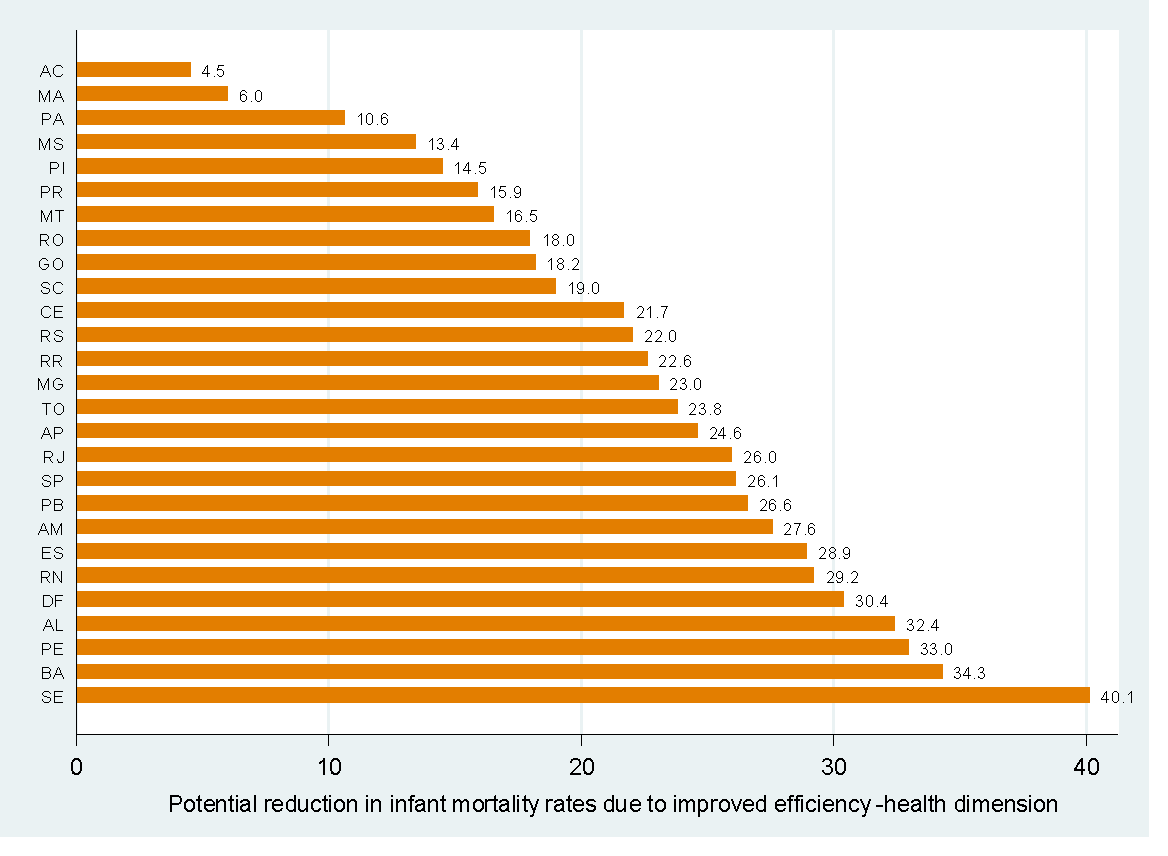


**Graph S5.13** - Potential years of life gained by increased efficiency in the intersectoral dimension by macro-region and Federative Unit, 2010-19, Brazil.


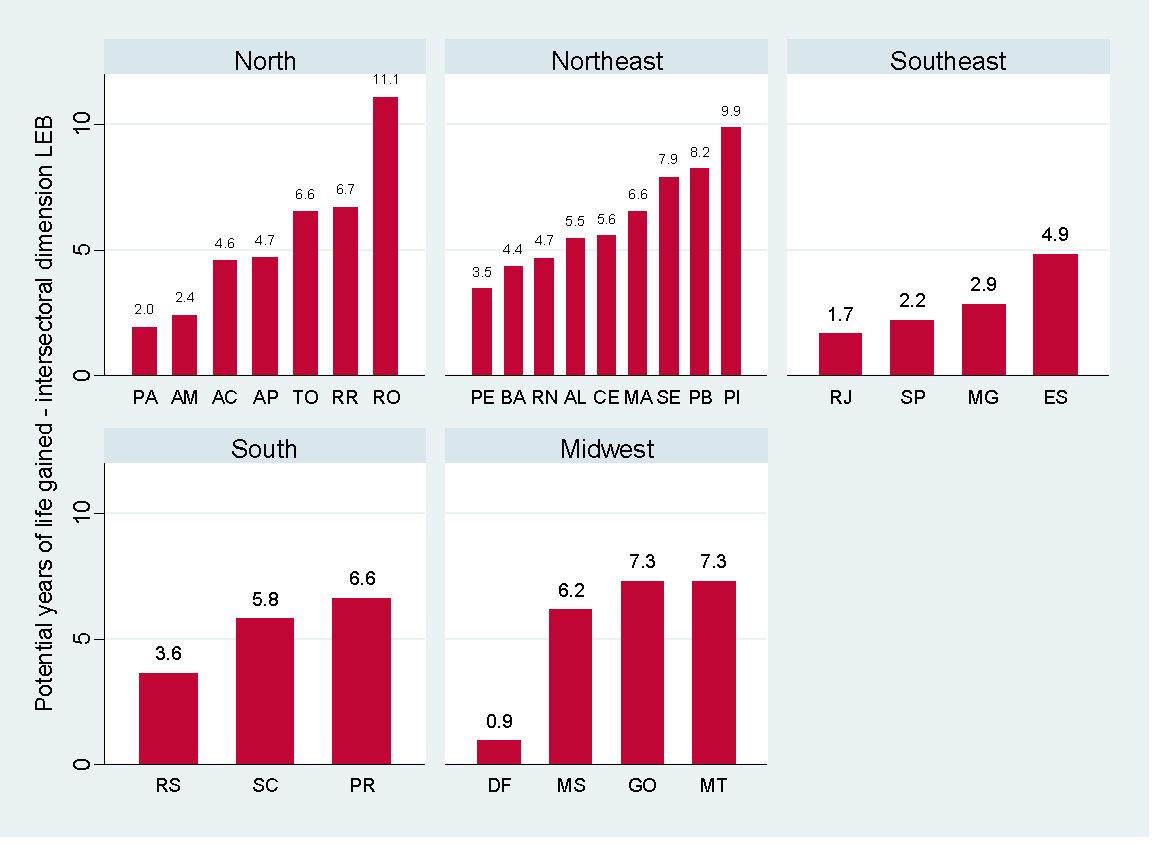


**Graph S5.14** - Potential years of life gained by increased efficiency in the intersectoral dimension by Federative Unit, 2010-19, Brazil.


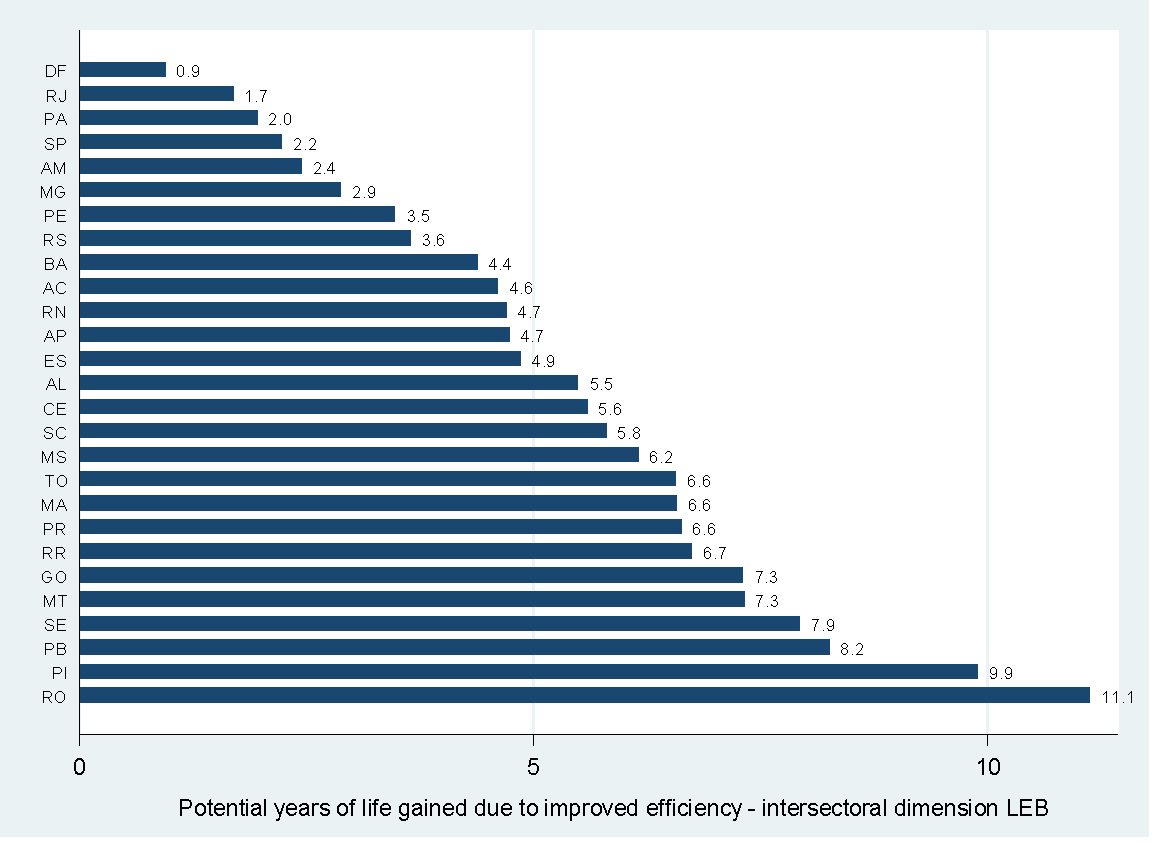


**Graph S5.15** - Potential reduction in infant mortality rate due to increased efficiency in the intersectoral dimension by macro-region and Federative Unit, 2010-19, Brazil.


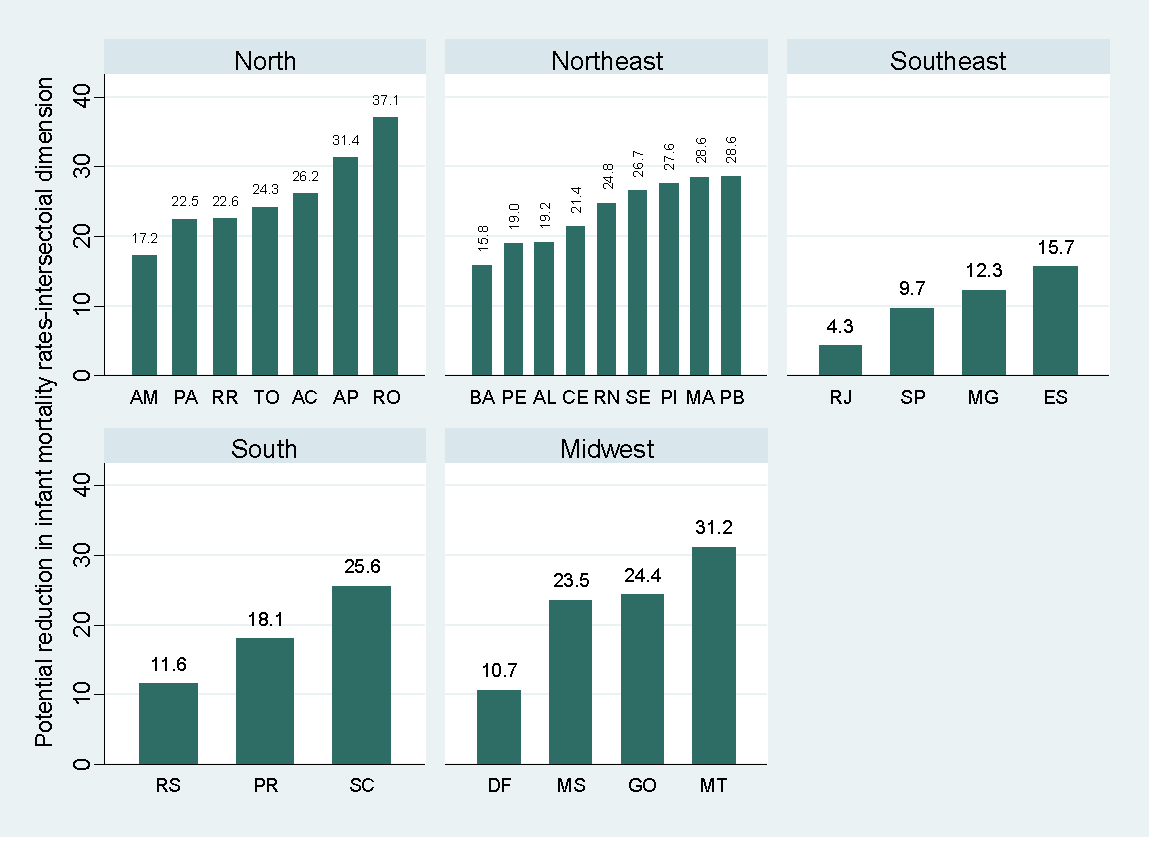


**Graph S5.16** - Potential reduction in the infant mortality rate due to increased efficiency in the intersectoral dimension by Federative Unit, 2010-19, Brazil.

**
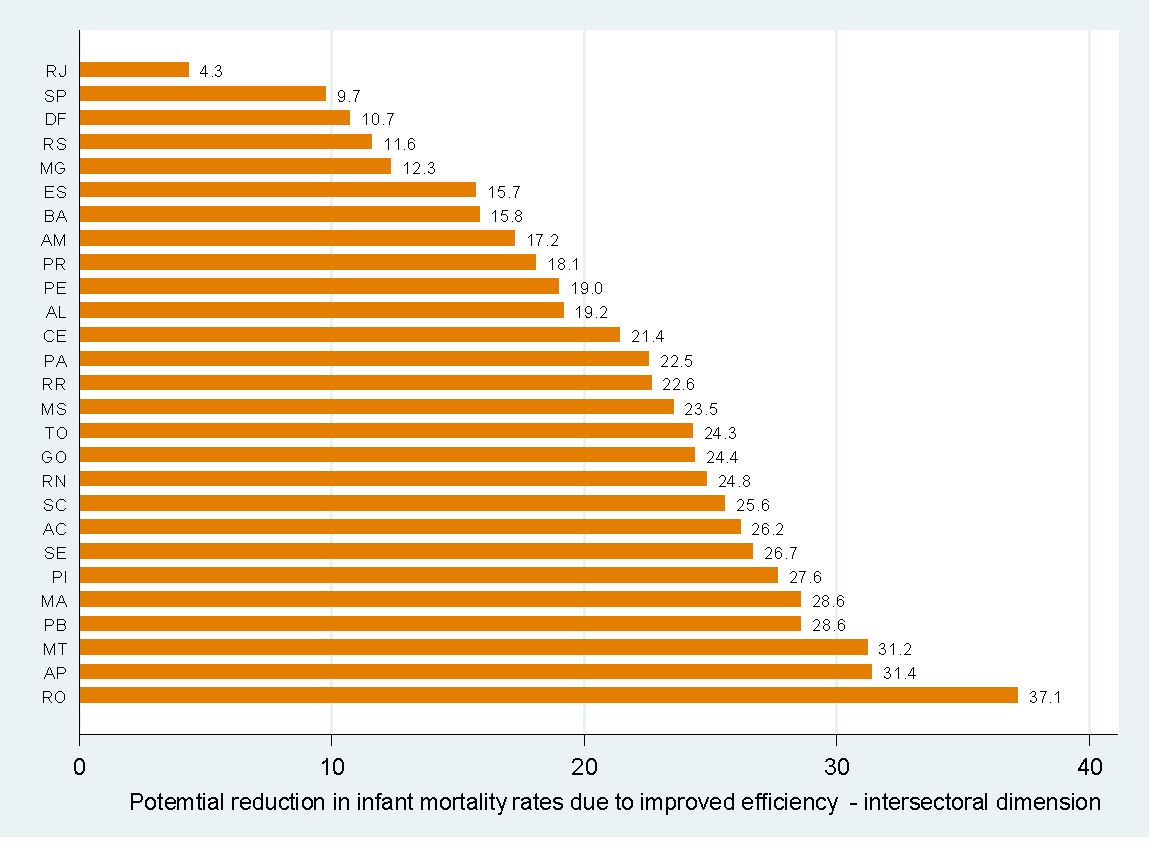
**

**More details about the Brazilian states**

Brazil has 27 Federative Units (26 states and 1 Federal District), distributed in five macroregions. The South and Northeast have the largest populations, whereas the North region has the largest territory, with low densities, especially in the Amazon region. Most health and economic indicators have better results in the South and Southeast, with intermediate results in the Midwest, but they also exhibit more inequalities and social disparities. Table S5.1, below presents a wide range of indicators of these states.

According to the graphs presented above (Graphs S5,1-S5.16), we have prepared a short summary below on the efficiency results in the Brazilian states.

Regarding Life Expectancy at birth, the states with the best results are in the southeast region (Rio de Janeiro-RJ and São Paulo-SP), besides Amazonas, Distrito Federal-DF (capital), Rio Grande do Sul-RS, and Pernambuco. As for infant mortality, the southeast region stands out, followed by Rio Grande do Sul and the capital (Federal District). The North and Northeast regions present worse results, and the rest of the Midwest and South regions are not much better.

Considering the resources dimension, some states in the Northeast stand out, mainly Maranhão, Piauí, and Alagoas, as well as Acre in the North. In the health dimension, the North stands out, especially Acre (both variables), Amapá (LEB), Amazonas (LEB), and Pará (IM). Maranhão, in the Northeast, also stands out in this dimension, especially for IM. In the intersectoral dimension, the most relevant in the overall analysis, the southeastern region (RJ and SP, more strongly) presents the best results, followed by the Federal District and Pará (LEB). It is worth mentioning that the level of transparency (Federal Public Ministry-MPF) was highly significant in the bivariate analysis in the expected sense, although it did not remain in the dimensional models. In contrast, income inequality by gender was statistically significant in the opposite direction and did not remain in the final models.

**Table S5.1- Social, health, demographic and economic indicators, Brazilian states, 2021-23.**

| States | Municipalities (n) | Population | Total area (Km^2^) | % urban areas | % urban  population | HDI | GDP per capital (R$) | Gini index | Life Expectancy  at biirth | Infant mortality | Competitiveness score |
| --- | --- | --- | --- | --- | --- | --- | --- | --- | --- | --- | --- |
| **North** |  |  |  |  |  |  |  |  |  |  |  |
| Acre | 22 | 830,018 | 164,173,429 | 0.13 | 72.61 | 0.710 | 23.569.31 | 0.539 | 72.26 | 17.19 | 25.6 |
| Amapá | 16 | 733,759 | 142,470,762 | 0.11 | 89.81 | 0.688 | 22.902.86 | 0.526 | 71.70 | 18.07 | 26.4 |
| Amazonas | 62 | 3,941,613 | 1,559,255,88 | 0.04 | 79.17 | 0.700 | 30.803.56 | 0.529 | 69.61 | 15.69 | 43.5 |
| Pará | 144 | 636,707 | 1,245,870,70 | 0.14 | 68.49 | 0.690 | 29.953.43 | 0.529 | 69.62 | 14.66 | 35.7 |
| Rondônia | 52 | 1,581,196 | 237,754,17 | 0.22 | 73.22 | 0.700 | 32.044.73 | 0.459 | 68.86 | 13.41 | 40.5 |
| Roraima | 15 | 830,018 | 164,173,43 | 0.10 | 76.41 | 0.699 | 27.887.57 | 0.596 | 69.71 | 18.79 | 27.3 |
| Tocantins | 139 | 8,120,131 | 277,423,63 | 0.20 | 78.81 | 0.731 | 32.214.73 | 0.513 | 71.74 | 12.33 | 42.3 |
| **Northeast** |  |  |  |  |  |  |  |  |  |  |  |
| Alagoas | 102 | 3,127,683 | 27,830,661 | 2.11 | 73.64 | 0.684 | 22.662.01 | 0.526 | 69.87 | 12.83 | 40.6 |
| Bahia | 417 | 14,141,626 | 564,760,43 | 0.50 | 72.07 | 0.691 | 23.530.94 | 0.546 | 71.34 | 15.35 | 34.1 |
| Ceará | 184 | 8,794,957 | 148,894,45 | 1.07 | 75.09 | 0.734 | 21.090.10 | 0.549 | 72.02 | 11.73 | 50.7 |
| Maranhão | 217 | 6,776,699 | 329,651,50 | 0.47 | 63.07 | 0.676 | 17.471.85 | 0.53 | 67.90 | 15.32 | 35.3 |
| Paraíba | 223 | 3,974,687 | 56,467,24 | 1.20 | 75.37 | 0.698 | 19.081.81 | 0.562 | 71.72 | 14.72 | 49.8 |
| Pernambuco | 185 | 9,058,931 | 98,067,88 | 1.44 | 80.15 | 0.719 | 22.823.59 | 0.579 | 72.83 | 13.27 | 41.0 |
| Piauí | 224 | 3,271,199 | 251,755,48 | 0.36 | 65.77 | 0.690 | 19.465.69 | 0.516 | 68.57 | 15.76 | 34.5 |
| Rio Grande do Norte | 167 | 3,302,729 | 52,809,60 | 1.54 | 77.82 | 0.728 | 22.516.97 | 0.587 | 74.16 | 11.06 | 34.2 |
| Sergipe | 75 | 2,210,004 | 21,938,19 | 2.11 | 73.51 | 0.702 | 22.177.45 | 0.568 | 70.85 | 17.63 | 37.7 |
| **Midwest** |  |  |  |  |  |  |  |  |  |  |  |
| Distrito Federal | 1 | 2,817,381 | 5,760,78 | 10.25 | 96.62 | 0.814 | 92.732.27 | 0.566 | 73.16 | \| 10.08 \| \| --- \| | 69.1 |
| Goiás | 246 | 7,056,495 | 340,242,86 | 0.59 | 90.29 | 0.737 | 37.414.08 | 0.467 | 68.28 | \| 12.66 \| \| --- \| | 61.7 |
| Mato Grosso | 141 | 3,658,649 | 903,208,36 | 0.14 | 81.90 | 0.736 | 65.426.10 | 0.461 | 68.77 | \| 14.08 \| \| --- \| | 61.5 |
| Mato Grosso do Sul | 77 | 2,757,013 | 357,142,08 | 0.24 | 85.64 | 0.742 | 50.086.07 | 0.496 | 70.03 | \| 12.35 \| \| --- \| | 60.3 |
| **Southeast** |  |  |  |  |  |  |  |  |  |  |  |
| Espírito Santo | 78 | 3,833,712 | 46,074,45 | 1.59 | 85.29 | 0.771 | 45.353.81 | 0.508 | 76.86 | \| 10.79 \| \| --- \| | 60.3 |
| Minas Gerais | 853 | 20,539,989 | 586,513,98 | 0.80 | 83.38 | 0.774 | 40.052.13 | 0.487 | 75.75 | 11.37 | 61.9 |
| Rio de Janeiro | 92 | 16,055,174 | 43,750,43 | 6.46 | 96.71 | 0.762 | 54.359.61 | 0.565 | 71.12 | \| 13.16 \| \| --- \| | 50.8 |
| São Paulo | 645 | 44,411,238 | 248,219,49 | 3.47 | 95.88 | 0.806 | 58.302.29 | 0.533 | 73.61 | \| 11.31 \| \| --- \| | 89.8 |
| **South** |  |  |  |  |  |  |  |  |  |  |  |
| Paraná | 399 | 11,444,380 | 199,298,98 | 1.68 | 85.31 |  | 47.421.76 | 0.475 | 72.11 | \| 10.32 \| \| --- \| | 72.1 |
| Santa Catarina | 295 | 7,610,361 | 95,730,69 | 2.64 | 83.99 | 0.792 | 58.400.55 | 0.424 | 74.6 | \| 9.79 \| \| --- \| | 84.2 |
| Rio Grande do Sul | 497 | 10,882,965 | 281,707,15 | 1.28 | 85.10 | 0.771 | 50.693.51 | 0.468 | 72.84 | \| 10.49 \| \| --- \| | 64.2 |

**Notes:** GDP- 2023 (values 2021) Btazilian Institute of Geography and Statistics-IBGE; HDI-Human Development Index, 2021 United Nations Development Programme-UNDP; Population-Census 2022; Gini index-IBGE PNAD 2016; Life expectancy-PNAD 2021; Infant mortality, Datasus, 2022.

Competitiveness scores from Public Leadership Center, 2023 dimensions and weights: Public Security (12.7); Infrastructure (12.4); Social Sustainability (11.6); Fiscal solidity (11.5); Education (11.5); Environmental Sustainability (8.3); State administrative efficiency (9.2); Human capital (8.2); Market potential (7.8); Innovation (6.8).
