# Supplementary material for: Intersectional equity in Brazil’s remote rural municipalities: the road to efficiency and effectiveness in local health systems
Source: Front Public Health. 2024 Sep 10;12:1401193. doi: 10.3389/fpubh.2024.1401193 (PMC11419982; doi:10.3389/fpubh.2024.1401193)
Supplement: Supplementary file 6 [file Table_6.DOCX]

**Supplement 6-Bivariate analysis by dimension - Brazilian states with RRL only**

**Chart S6.1** - Bivariate analyses according to resource dimension, for selected effect variables. Federative units with remote rural municipalities. Brazilian states with RRL, 2010-19.

| **Variable/coefficients(beta)** | **Life Expectancy at birthB** | **Infant Mortality** | **Probability of deaths from preventable causes** | **Low birth weight(%)** | **Adolescent mothers (%)** |
| --- | --- | --- | --- | --- | --- |
| Average income | 0,03 | -0,08 | 0,01 e-1 | 2,86 e-5 | -2,69 e-4 |
| GDP - % agriculture | -23,51 | 72,38 | -1,81 | -0,03 | 0,19 |
| GDP - % industry | -5,02 | 12,45 | 0,50 | 0,02 e-1 | 0,05 |
| GDP - % servicess | 45,94 | -135,18 | 2,65 | 0,05 | -0,39 |
| GDP - % Public Administration | 7,38 | -22,78 | -0,21 | 0,02 e-1 | -0,05 |
| GDP per capita | 0,02 e-2 | -4,14 e-4 | 9,16 e-6 | 1,09 e-7 | -1,58 e-6 |
| TIG (Intergovernmental transfers) | 0,11 | -0,33 | 0,06 e-1 | 2,11 e-4 | -1,57 e-3 |
| DPC (local health expenditure per capita) | 0,01 | -0,03 | 0,05 e-2 | 7,45 e-6 | -8,79 e-5 |
| RPC (local health revenue per capita) | 0,02 | -0,05 | 0,09 e-2 | 9,21 e-6 | -1,65 e-4 |
| PHC expenditure | -0,04 e-1 | 0,01 | -0,03 e-2 | 3,14 e-5 | 1,35 e-4 |
| INV (investments) | 0,01 | -0,03 | 0,05 e-1 | 3,03 e-5 | -2,68 e-4 |
| DPES (personnel costs) | 1,03 e-7 | -2,78 e-7 | 2,60 e-9 | 7,88 e-11 | -9,57 e-10 |
| Coverage FHS | 5,54 | -17,39 | 0,47 | 4,99 e-3 | -0,05 |
| Coverage PHC | 6,44 | -20,10 | 0,48 | 2,73 e-3 | -0,05 |
| Coverage CHW | -4,06 | 10,90 | 0,13 | -1,45 e-2 | 0,05 |
| Density area PHC facilities | 74,26 | -216,18 | 3,96 | 0,06 | -0,68 |
| Density pop PHC facilities | 4406,97 | -14523,81 | 486,76 | 12,42 | -31,90 |
| Density area FHS | 69,38 | -192,36 | 1,37 | 0,05 | -0,59 |
| Density area CHW | -238,94 | 632,42 | -11,21 | -0,50 | 1,50 |
| Density area NASF | 430,28 | -1223,16 | 19,51 | 0,40 | -4,10 |
| Density pop NASF | 18820,51 | -56077,93 | 813,94 | 12,35 | -180,93 |
| Proportion of PHC facilities | -15,98 | 46,52 | -1,16 | 2,37 e-3 | 0,16 |
| Proportion of Specialized care facilities | 21,93 | -65,60 | 1,59 | 0,02 | -0,19 |
| Density total teams pop | 12751,82 | -38893,09 | 1073,89 | 2,20 | -121,04 |
| Density total facilities pop | 5353,30 | -14850,75 | 356,02 | 2,30 | -55,50 |
| Density total equipment pop | 19644,87 | -53632,45 | 1571,20 | 24,21 | -197,93 |
| Density of PHC doctors pop | 3640,13 | -9419,12 | 240,23 | 0,89 | -53,07 |
| Density of doctors pop | 3667,54 | -9474,04 | 209,87 | 3,14 | -50,55 |
| Density of SUS doctors pop | 3059,79 | -8107,04 | 212,16 | 2,77 | -43,88 |
| Density of nurses pop | 7041,86 | -19946,09 | 384,29 | 2,55 e-4 | -66,08 |
| % of surgical bedss | -6,26 | 15,84 | -0,07 | -0,03 | 0,05 |
| Density of beds pop | -766,11 | 2198,76 | 84,75 | -0,60 | 6,19 |
| Density of beds area | 0,92 | -2,45 | -0,04 e-1 | 1,20 e-3 | -4,59 e-3 |
| % surgeonss | -1,61 | 6,82 | 0,76 | 6,11 e-3 | -0,09 |
| % PHC doctors | -6,93 | 19,82 | -0,60 | -0,02 | 0,07 |
| % of SUS doctors | -12,87 | 34,53 | -0,69 | -0,02 | 0,14 |
| % SUS PHC doctors | 1,06 | -2,23 | -0,01 | 1,41 e-4 | -0,02 |
| % of surveillance units | 4,63 | -16,45 | 0,22 | 1,87 e-3 | -0,10 |
| % of fluvial units | 49,86 | -198,76 | 6,21 | 9,68 e-3 | -0,20 |
| % of obstetric beds | -15,09 | 42,22 | -0,19 | -0,01 | 0,18 |
| % of FHS-OH teams | -7,63 | 23,77 | -0,21 | -0,01 | 0,06 |
| % of CHW teams | -9,62 | 28,13 | -0,99 | -0,02 | 0,08 |
| % of Riverine and/or Fluvial teams | 14,61 | -58,32 | 1,32 | 0,01 | -0,06 |
| % PHS teams | 25,67 | -74,66 | 0,64 | 4,88 e-3 | -0,08 |
| % of private health plans beneficiaries | -8,97 | 16,85 | 1,16 | -6,01 e-3 | 0,14 |
| % of diagnostic and therapeutic units | 10,49 | -33,32 | 0,90 | 0,01 | -0,07 |
| **Variable/coefficients(beta)** | **Life Expectancy at birthB** | **Infant Mortality** | **Probability of deaths from preventable causes** | **Low birth weight(%)** | **Adolescent mothers (%)** |

**Note:** In green, results associated with improvement; in red, with worsening; in black, not significant.

**Chart S6.2** - Bivariate analyses according to health dimension, for selected effect variables. Federative units with remote rural municipalities. Brazilian states with RRL, 2010-19.

| **Variable/coefficients(beta)** | **Life Expectancy at birthB** | | **Infant Mortality** | **Probability of deaths from preventable causes** | **Low birth weight(%)** | **Adolescent mothers (%)** |
| --- | --- | --- | --- | --- | --- | --- |
| Deaths in children <1y-total | 0,09 | -0,23 | | 0,05 e-1 | -9,11 e-5 | -6,32 e-4 |
| Total deaths – families | 1,78 | -4,48 | | 0,11 | -2,54 e-3 | -0,01 |
| Total hospitalizations – families | -173,89 | 504,89 | | -9,44 | -0,26 | 1,47 |
| Home visits – familes | -2,71 | 7,70 | | -0,20 | -9,85 e-3 | 0,03 |
| % pregnant adolescent women | -5,01 | 13,89 | | -0,60 | 8,24 e-3 | --- |
| % of prenatal started in the first trimester | 14,71 | -45,45 | | 1,02 | 1,66 e-2 | -0,11 |
| Exclusive breastfeeding | 1,53 | -5,22 | | 0,32 | -0,01 | -0,02 |
| % of up to date vaccination in<1y | 20,12 | -62,11 | | 1,62 | 9,88 e-3 | -0,09 |
| % of malnutrition in <1y | -11,06 | 35,90 | | 0,07 | -0,01 | 0,03 |
| % of acute respiratory infections and diarrhea in children <2y | -21,79 | 65,74 | | -1,31 | -0,02 | 0,16 |
| % of DM in families | 90,74 | -265,41 | | 4,28 | 0,04 | -0,83 |
| % of monitored DM | -14.60 | 41,75 | | -1,34 | -0,04 | 0,13 |
| % SAH in families | 0,09 | -0,27 | | 0,02 | 1,20 e-3 | 3,16 e-4 |
| % SAH nonitored | -7,85 | 21,81 | | -0,78 | -0,03 | 0,09 |
| %TB in families | -161,24 | 426,80 | | -5,91 | 0,58 | 1,55 |
| %Hansen disease in families | -64,98 | 257,86 | | 4,19 | 0,51 | 1,08 |
| Prenatal appointments | 16,35 | -49,79 | | 0,96 | 0,02 | -0,13 |
| Adolescent mothers (%) | -39,36 | 112,53 | | -2,14 | -0,03 | --- |
| Low birth weight (%) | 15,46 | -45,64 | | 2,20 | --- | --- |

**Note:** In green, results associated with improvement; in red, with worsening; in black, not significant.

**Chart S6.3** - Bivariate analyses according to the intersectoral dimension, for selected effect variables. Federative units with remote rural municipalities. Brazilian states with RRL, 2010-19.

| **Variable/coefficients(beta)** | **Life Expectancy at birthB** | | **Infant Mortality** | **Probability of deaths from preventable causes** | **Low birth weight(%)** | **Adolescent mothers (%)** |
| --- | --- | --- | --- | --- | --- | --- |
| % of highly educated black and brown men | 0,32 | -0,92 | | 0,02 | 3,42 e-4 | -2,79 e-3 |
| % of highly educated black and brown women | 0,26 | -0,76 | | 0,01 | 2,65 e-4 | -2,72 e-3 |
| % of highly educated white women | 0,27 | -0,80 | | 0,01 | 2,85 e-4 | -2,41 e-3 |
| % of highly educated white men | 0,31 | -0,90 | | 0,02 | 3,13 e-4 | -2,75e-3 |
| % of highly educated women | 0,29 | -0,85 | | 0,01 | 2,99 e-4 | -2,52 e-3 |
| % of highly educated men | 0,35 | -1,01 | | 0,02 | 3,77 e-4 | -3,02 e-3 |
| % of highly educated white people | 0,31 | -0,89 | | 0,02 | 3,18 e-4 | -2,70 e-3 |
| % of highly educated black and brown people | 0,29 | -0,85 | | 0,01 | 3,06 e-4 | -2,56 e-3 |
| % of low-income among white people | -0,25 | 0,76 | | -0,01 | -2,73 e-4 | 2,14 e-3 |
| % of low income among black and brown people | -0.23 | 0,68 | | -0,01 | -2,20 e-4 | 1,99 e-3 |
| % of unemployed white people | -0.38 | 1,07 | | -0,02 | -2,57 e-4 | 3,98 e-3 |
| % of unemployed black and brown people | -0,35 | 1,00 | | -0,02 | -2,06 e-4 | 3,65 e-3 |
| Average income among black and brown people | 0,03 | -0,09 | | 1,52 e-3 | 0,30 e-4 | 3,16 e-4 |
| Average income among white people | 0,02 | -0,04 | | 6,86 e-4 | 1,33 e-5 | 1,47 e-4 |
| Inequality of high education among women, according to ethnicity | -3,29 | 9,72 | | -0,20 | -3,90 e-3 | 0,03 |
| Inequality of high education among men, according to ethnicity | -2,89 | 8,55 | | -0,18 | -4,41e-3 | 0,02 |
| Inequality of high education , according to gender | -0,82 | 2,64 | | -0,07 | 3,79 e-4 | 0,01 |
| Inequality of high education, according to gender and ethnicity | -2,31 | 6,91 | | -0,15 | -3,79 e-3 | 0,02 |
| Municipal HDI | 35,02 | -103,46 | | 1,81 | 0,04 | -0,29 |
| MHDI-income | 59,85 | -176,73 | | 2,78 | 0,05 | -0,46 |
| MHDI-education | 23,56 | -69,26 | | 1,21 | 0,03 | -0,20 |
| MHDI-longevity | 65,35 | -193,51 | | 3,37 | 0,06 | -0,55 |
| Fertility rate | -5.00 | 15,18 | | -0,28 | -5,44 e-3 | 0,04 |
| Ageing rate | 2,33 | -6,75 | | 0,14 | 1,87 e-3 | -0,02 |
| % highly educated | 0,32 | -0,93 | | 0,02 | 3,38 e-4 | -2,78 e-3 |
| Gin Indexi | -30,24 | 86,86 | | -1,11 | -0,02 | 0,32 |
| Income quintile ratio (1ºand 5º) | -0,09 e-2 | 2,95 e-3 | | -0,01 e-3 | -1,53 e-6 | 9,50 e-6 |
| Illiteracy rates | -0,69 | 2,08 | | -0,04 | -6,79 e-4 | 5,50 e-3 |
| Unemployment rates | -0,40 | 1,13 | | -0,02 | -3,11 e-4 | 4,11 e-3 |
| Cleaning service garbage collection | 0,17 | -0,50 | | 0,08 e-1 | 9,77 e-5 | -1,46 e-3 |
| Waste collection service | 0,11 | -0,32 | | 0,09 e-1 | 2,97 e-4 | -8,19 e-4 |
| General sewage or rainwater service | 0,30 | -0,87 | | 0,02 | 2,73 e-4 | -2,99 e-3 |
| Septic tanks | 0,05 | -0,15 | | -0,01 e-1 | -4,71 e-5 | -4,06 e-4 |
| General water supply network | 0,25 | -0,74 | | 0,01 | 3,06 e-4 | -1,86 e-3 |
| Well or spring | 0,10 | -0,31 | | 0,06 e-1 | 1,43 e-4 | -4,68 e-4 |
| Bolsa Família cash transfer – basic benefit | -53,90 | 141,94 | | 2,11 | -0,02 | 0,48 |
| Bolsa Família cash transfer – variable benefit | -87,84 | 257,44 | | -2,10 | -0,09 | 0,74 |
| Bolsa Família cash transfer – variable youth benefit | -534,71 | 1562,55 | | -16,28 | -0,49 | 4,90 |
| Bolsa Família cash transfer – variable nursing mother benefit | 1778,59 | -5498,21 | | 157,22 | 2,09 | -13,98 |
| Bolsa Família cash transfer – variable pregnant women benefit | 1414,93 | -4331,69 | | 100,29 | 1,71 | -10,99 |
| Bolsa Família cash transfer – poverty erradication benefit | 38,49 | -125,88 | | 5,27 | 0,03 | -0,41 |
| **Variable/coefficients(beta)** | **Life Expectancy at birthB** | | **Infant Mortality** | **Probability of deaths from preventable causes** | **Low birth weight(%)** | **Adolescent mothers (%)** |

**Note:** In green, results associated with improvement; in red, with worsening; in black, not significant.
