# Supplementary material for: Intersectional equity in Brazil’s remote rural municipalities: the road to efficiency and effectiveness in local health systems
Source: Front Public Health. 2024 Sep 10;12:1401193. doi: 10.3389/fpubh.2024.1401193 (PMC11419982; doi:10.3389/fpubh.2024.1401193)
Supplement: Supplementary file 7 [file Table_7.DOCX]

**Supplement 7 – Graphs by rural-urban typologies for Brazilian states with RRL only. Ranking of the Brazilian states, according to rural-urban typology**

**Graph S7.1** - Distribution of potential years of life gained attributed to improved efficiency, according to rural-urban typologies. Brazilian states with RRL, 2010-19.


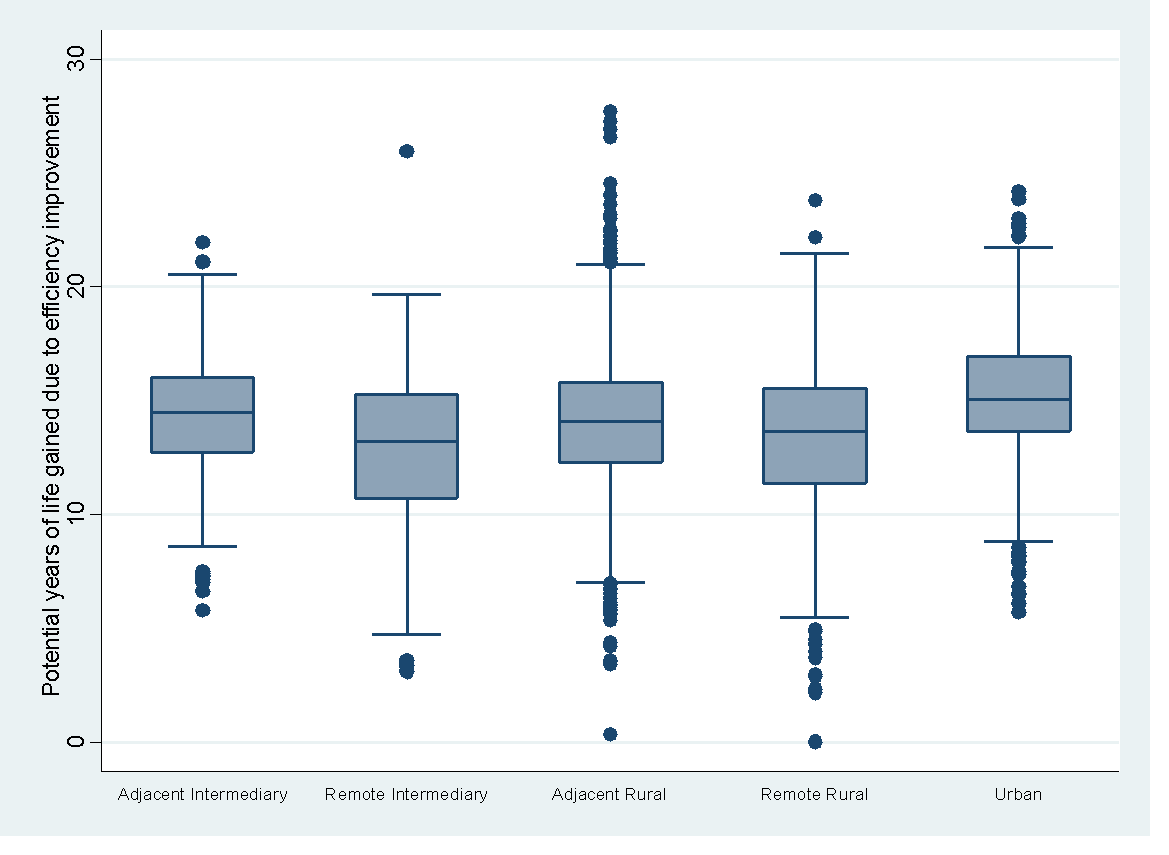


**Graph S7.2** - Potential years of life gained attributed to improved efficiency, according to rural-urban typologies and Federative Units


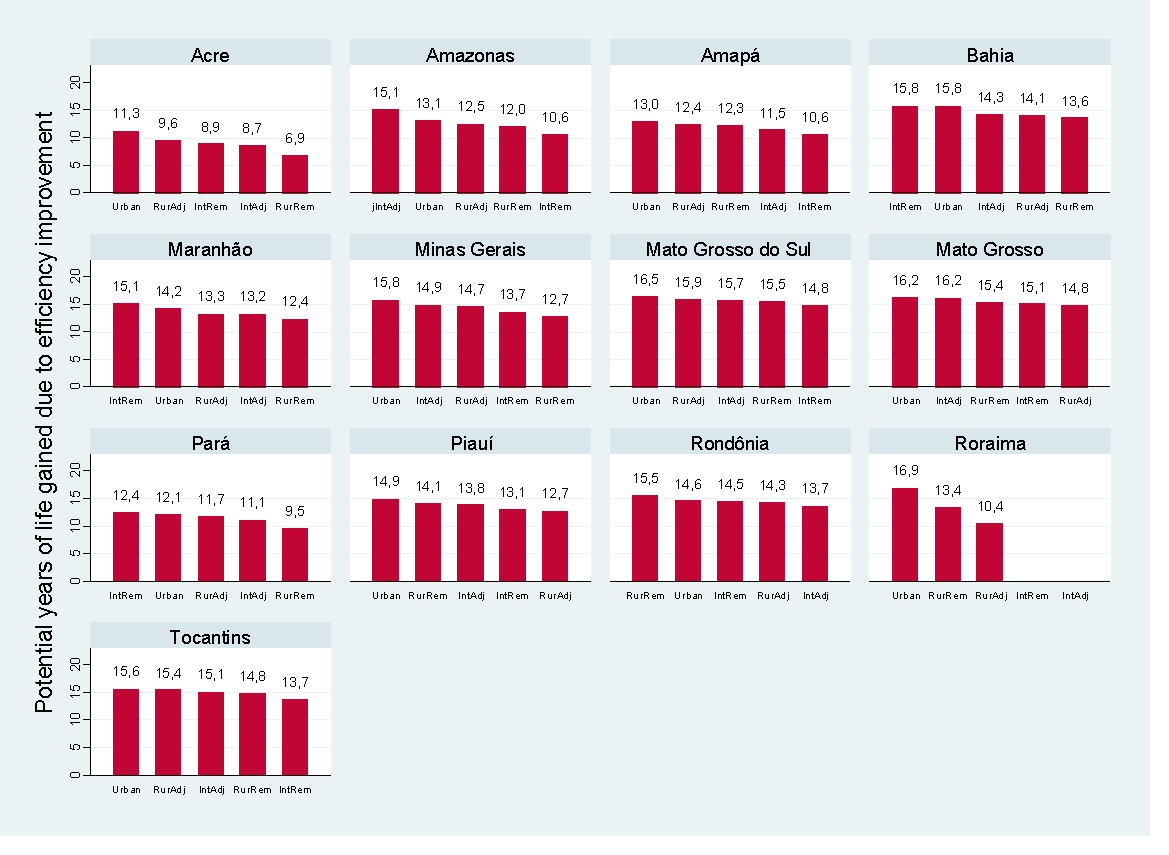


**Graph S7.3** - Distribution of the reduction in infant mortality rate attributed to improved efficiency, according to rural-urban typologies.


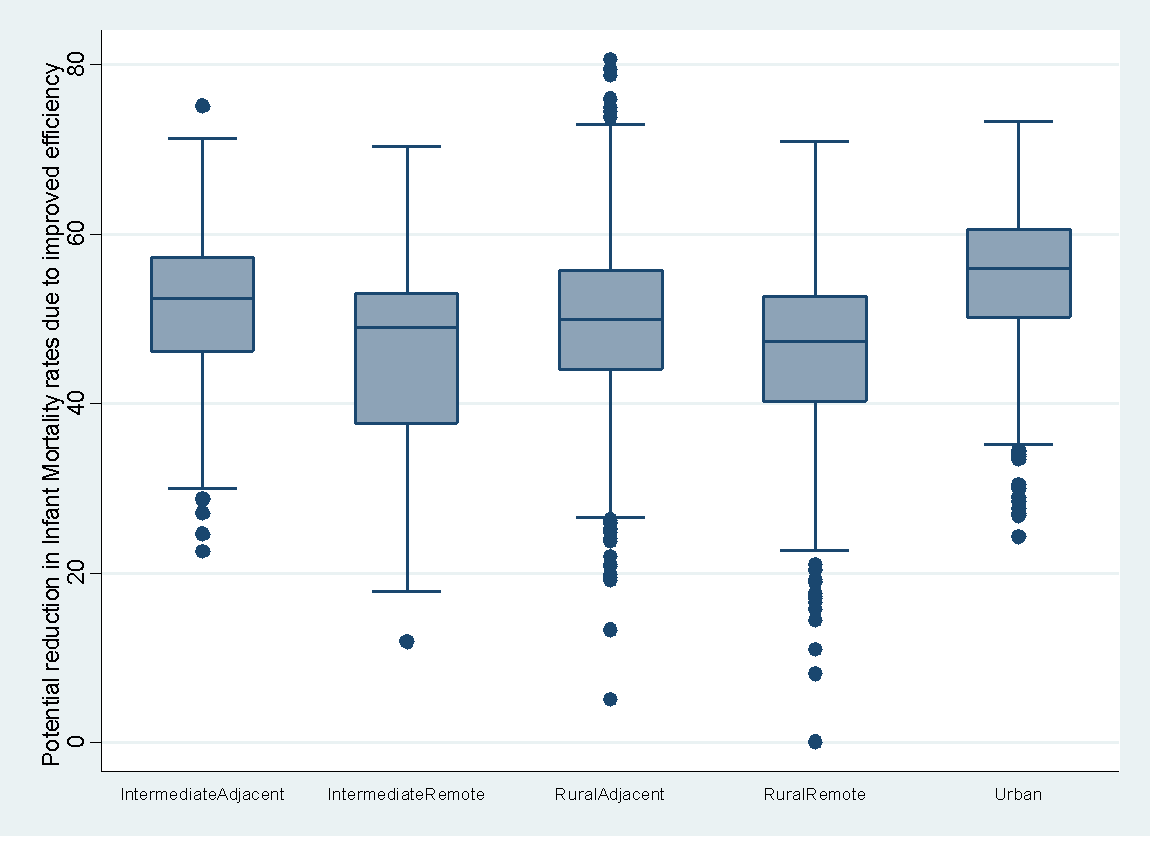


**Graph S7.4** - Reduction in the mortality rate attributed to improved efficiency, according to rural-urban typologies and Federative Units


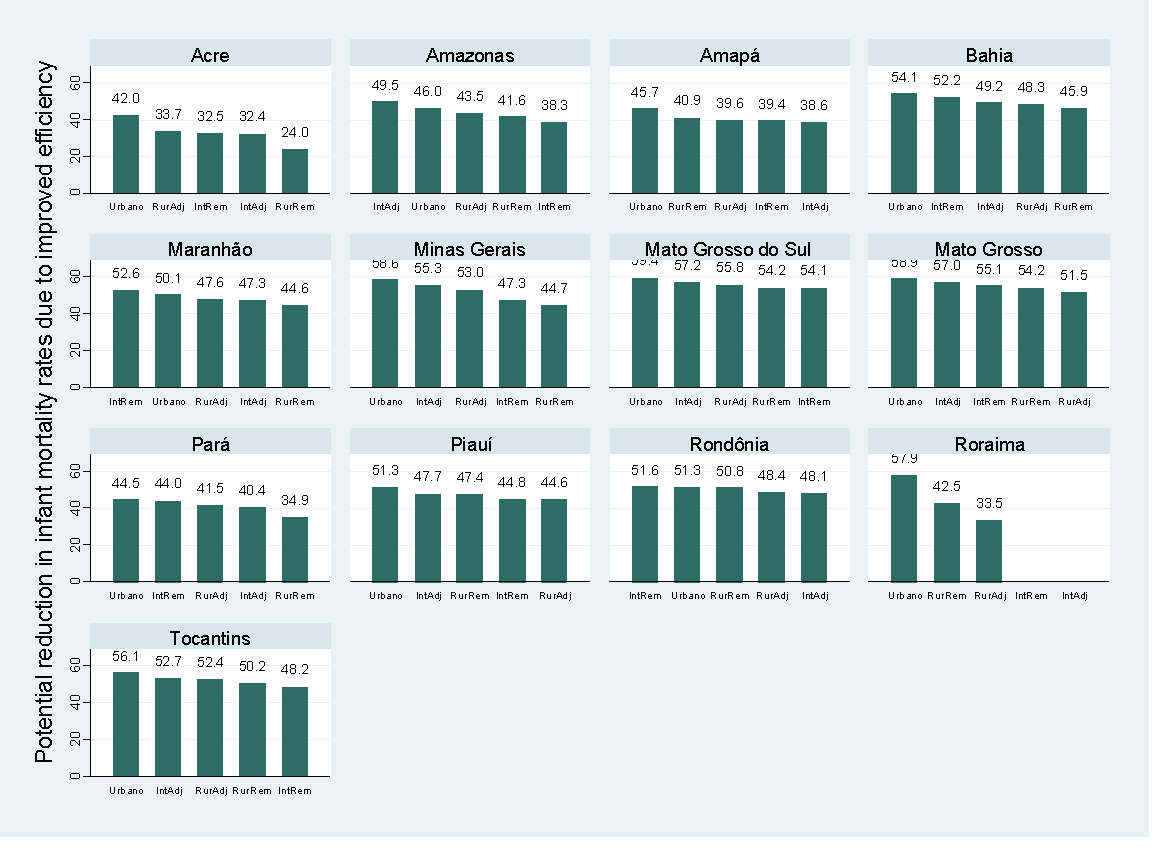


**Graph S7.5** - Distribution of the reduction in deaths from avoidable causes attributed to improved efficiency, according to rural-urban typologies


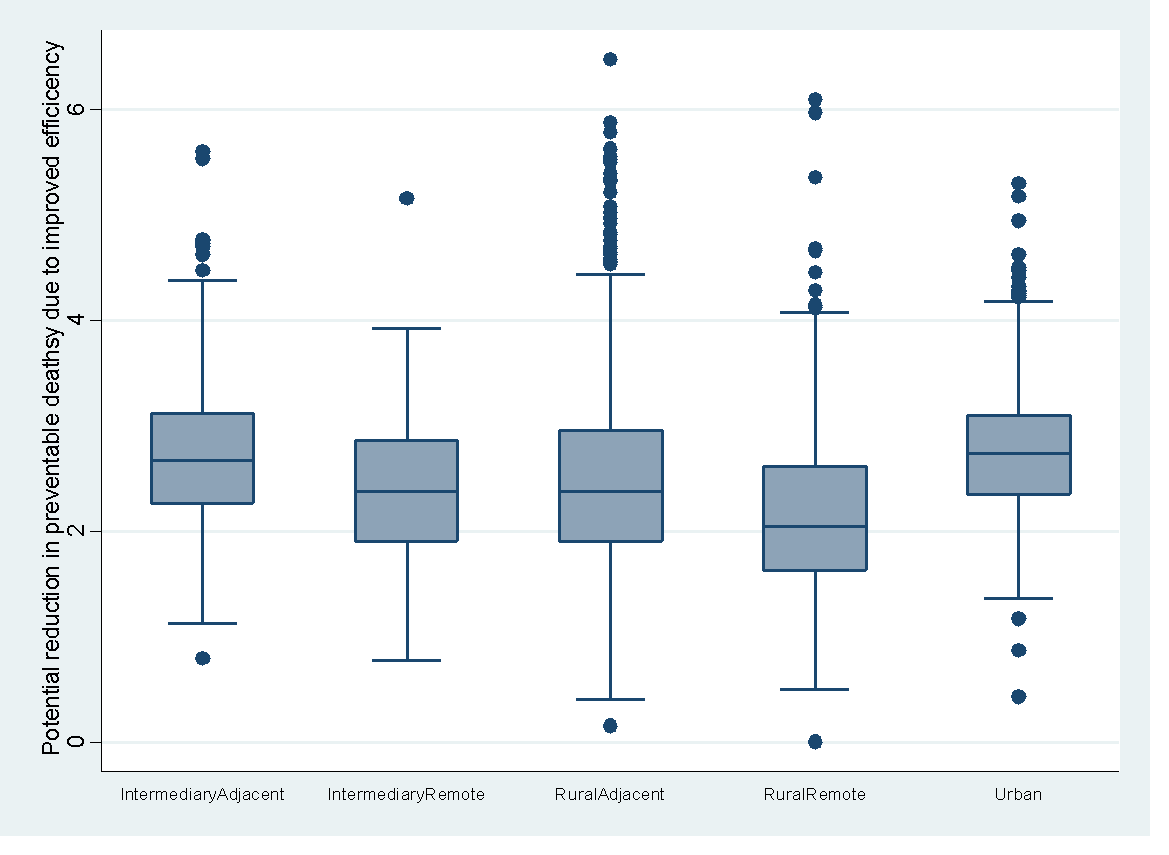


**Graph S7.6** Reduction in deaths from preventable causes attributed to improved efficiency, according to rural-urban typologies and Federative Units


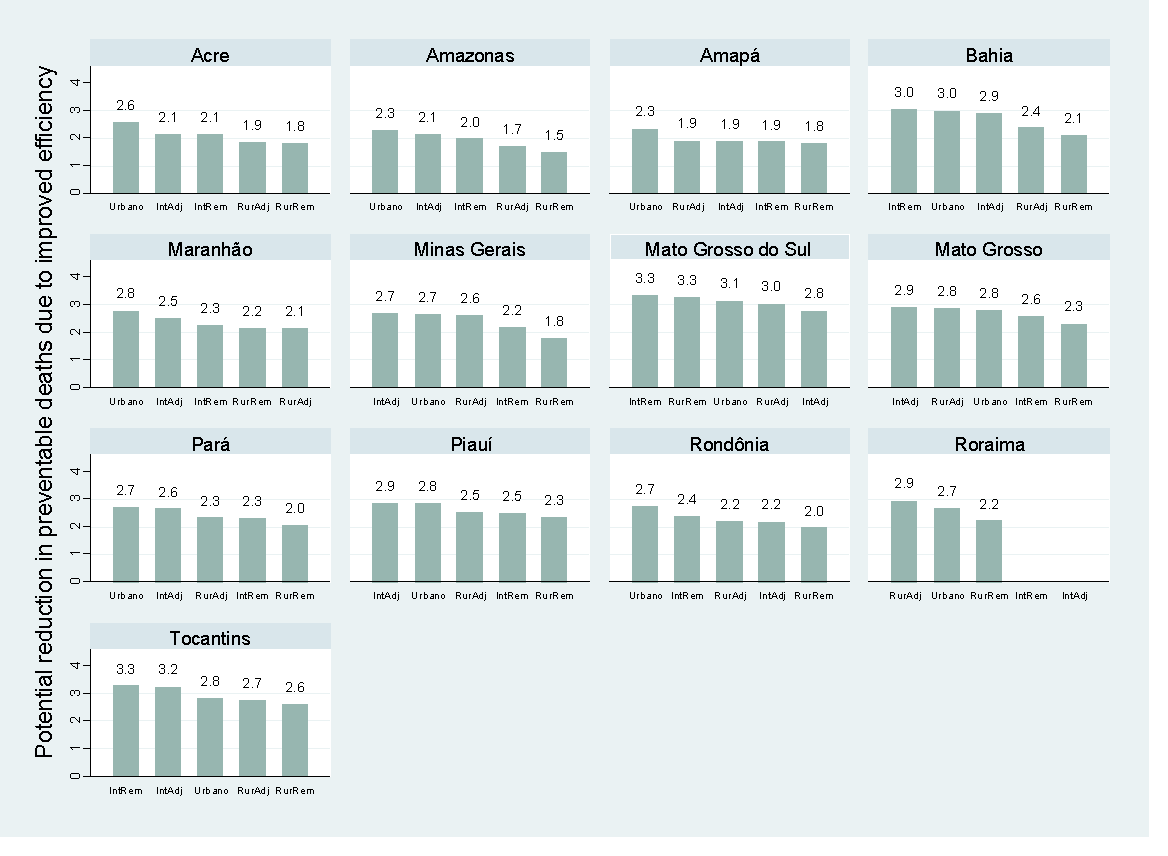


**Graph S7.7** - Distribution of the reduction in the % of newborns with low birth weight attributed to improved efficiency, according to rural-urban typologies.


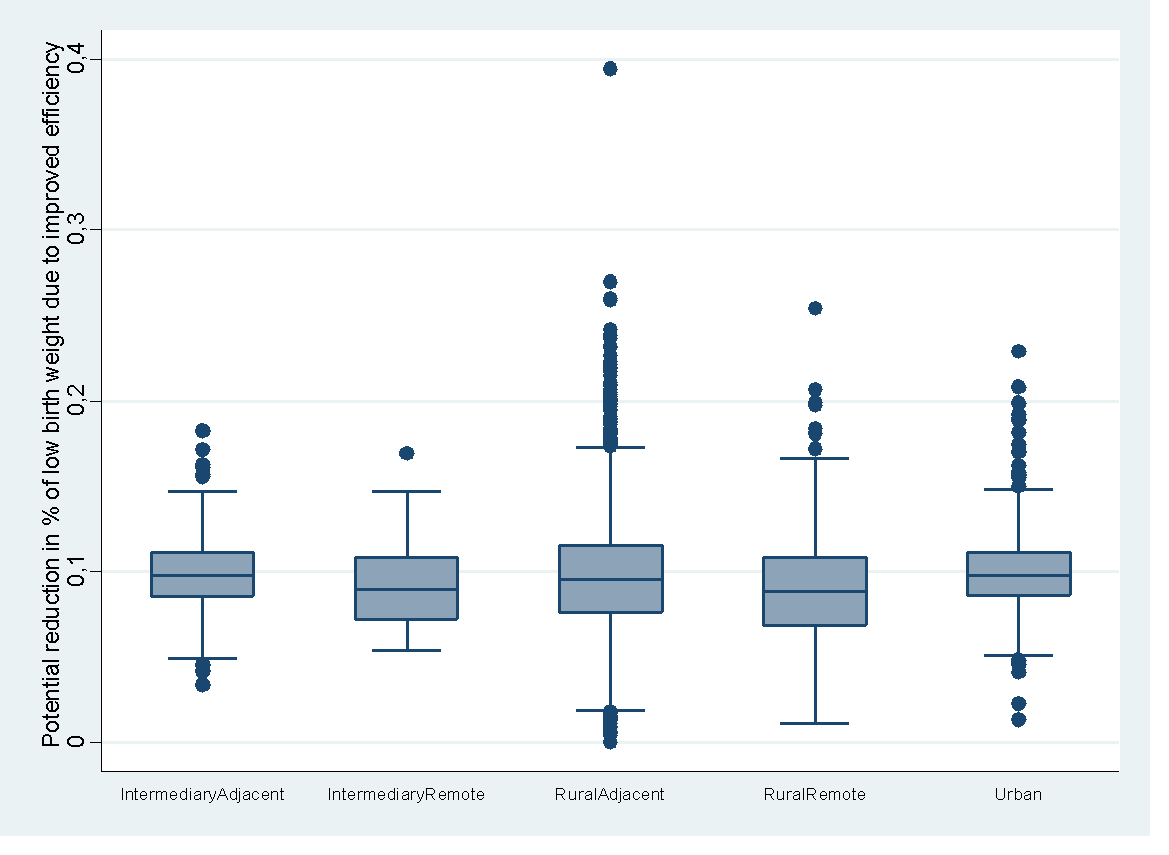


**Graph S7.8** Reduction in the % of newborns with low birth weight attributed to improved efficiency, according to rural-urban typologies and Federative Units


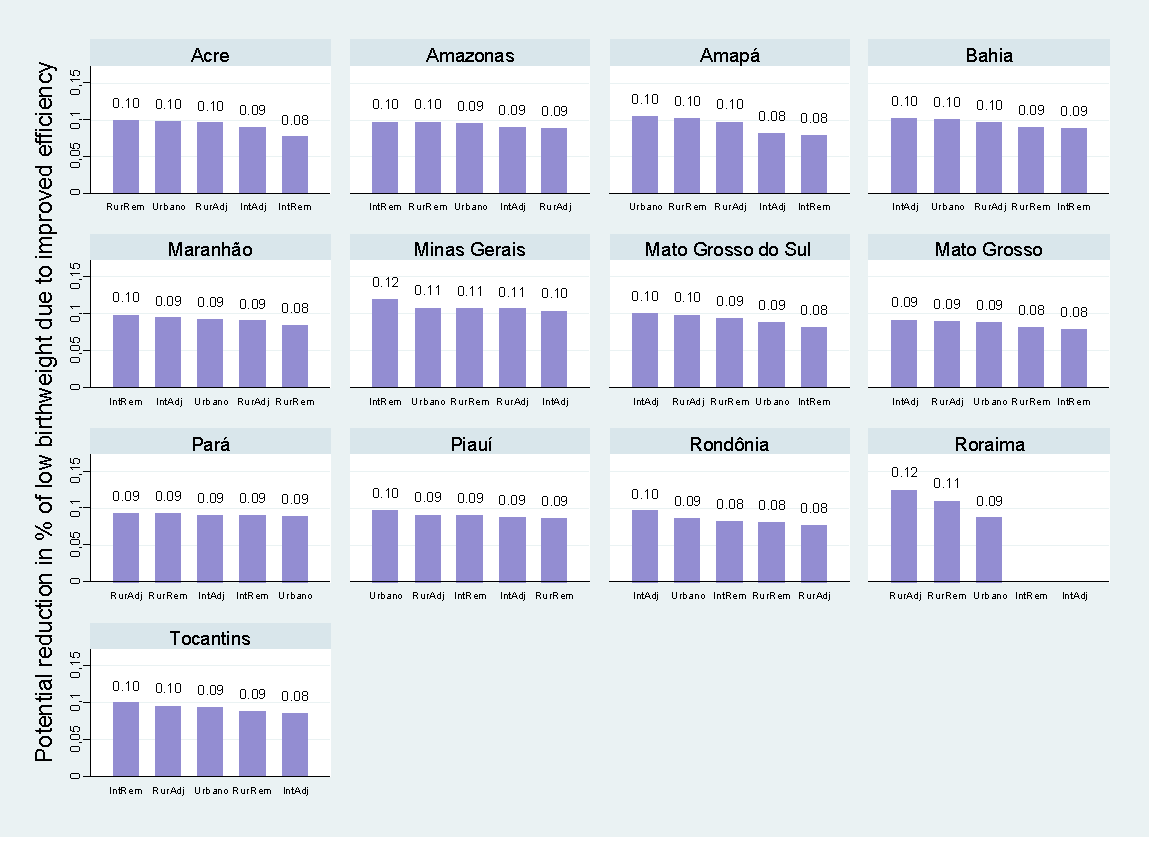


**Graph S7.9** - Distribution of the reduction in the proportion of teenage mothers attributed to improved efficiency, according to rural-urban typologies


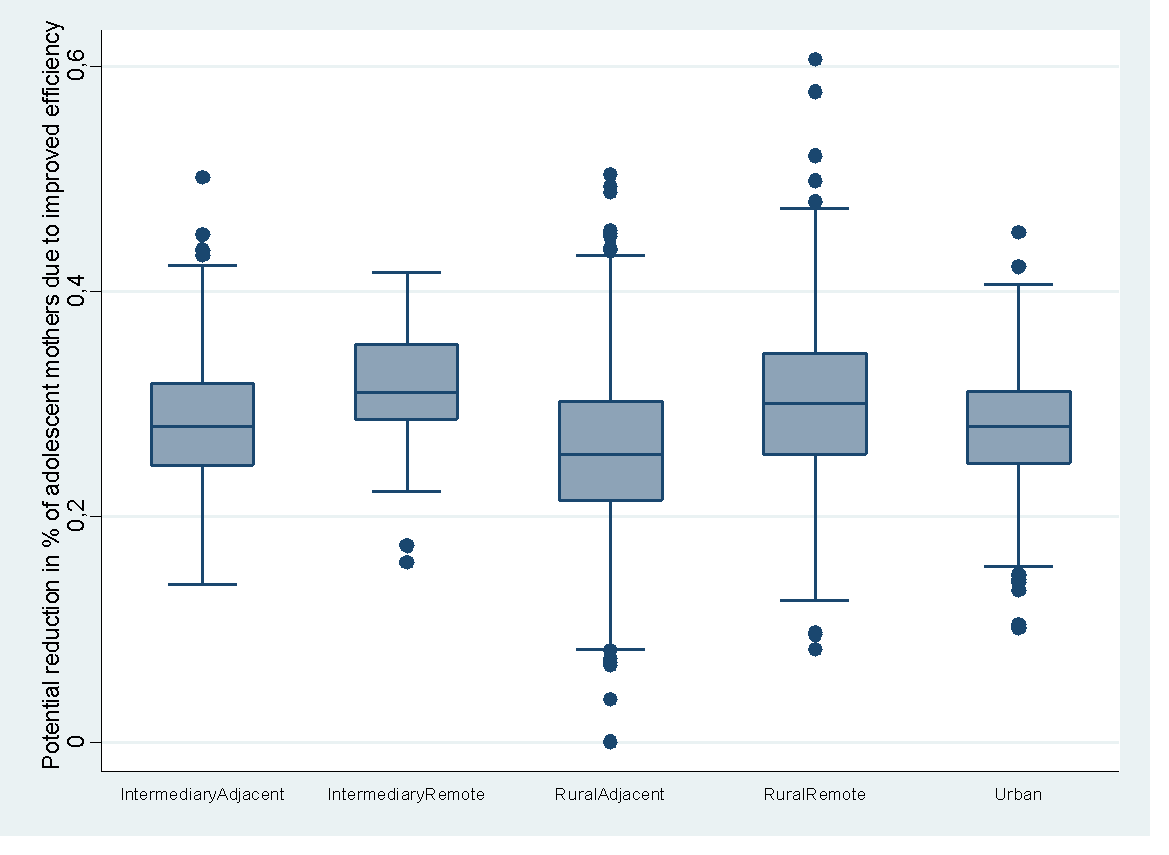


**Graph S7.10** - Reduction in the proportion of teenage mothers attributed to improved efficiency, according to rural-urban typologies and Federative Units


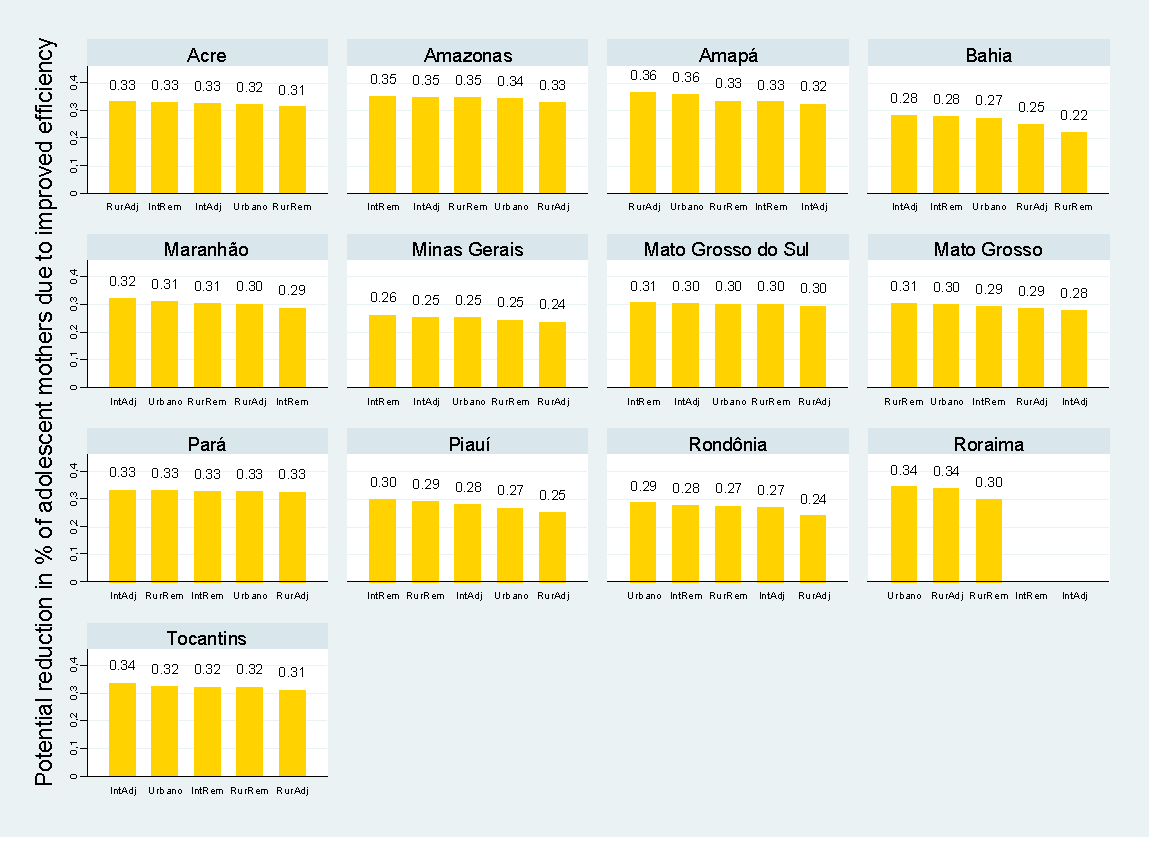


**Ranking of the Brazilian states, according to rural-urban typology**

Chart S7.1 shows the ranking of the Federal units from the efficiency evaluation, according to the IBGE typology: Maranhão, Tocantins, Bahia, Minas Gerais, and the Amazon states of Acre, Roraima, and Pará are in first place for the Remote Rural typology. In second place overall, the Adjacent Rural typology shows the best classification for the states of Amazonas, Piauí, and Rondônia. Then, in third place overall, the Remote Intermediate typology has the best rating for Amapá, Mato Grosso, and Mato Grosso do Sul (tied with Remote Rural). Lastly, the Adjacent Intermediate and Urban typologies remained in fourth and fifth place, respectively, and were not the best classification for any state. Alternatively, the states of Acre, Amapá, and Rondônia had the second-best ranking for the adjacent intermediate typology. Although the states of Amazonas, Pará, Mato Grosso do Sul, and Roraima scored slightly higher in the urban typology, they remained in last place.

**Chart S7.1** – Summary ranking measures, by Federal unit and rural-urban typology

| **State** | **Remote Rural** | **Urban** | **Adjacent Rural** | **Remote Intermediate** | **Adjacent Intermediate** |
| --- | --- | --- | --- | --- | --- |
| Acre (N) | **1,8 (1)** | **4,2** | **3,6** | **2,8** | **2,6 (2)** |
| Amazonas (N) | **2,4 (2)** | **3,6** | **2,0 (1)** | **3,0** | **4,0** |
| Amapá (N) | **3 (3)** | **4,8** | **3,8** | **1,6 (1)** | **1,8 (2)** |
| Bahia (NE) | **1,2 (1)** | **4,0** | **2,2** | **3,8** | **3,8** |
| Maranhão (NE) | **1,6 (1)** | **4,0** | **2,2** | **3,8** | **3,4** |
| Minas Gerais (SE) | **1,6 (1)** | **4,2** | **2,4** | **3,2** | **3,6** |
| Mato Grosso Sul (MW) | **2,6 (1,5)** | **3,6** | **2,8** | **2,6 (1,5)** | **3,4** |
| Mato Grosso (MW) | **2,6 (3)** | **4,0** | **2,4 (2)** | **2,2 (1)** | **3,8** |
| Pará (N) | **2,2 (1)** | **3,4** | **3,0** | **3,2** | **3,2** |
| Piauí (NE) | **2,6 (2)** | **4,2** | **2,0 (1)** | **2,8** | **3,4** |
| Rondônia (N) | **2,8 (3)** | **4,4** | **1,8 (1)** | **3,8** | **2,2 (2)** |
| Roraima (N) | **2,2 (1)** | **3,8** | **3,0** |  |  |
| Tocantins (N) | **1,8 (1)** | **4,0** | **2,8** | **2,8** | **3,4** |
| **Average rank** | **2,2 (1)** | **4,0 (5)** | **2,6 (2)** | **3,0 (3)** | **3,2 (4)** |

Note: Regions-MW-Midwest; N-North; NE-Northeast; SE-Southeast
