# Supplementary material for: Intersectional equity in Brazil’s remote rural municipalities: the road to efficiency and effectiveness in local health systems
Source: Front Public Health. 2024 Sep 10;12:1401193. doi: 10.3389/fpubh.2024.1401193 (PMC11419982; doi:10.3389/fpubh.2024.1401193)
Supplement: Supplementary file 8 [file Table_8.DOCX]

**Supplement 8-Summary chart of variables in the final models for the three analysis and metadata**

**Chart S8.1 – Summary of all analysis carried out and remaining variables in the final models**

| **Comprehensiveness** | | **All states (FU)** | | **Federative units (FU) only with Remote Rural Locations-RLL** | | | | | **RRL** | | **Remaining variables in the final models** |
| --- | --- | --- | --- | --- | --- | --- | --- | --- | --- | --- | --- |
| **Dimensions** | **Remaining variables in the final models** | **LEB** | **IM** | **LEB** | **IM** | **Preventable deathss** | **Low birth weight** | **Adolescent mothers** | **LEB** | **IM** |  |
| **Resources** | **Health expend % GDP** | **x** |  |  |  |  |  |  |  |  | **Health expend % GDP** |
|  | **PHC % GDP** | **x** | **x** |  |  |  |  |  |  |  | **PHC % GDP** |
|  | **Services % GDP** |  |  |  |  |  |  |  | **x** | **x** | **Services % GDP** |
|  | **Local health exp pc** |  |  | **x** | **x** |  | **x** |  |  |  | **Local health exp pc** |
|  | **Intragovern transfers** |  |  |  |  |  |  | **x** |  | **x** | **Intragov transfers** |
|  | **Density of nurses** |  | **x** |  |  |  |  |  |  |  | **Density of nurses** |
|  | **Density of PHC doctors** |  |  |  |  |  | **x** | **x** |  |  | **Density of PHC doctors** |
|  | **FHS coverage** |  |  | **x** | **x** |  |  |  |  |  | **FHS coverage** |
|  | **% FHS/OH teams** |  |  |  |  | **x** |  |  |  |  | **% FHS/OH teams** |
|  | **Riverine/Fluvial teams** |  |  |  |  |  |  |  | **x** | **x** | **Riverine/Fluvial teams** |
|  | **% CHW teams** |  |  |  |  | **x** |  |  |  |  | **% CHW teams** |
|  | **Availability Diagn/Ther** |  |  |  |  |  |  |  | **x** | **x** | **Availability Diagn/Ther** |
| **Products and Services** | **Incidence TB** | **x** |  |  |  |  |  |  |  |  | **Incidence TB** |
|  | **Use of services/needs** | **x** | **x** |  |  |  |  |  |  |  | **Utilization serv/needs** |
|  | **% vaginal deliveries** | **x** |  |  |  |  |  |  |  |  | **% vaginal deliveries** |
|  | **Hospitalization sens BS** |  | **x** |  |  |  |  |  |  |  | **Hospitalization sens BS** |
|  | **Prenatal appointments** |  |  | **x** | **x** |  |  |  |  |  | **Prenatal appointments** |
|  | **Adolescent mothers** |  |  |  | **x** |  |  |  | **x** | **x** | **Adolescent mothers** |
|  | **Low birth weight** |  |  |  |  | **x** |  |  |  |  | **Low birth weight** |
|  | **Home visits** |  |  |  |  |  | **x** |  |  |  | **Home visits** |
|  | **% ARI/diarrhea <2a** |  |  |  |  | **x** |  |  |  |  | **% ARI/diarrhea <2a** |
| **Intersectoral** | **% pop<1/4 MW** | **x** | **x** |  |  |  |  |  |  |  | **% pop<1/4 MW** |
|  | **Unemployment** | **x** | **x** |  | **x** | **x** |  |  |  |  | **Unemployment** |
|  | **Gini Index** |  | **x** | **x** | **x** |  |  | **x** | **x** |  | **Gini Index** |
|  | **Average income** |  |  |  | **x** |  |  | **x** |  |  | **Average income** |
|  | **Ratio ethnicity income** | **x** |  |  |  |  |  |  |  |  | **Ratio ethnicity income** |
|  | **Illiteracy** | **x** | **x** | **x** | **x** |  |  |  | **x** | **x** | **Illiteracy** |
|  | **% highly educated** |  |  |  |  |  |  | **x** |  |  | **% highly educated** |
|  | **Ratio ethnicity educ** |  | **x** |  |  |  |  |  |  |  | **Ratio ethnicity educ** |
|  | **Ratio EG education** |  |  |  |  |  |  |  | **x** | **x** | **Ratio EG education** |
|  | **Basic Sanitation** | **x** | **x** | **x** | **x** |  |  |  | **x** | **x** | **Basic Sanitation** |
|  | **Smoking** | **x** | **x** |  |  |  |  |  |  |  | **Smoking** |

**Note:** GDP-Gross Domestic product; PHC-Primary Health Care; FHS-Family Health Strategy; CHW-Community Health Workers; TB-Tuberculosis; BS-Basic Sanitation; ARI-Acute Respiratoy Infections; MW-Minimum wage;

EG-Ethnicity and Gender.

**Chart S8.2**-Metadata Dimensions, subdimensions, variables, formulas, unit of measurement, sources and links

| **Dimension** | **subdimension** | **Variable name** | **Formula** | **Unit of measure-ment** | **Source** | **Link** |
| --- | --- | --- | --- | --- | --- | --- |
| Inputs | Financial Resources | DPC -local health expenditures per capita | Local health expenditure per capita in R$ (committed and settled) | R$ | SIOPS  (Public Health budget information system) | https://www.gov.br/saude/pt-br/acesso-a-informacao/siops http://siops-asp.datasus.gov.br/CGI/deftohtm.exe?SIOPS/serhist/municipio/mIndicadores.def |
| Inputs | Financial Resources | TIG –intergovernamental transfers | Total intergovernmental transfers (in R$) | R$ | SIOPS | https://www.gov.br/saude/pt-br/acesso-a-informacao/siops |
| Inputs | Financial Resources | State health spending as a % of GDP | Current spending on health by the state/total state GDP | R$ | SIOPS | https://www.gov.br/saude/pt-br/acesso-a-informacao/siops |
| Inputs | Financial Resources | % GDP of Public Administration | Percentage share of public administration value added in total value added | % | IBGE-Brazilian Institute of Geography and Statistics | https://sidra.ibge.gov.br/pesquisa/pib-munic/tabelas |
| Inputs | Financial Resources | % GDP - Services | Percentage share of added value from trade and services in total added value | % | IBGE | https://sidra.ibge.gov.br/pesquisa/pib-munic/tabelas |
| Inputs | Human Resources | FHS coverage-  FHS-Family Health Strategy | Population covered by the Family Health Strategy/total population | % | e-Gestor  e-Manager | https://egestorab.saude.gov.br/ |
| Inputs | Human Resources | % FHS teams SB-Oral Health  FHS-Family Health Strategy | % of Family Health or Oral Health teams out of total teams | % | Datasus/  CNES | https://datasus.saude.gov.br/informacoes-de-saude-tabnet/ |
| Inputs | Human Resources | % Riverine and Fluvial teams | % of riverine/fluvial teams out of total teams | % | Datasus/  CNES  National Registry of Health Facilities; Information Technology Department of the Public Healthcare system | https://datasus.saude.gov.br/informacoes-de-saude-tabnet/ |
| Inputs | Human Resources | % of CHW – Community Health Workers | % of CHWs out of total teams | % | e-Gestor | https://egestorab.saude.gov.br/ |
| Inputs | Human Resources | CHW coverage  CHW – Community Health Workers | Population covered by CHWs/total population | % | Datasus/  CNES | https://datasus.saude.gov.br/informacoes-de-saude-tabnet/ |

| **Dimension** | **subdimension** | **Variable name** | **Formula** | **Unit of measurement** | **Source** | **Link** |
| --- | --- | --- | --- | --- | --- | --- |
| Inputs | Human Resources | Density of nurses | number of nurses per thousand inhabitants | 1,000 | Datasus/  CNES | https://datasus.saude.gov.br/informacoes-de-saude-tabnet/ |
| Inputs | Human Resources | Density of PHC doctors | number of doctors per thousand inhabitants (basic areas; clinical/general practitioner/FHS or CF/pediatrician/GO) | 1,000 | Datasus/  CNES | https://datasus.saude.gov.br/informacoes-de-saude-tabnet/ |
| Inputs | Material Resources | Availability of Diagnostic and Therapeutic Support Services | Total diagnostic and therapeutic support equipment/total equipment | % | Datasus/  CNES | https://datasus.saude.gov.br/informacoes-de-saude-tabnet/ |
| Products | Prevention | Incidence of Tuberculosis | New cases of tuberculosis/general population | 1,000 | Datasus-SINAN (Compulsoy notifiable diseases information system) | https://datasus.saude.gov.br/informacoes-de-saude-tabnet/ |
| Outputs | Access/utilization | Adequate Prenatal according to number of appointments | Total pregnant women with seven or more prenatal consultations/total pregnant women | % | Datasus-SINASC (Live births Information System) | https://datasus.saude.gov.br/informacoes-de-saude-tabnet/ |
| Outputs | Access/utilization | % of vaginal births | Total live births to resident mothers by normal delivery/total live births | % | Datasus-SINASC | https://datasus.saude.gov.br/informacoes-de-saude-tabnet/ |
| Outputs | Access/utilization | % de ARI/Diarrhea in children <2y | Children under two diagnosed with ARI or diarrhea/total children under two years of age | % | Datasus-SIAB (PHC Information system) | https://datasus.saude.gov.br/informacoes-de-saude-tabnet/ |
| Outputs | Access/utilization | Home visits Famiy Healthr | Total family home visits/population | % | Datasus-SIAB/SISAB | https://datasus.saude.gov.br/informacoes-de-saude-tabnet/ |
| Outputs | Intermediate Results | Hospitalizations due to basic sanitation-sensitive causes | Hospitalizations due to diseases transmitted by insect vectors, fecal-oral route and water contamination or lack of hygiene | 1,000 | SNIS- National System of Sanitation Information | <https://www.gov.br/mdr/pt-br/assuntos/saneamento/snis> |
|  | Intermediate Results | % adolescent mothesr | Number of live births to mothers under 20/total live births to resident mothers | % | Datasus-SINASC | https://datasus.saude.gov.br/informacoes-de-saude-tabnet/ |

| **Dimension** | **subdimension** | **Variable name** | **Formula** | **Unit of measurement** | **Source** | **Link** |
| --- | --- | --- | --- | --- | --- | --- |
| Outputs | Intermediate Results | % Low birth weight | Live births newborns weighing less than 2,500 g/total live births to resident mothers | 1,000 | Datasus-SINASC | https://datasus.saude.gov.br/informacoes-de-saude-tabnet/ |
| Outputs | Intermediate Results | Probability of deaths due to preventable causes | It includes long lists of causes, according to the following ranges: <5 years and 5-74 years and includes causes that can be avoided by health promotion (against infectious diseases and accidents) and primary prevention (vaccinations and prenatal care, childbirth and puerperium) or by adequate treatment and control (secondary prevention, chronic diseases). | 1,000 | Datasus-SIM  National Health Statistics – Mortality Information System | https://datasus.saude.gov.br/informacoes-de-saude-tabnet/ |
| Outputs | Utilization according to health needs | Health services utilization according to health needs | The use of health services at state level, according to health needs, was calculated using regressions in which the use of services, general health status and limitations due to chronic diseases were assessed, adjusted for gender, race, marital status, education and income. Age, the existence of chronic diseases (collinear) and registration in the FHS units were not significant, although they were tested. | number | IBGE-PNS 2013/19  National Health Survey-Brazilian Institute of Geography and Statistics | https://sidra.ibge.gov.br/pesquisa/pns |
| Outcomes | Health levels | Infant Mortality (rate) | Deaths in children under one year old/total live births | 1.000 | Datasus-SIM and SINASC (Live births Information System) | https://datasus.saude.gov.br/informacoes-de-saude-tabnet/ |
| Outcomes | Health levels | Life Expectancy at birth | Life expectancy at birth, according to life tables | number | UNDP- United Nations Development Programme | <https://www.undp.org/pt/brazil/idhm-munic%C3%ADpios-2010> |

| **Dimension** | **subdimension** | **Variable name** | **Formula** | **Unit of measurement** | **Source** | **Link** |
| --- | --- | --- | --- | --- | --- | --- |
| Intersectoral | Poverty | % of population earning <1/4 Minimum Wage | People earning less than 1/4 of the minimum wage/Total population | % | IBGE-census | https://sidra.ibge.gov.br/home/pms/brasil |
| Intersectoral | income inequalities | Gini Index | Value of the Gini Index of people's per capita household income | Index (0-1) | IBGE-census | https://sidra.ibge.gov.br/home/pms/brasil |
| Intersectoral | Income and work | Average Income | Average per capita income of employed people | R$ | IBGE-census | https://sidra.ibge.gov.br/home/pms/brasil |
| Intersectoral | Income and work | Ethnicity-income ratio | Average income among whites/Average income among blacks and browns | number | IBGE-census | https://sidra.ibge.gov.br/home/pms/brasil |
| Intersectoral | Income and work | % unemployed | % of economically active people aged 16 and over who are not employed | % | IBGE-census | https://sidra.ibge.gov.br/home/pms/brasil |
| Intersectoral | Education | Illiteracy | % of people aged 15 and over who can neither read nor write | % | IBGE-census | https://sidra.ibge.gov.br/home/pms/brasil |
| Intersectoral | Education | % highly educated | People aged 18 with completed secondary education/total people aged 18 | % | IBGE-census | https://sidra.ibge.gov.br/home/pms/brasil |
| Intersectoral | Education | Ethnicity/gender ratio % highly educated | % black and brown people with high schooling/% white people with high schooling | number | IBGE-census | https://sidra.ibge.gov.br/home/pms/brasil |
| Intersectoral | Education | Ethnicity/gender ratio % highly educated | % highly educated black and brown women/% highly educated white men | number | IBGE-census | https://sidra.ibge.gov.br/home/pms/brasil |
| Intersectoral | Development | MHDI - Municipal Human Development Index | Municipal Human Development Index Geometric mean of the indices of the Income, Education and Longevity dimensions, with equal weights, in 2010. | Índice (0-1) | UNDP | https://sidra.ibge.gov.br/home/pms/brasil |
| Environment | Basic Sanitation | Percentage of people living in households with access to water supply (2010) | People living in permanent private households served with or without household plumbing, in 2010/Total number of people living in permanent private households, in 2010 | % | IBGE-census | https://sidra.ibge.gov.br/home/pms/brasil |

| **Dimension** | **subdimension** | **Variable name** | **Formula** | **Unit of measurement** | **Source** | **Link** |
| --- | --- | --- | --- | --- | --- | --- |
| Environment | Basic Sanitation | Percentage of people living in households with access to sanitation (2010) | People living in permanent private households who have an outlet for their waste by connecting the household to the septic tank, in 2010/Total number of people living in permanent private households, in 2010 | % | IBGE-census | https://sidra.ibge.gov.br/home/pms/brasil |
| Intersectoral | Governance | Transparency index (MPF) | Level of transparency conferred by an index relating to the following items: general, income and expenditure, tenders and contracts, reports, citizen information services and electronic services (e-SIC); disclosure of the structure and form of contact and good transparency practices (score ranges from 0-10) | number | MPF-Federal Public Ministry | <http://combateacorrupcao.mpf.mp.br/ranking/pontuacao/ranking/itens-avaliados> |

**Sources:** CNES - National Register of Health Establishments; SIA - Outpatient Information System; SIAB/SISAB - Primary Care Information System; SIH - Hospital Information System; SINAN - Notifiable Diseases Information System; SINASC - Live Births Information System; SIM - Mortality Information System; SISCOLO and SISMAMA - cervical and breast cancer information systems; PNI - National Immunization Program; DATASUS- Information Technology Department of the Public Healthcare system; IBGE- Brazilian Institute of Geography and Statistics, SIOPS/MS-Information System for Public Budget in Health/Ministry of Health; STN/MF - National Treasury Secretary/ Ministry of Finance; UNDP-United Nations’ Development Programme/Brazil and MPF -Federal Public Ministry; PNS-National Health Survey; SNIS- National System of Sanitation Information; e-Gestor-PHC information (e-Manager)
